# Endocidal Regulation of Secondary Metabolites in the Producing Organisms

**DOI:** 10.1038/srep29315

**Published:** 2016-07-08

**Authors:** Shiyou Li, Ping Wang, Wei Yuan, Zushang Su, Steven H. Bullard

**Affiliations:** 1 National Center for Pharmaceutical Crops, Arthur Temple College of Forestry and Agriculture, Stephen F. Austin State University, Nacogdoches, TX 75962, USA

## Abstract

Secondary metabolites are defined as organic compounds that are not directly involved in the normal growth, development, and reproduction of an organism. They are widely believed to be responsible for interactions between the producing organism and its environment, with the producer avoiding their toxicities. In our experiments, however, none of the randomly selected 44 species representing different groups of plants and insects can avoid autotoxicity by its endogenous metabolites once made available. We coined the term endocides (endogenous biocides) to describe such metabolites that can poison or inhibit the parent via induced biosynthesis or external applications. Dosage-dependent endocides can selectively induce morphological mutations in the parent organism (e.g., shrubbiness/dwarfism, pleiocotyly, abnormal leaf morphogenesis, disturbed phyllotaxis, fasciated stems, and variegation in plants), inhibit its growth, development, and reproduction and cause death than non-closely related species. The propagule, as well as the organism itself contains or produces adequate endocides to kill itself.

Secondary metabolites (SMs) usually refer to the organic compounds that are not directly involved in the normal growth, development, and reproduction of an organism^1^,^2^,^3^. Some authors have suggested that SMs may have no explicit role in the internal economy of the producing organism^4^, but it is usually believed that they are responsible for interactions between the producing organism and its environment, particularly in defense^1^,^3^,^5^,^6^,^7^,^8^,^9^,^10^. To refer to biochemical interactions between plants, in 1937, H. Molisch coined the term allelopathy, which was later defined as any direct or indirect, stimulatory and inhibitory effect by one plant (including microorganisms) on another through production of chemical compounds that escape into the environment^11^. Allelopathy is usually interspecific^12^. Intraspecific allelopathy, commonly known as autotoxicity occurs when a plant releases toxic chemical substances into the environment that inhibit germination and growth of the same plant species^12^,^13^. Since the 1970s, such an exogenous autotoxicity has drawn great interests of scientists from various fields. Recent evidences have indicated that many autotoxicity cases are primarily caused by the indirect effects of autotoxins via influencing microbes or parasitic organisms in the environment^14^,^15^,^16^,^17^. Some identified autotoxins or allelochemicals are not necessarily responsible for allelopathy because they may not reach sufficient concentrations and duration in soils to display direct inhibitory effects on their neighbors^17^. Endogenous autotoxicity in producers induced by their own SMs has never been seriously addressed^18^. In fact, it has been widely believed that a species can avoid self-toxicity by its own toxic metabolites and thus many studies have focused on organisms’ avoidance and detoxification mechanisms^19^,^20^,^21^,^22^,^23^,^24^,^25^,^26^,^27^,^28^.

To reveal the internal role of some SMs in their producers, we investigated 44 species representing different groups of plants and insects found in the Southeastern United States. It was found that no organism can avoid either endogenous or exogenous autotoxicity by its own metabolites once made available via induced biosynthesis or external applications. The fact of unavoidance and commonness of endogenous autotoxicity in the producing organism induced by its endocides does not support the common knowledge that a species can avoid self-toxicity by its own toxic metabolites^19^,^20^,^21^,^22^,^23^. We further found that these agents were usually more toxic to the producing species and its closely-related species than to others. This phenomenon cannot be explained by allelopathy, defense or any other existing theory. Thus, we coined the new term *endocides* (endogenous biocide) to describe such selective toxic SMs that cause both endogenous and exogenous autotoxicity.

## Results

### Morphological Mutations Induced by Prolonged Soaking of Fruits (Seeds) in Water

The prolonged soaking of fruits (seeds) in water induced abnormal morphogenesis in each of the 12 woody and herbaceous species investigated (Supplementary Table S1).

**Figure 1:**
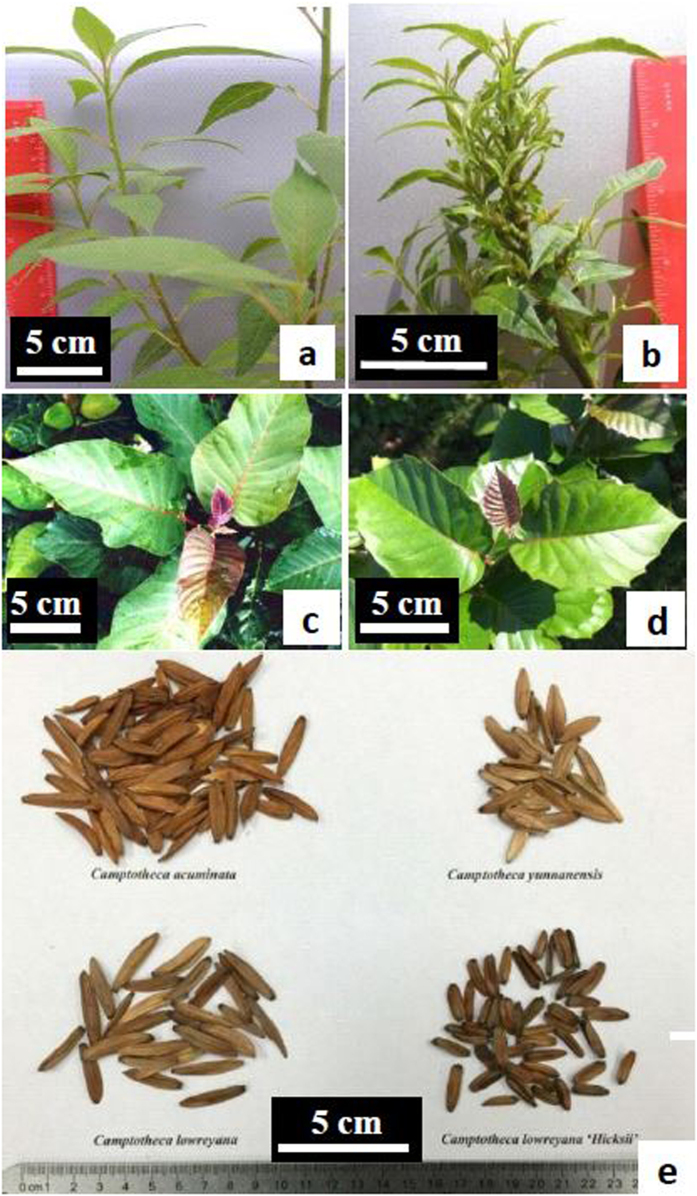
Comparison of *Camptotheca lowreyana* cultivars with its parent *C. lowreyana* var*. lowreyana*. (**a**) Stems and leaves of ‘Katie’. (**b**) Fasciated stems, heterogeneous leaves, reduced internodes, and disturbed phyllotaxis of ‘CT168’. (**c**) Leaves on a lower branch of mature tree of *C. lowreyana* var. *lowreyana.* (**d**) Leaves on a lower branch of mature tree of ‘Hicksii’. (**c**) Comparison of mature fruits of different *Camptotheca* taxa: ‘Hicksii’ has the smallest fruits (upper row: left-*C. acuminata* and right-*C. yunnanensis*; bottom row: left-*C. lowreyana* var. *lowreyana* and right-‘Hicksii’).

Without any treatment, none of the total 422 seedlings of *Camptotheca lowreyana* developed any abnormal leaves (vs those grown in the native range of China). Following a 9-week prolonged soaking in water, 23 of the total 69 seedlings had morphological mutations in at least one true leaf or stem. The mutations include leaf size, lobed or bifid leaves, compound leaves (e.g., two leaflets per petiole), disturbed phyllotaxis, fasciated stems, or leaf variegation (with white and green bi-color or mosaic pattern). The propagation of two mutated seedlings by shoot cutting led the development of cultivar ‘Katie’ and ‘Hicksii’, respectively^29^. Unlike the parent tree that grows up to 20 m in height, ‘Katie’ is a shrub with a maximum height of 3 m (Fig. 1). It has a vigorous and dense multi-branching growth habit and small lanceolate or elliptic leaves with entire margins in both juvenile and mature stages^30^. ‘Hicksii’ has shorter fruits and smaller cordate leaves with large-tooth leaf margins particularly in juvenile growth stage in comparison with its patent (Table 1). Seedlings germinated from the fruits of ‘Hicksii’ trees are morphologically consistent with their parents. This is desirable because both cultivars have a higher yield of anti-cancer camptothecin (CPT) in young leaves (0.4778% in ‘Katie’ and 0.5537% in ‘Hicksii’ vs. 0.3913% in *C. lowreyana*, on dry weight basis), and are more hardy and drought-tolerant than natural taxa of *Camptotheca*^29^.

*Camptotheca* seedlings germinated from the fruits without soaking treatment did not develop any abnormal leaves. After three months of saturated soaking of fruits by rain water, however, about 92.3% of the ‘Hicksii’ seedlings and about 7.9% of *C. acuminata* seedlings germinated under the parent trees had abnormal morphogenesis in at least one true leaf (Supplementary Fig. S1 and Table S2). For both species, 24 h fruit soaking in water in the laboratory experiment resulted in significantly higher germination rates than either control (no soaking) or prolonged (4 weeks) soaking group (*P* < 0.05) (Supplementary Fig. S2). Following a 4-week fruit soaking in water, however, 15.6% *C. acuminata* seedlings developed 2–3 branches and 38.5% ‘Hicksii’ seedlings developed 2–5 branches from the main stem in comparison with no branch development during the first 2–3 months of normal seedling growth.

**Figure 2:**
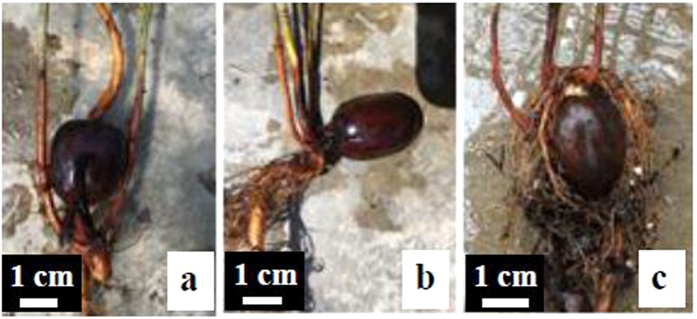
Shrubbiness development (multiple stems directly from the same radicle) of three *Quercus* species after a month-long soaking of the bulk acorns in an amount of water just adequate water to cover all acorns for about a month. (**a**) 7.3% of the seedlings germinated from the treated acorns of *Quercus shumardii* developed shrubbiness. (**b**) 8.6% of the seedlings germinated from the treated acorns of *Q. texana* developed shrubbiness. (**c**) 19.3% of the seedlings germinated from the treated acorns of *Q. michauxii* developed shrubbiness.

Similar to *Camptotheca, Quercus* might develop abnormal morphogenesis after prolonged soaking of acorns. After a month long soaking of bulk acorns in just enough water to cover all acorns, 7.32% of *Q. shumardii*, 8.64% of *Q. texana*, and 19.27% of *Q. michauxii* seedlings developed 2–3 stems directly from the same radicle (shrubbiness) (Fig. 2). Bilobed, bifid, or variegated leaves were also found in some seedlings of each species. None of the seedlings germinated from unsoaked acorns developed multiple stems or abnormal leaves.

**Figure 3:**
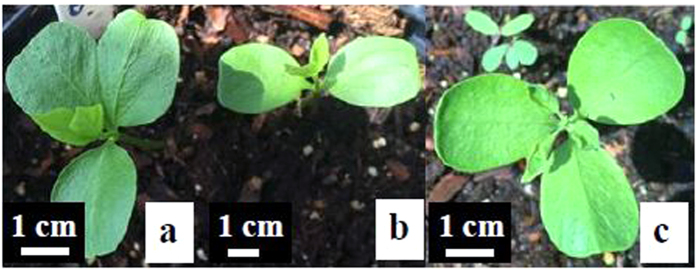
Induced pleiocotyly in *Triadica sebifera* by the prolonged seed soaking in water for six weeks. (**a**) Cotyledon with two lobes (two fused cotyledons). (**b**) Normal cotyledon. (**c**) Tricotyledon.

57.8% of *Triadica sebifera* seeds soaked in water for six weeks germinated. 10.6% of the seedlings had three or four cotyledons (pleiocotyly or polycotyly) and cotyledons with two lobes (which may be interpreted as two fused cotyledons). In comparison, the seedlings germinated from untreated seeds developed with normal dicotyledons (Fig. 3).

### Morphological Mutations Induced by External Applications of Extracts

**Figure 4:**
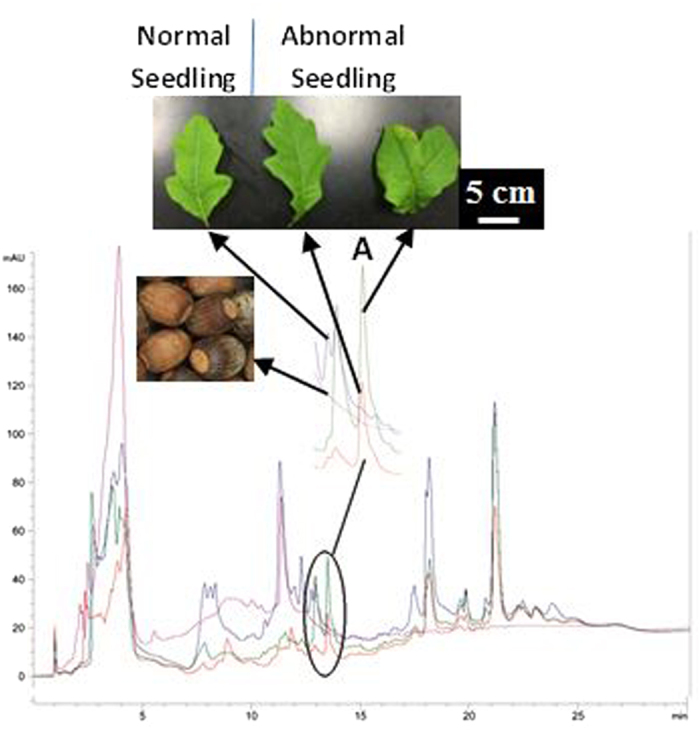
HPLC profiles of leaf extracts of abnormal seedling of *Quercus shumardii* induced by the acorn extracts in comparison with leaf extracts of a normal seedling and acorn extracts. A: A unique compound in the abnormal seedling.

The induced morphological mutations in *Q. shumardii* seedlings germinated from acorns soaked by *Q. shumardii* acorn extracts for 48 h are similar to the prolonged water soaking of the acorns as described in the previous section. The application of 5% extracts of the acorns induced about 18% of the seedlings to develop 3–5 stems in comparison with the development of one main stem in the control group (no soaking treatment) (Supplementary Fig. S4). Further, early leaf development of almost all seedlings in the 0.5% or 5% extracts treatment group displayed abnormal morphogenesis (e.g., lobed or bifid leaves, or variegated leaves) (Supplementary Fig. S5). The HPLC profiles of leaf samples from two normal *Q. shumardii* seedlings are similar each other but different from either normal or bi-lobed leaves from the abnormal seedlings induced by 0.5% extracts. The chromatographs of the extracts also showed that *Q. shumardii* acorns had much less chemical diversity than seedlings. Interestingly, the normal and bi-lobed leaves from the abnormal seedlings (Supplementary Fig. S5e and S5f) are similar in HPLC profiles but both had a compound or compounds that were not detected in either acorns or normal seedlings of *Q. shumardii* (Fig. 4). The unique compound(s) are likely metabolic products of the abnormal oak seedlings.

External application of endocides also induced pleiocotyly. In the same experiment, pleiocotyly was observed in 13.9% of the *T. sebifera* seedlings germinated from the seeds soaked in 5% *T. sebifera* seed extracts for six weeks.

The seeds of *Arachis hypogaea* soaked in a 5% solution of *A. hypogaea* seed extracts for a week did not germinated. Four of the seven seedlings germinated from the seed treatment of 5% *A. hypogaea* shell extracts for a week and one of the 20 seedlings in the water soaking treatment for a week developed abnormal leaf morphogenesis including one or three developed leaflets (vs. normal four leaflets), lobed leaves, flat petioles, petioleless smaller leaflets with non-entire leaf margins (vs. normal larger leaflets with entire margin), or variegated leaves and fused stems (Supplementary Fig. S3).

Mutations were also observed when other propagules were treated with their extracts. Larger leaves (1.5 to 2.5 cm long) were developed and retained on new stems of *Opuntia ficus-indica* after the soaking treatment with a 5% solution of extracts of *O. ficus-indica* for 12 days. This is in contrast to the leaves of *O. ficus-indica* that are minute and are shed early in the normal development process.

**Figure 5:**
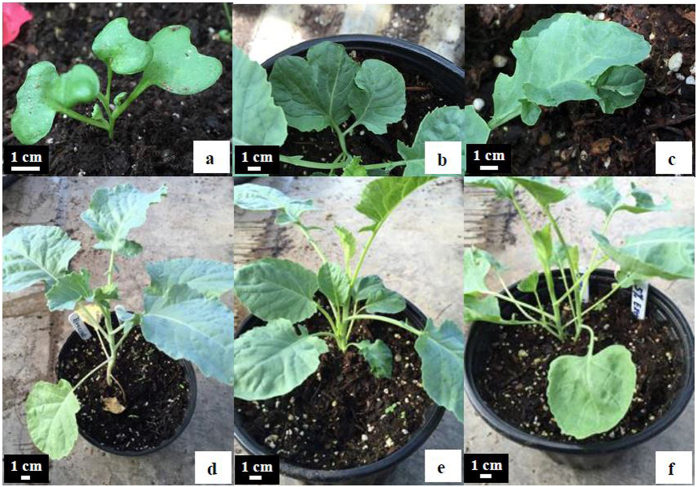
Induced morohological mutations in *Brassica oleracea* by soaking the seeds in 5% EtOH seed extracts for 48 h. (**a**) Abnormal pleiocotyly (4 cotyledons). (**b**) Leaf with two lobes. (**c**) Leaflet on leaf. (**d**) Normal single stem seedling without treatment. (**e**,**f**) Induced shrubbiness with two more stems.

Like woody species, herbaceous *Brassica oleracea* showed shrubbiness, pleiocotyly, and various abnormal leaves in seedlings germinated from the seeds soaked in a 5% solution of *B. oleracea* seed extracts for 48 h (Fig. 5). Of the 610 *B. oleracea* seedlings germinated from the 900 seeds soaked in a 5% solution of seed extracts for 48 h, approximately 1.3% developed 2–5 stems directly from the same radicle, approximately 1% had pleiocotyly, and approximately 3.5% had various abnormal leaf morphogenesis including leaves with two lobes or leaves with leaflets on surface. Following the 48 h water soaking, no seedling developed with multiple-stems or pleiocotyly and less than 0.5% of seedlings developed with an abnormal leaf. This is lower than the results observed in seedlings induced by the extracts.

### Morphological Mutations Induced by Decapitation Pruning

‘Katie’ plants underwent extensive pruning including decapitation to produce over 200 cuttings for propagations. One plant became a chimera with most stems having normal ‘Katie’ characteristics except one fasciated stem with smaller heterogeneous leaves in new growth. From this mutated stem, ‘CT168’, a dwarf mutant (up to 1 m tall in maturity) of ‘Katie’, was developed (Fig. 1). ‘CT168’ is characterized by its fasciated stems, smaller heterogeneous leaves, reduced internodes, and disturbed phyllotaxis. The dwarf has the highest CPT yield in young leaves among all *Camptotheca* taxa (0.5890%, on dry weight basis).

Similar chimeras with abnormal morphogenesis were observed in all simple-leaf species following decapitation pruning (Supplementary Fig. S6 and Table S3). *Sambucus canadensis* developed twice pinnately compound leaves with 3 leaflets after pruning. This stands in contrast to the normal once pinnately compound leaves with 5–11 leaflets.

### Inhibition and Elimination of the Producing Species by External Applications of Endocides

In all 37 testing species (Supplementary Table S4), the extracts and isolates can inhibit the growth (Figs 6 and 7; Supplementary Figs S7, S8, S11, S12, S14–S21, S23–S36, and S41–S52), reproduction (Supplementary Figs S22, S37–S40, S42, and S43) and prevention of new spread (Supplementary Fig. S13) of the producing species at lower dosages and can kill them at higher dosages.

*Chara vulgaris* extracts can inhibit, eliminate, and prevent *C. vulgaris* and other two green algal species within a week (Supplementary Fig. S7).

**Figure 6:**
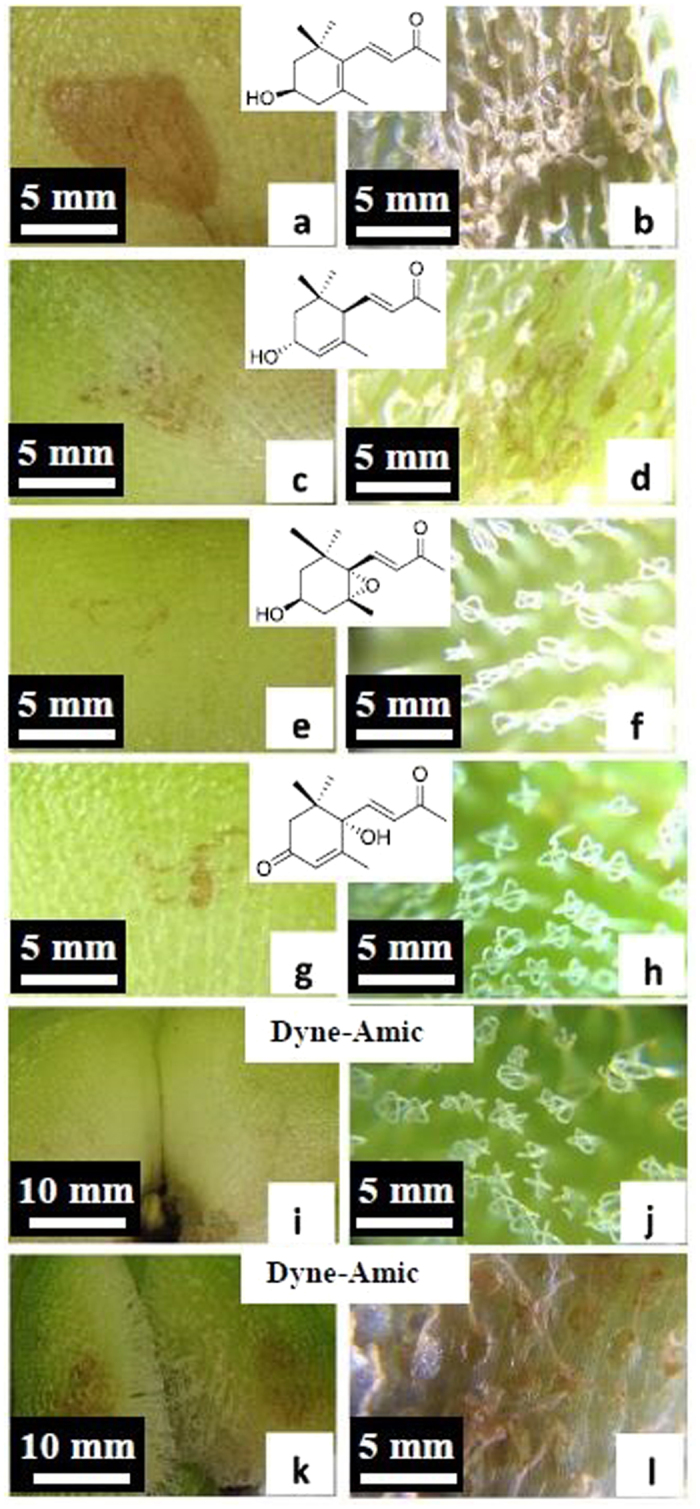
Toxicities of four isolated pure compounds from *Salvinia molesta* and surfactant (Dyne-Amic) on the upper and lower surfaces of *S. molesta*, respectively (application of 8 μL of testing agents dissolved in nanopure water at 1% concentration or surfactant at 0.5% concentration on the leaf surface of intact plants). (**a**,**b**) Application of (+)-3-hydroxy-*β*-ionone on the lower leaf surface killed the tissues ((**a**) lower surface; (**b**) upper surface). (**c**,**d**) Application of (3R, 6R, 7E)-3-hydroxy-4,7-megastigmadien-9-one on the lower leaf surface caused less damage on the tissues ((**c**) lower surface; (**d**) upper surface). (**e**,**f**) Application of annuionone D on the lower leaf surface had no observable damage on tissues ((**e**) lower surface; (**f**) upper surface). (**g**,**h**) Application of dehydrovomifoliol on the lower surfaces did not cause any observable leaf damages ((**g**) lower surface; (**h**) upper surface). (**i**–**l**) Application of Dyne-Amic: Dyne-Amic did not cause any damage when applied on lower surface (**i**) lower surface and (**j**) upper surface, but can destroy the tissue when applied on upper leaf surface (**k,l**).

Our laboratory, greenhouse, and field experiments showed that EtOH or water extracts of dried matter and fresh juice of *Salvinia molesta* can effectively inhibit and eliminate *S. molesta* and *S. minima* (Supplementary Figs S11–S15 and S17–S21). To date, we have identified and isolated at least four bioactive compounds from *S. molesta* that exhibits phytotoxicity on *S. molesta,* including salviniside II (Supplementary Figs S16 and S17) and (+)-3-hydroxy-*β*-ionone (Fig. 6). The growth of *S. molesta* was totally inhibited by salviniside II at a 0.1% of concentration within two weeks. There was no new growth identified thereafter during eight months of observation. The leaves of *S. molesta* treated with (+)-3-hydroxy-*β*-ionone at the dosage of 8 μL/leaf had injury within 12 h of the treatments, and two leaves were dead by the end of experiment. The leaves of *S. molesta* treated with other isolates from *S. molesta* at the same dosage had no or slight injury during two weeks of observation (Supplementary Table S6).

The application of 0.5% Dyne-Amic or 1% DMSO did not show toxicity on the lower leaf surface of *S. molesta*, but application of Dyne-Amic on the upper leaf surface may destroy the contact area of the leaf blades because the trichomes damaged by the surfactant released the endocides (Fig. 6).

**Figure 7:**
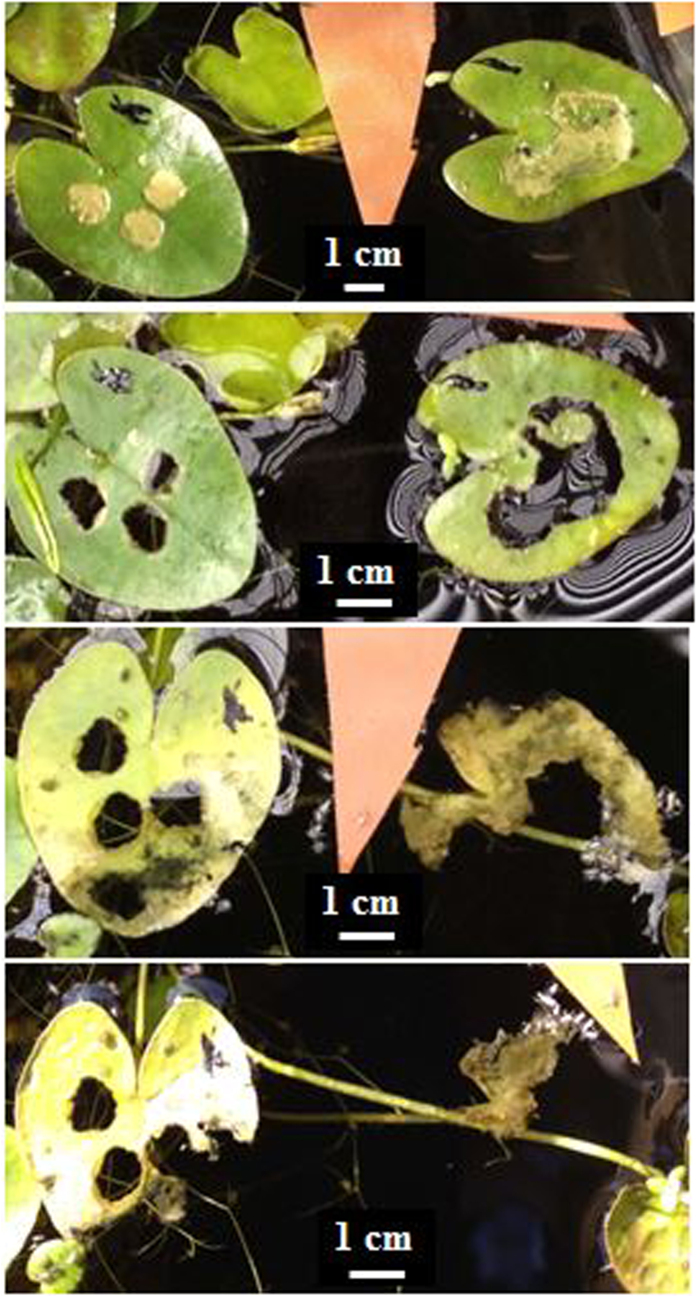
Leaf changes of *Nymphoides cristata* after the application of 10 μL fraction A of EtOH extracts of *N. cristata* (from top to bottom: 2, 4, 11, and 12 days after the treatment, respectively).

When applied in concentrations of 2.5% or higher, EtOH extracts of dried matter or fresh juice of *Nymphoides cristata* can inhibit and kill the leaves and stems of *N. cristata* within four weeks (Supplementary Figs S23 and S26). A fraction of the extracts killed *N. cristata* more quickly (Fig. 7).

**Figure 8:**
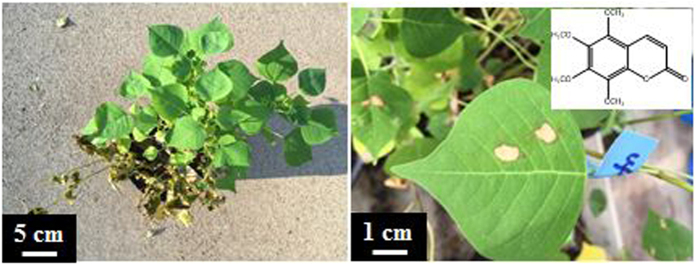
The photographs show that the impacts of 1% hexane fraction of the EtOH extracts of *Triadica sebifera* leaves and stems can kill 4-month-old seedlings of *T. sebifera* within 3 days (left) and the isolated 5,6,7,8-tetramethoxycoumarin showed endocidal activity by foliar application of 10 μL of 5% concentration (right).

In the field foliar spray tests, 10% extracts of *T. sebifera* leaves and stems can selectively inhibit and eliminate seedlings of the producing species without any effects on *Ligustrum sinense* and *Q. texana* (Supplementary Fig. S32). 1% Hexane fraction of the extracts was found to be more active than the 10% extracts and other fractions used in the foliar spray (Fig. 8). The 4-month-old seedlings of *T. sebifera* were injured within several hours and killed within several days following a foliar spray of 1% hexane fraction. None seedlings of the other tested species sustained any injury by the 5% hexane fraction of *T. sebifera.* In the droplet test of intact leaves, 5,6,7,8-tetramethoxycoumarin (Fig. 8) isolated from the hexane fraction showed endocidal activity against *T. sebifera*.

The seed germination of *Pinus koraiensis* can be totally inhibited by its seed extracts (Supplementary Fig. S22). Appromately 99.8% of the *B. oleracea* seeds soaked in 5% solution of the *B. oleracea* seed extracts for two weeks lost viability (Supplementary Information 2.9). The *T. sebifera* seeds treated by 5% extracts of *T. sebifera* seeds for six weeks had only a 20% germination rate in comparison with 28.9% for the seeds soaked in water for 24 h, 57.8% for the seeds soaked in water for six weeks, and 40.6% for seeds without soaking treatment. No germination of *T. sebifera* seeds occurred when treated with 5% leaf and stem extracts of *T. sebifera* for four weeks. The germination of *Phaseolus vulgaris* was affected by the bean extracts (Supplementary Fig. S37). Soaking the beans in 0.5% extracts can delay germination, while over 70% of the beans did not germinate after treatment in higher concentration (5%). Seeds of *A. hypogaea* treated by 5% *A. hypogaea* shell or seed extracts for a week had only 7.7% or zero germination, respectively compared with 22.3% germination rate in water soaking for a week and 73.3% in the control group (Supplementary Fig. S38). The seed germination of *Sorghum bicolor* can be inhibited after soaking in a 5 or 10% concentratioin of its extracts for 72 h (Supplementary Information 2.26.1).

The acorn germination of both *Q. shumardii* and *Q. texana* was greatly inhibited in 5% extracts of *Q. shumardii* acorns (Supplementary Fig. S39). The soaking of *Q. shumardii* acorns in 0.5% extracts did not inhibit the germination but induced about 10.7% of germinated acorns to develop secondary or more stems directly from the same radicle. This morphologically is similar to shrub seedling development rather than tree seedling development. About 41% of *Q. shumardii* acorns germinated after treatment with 5% extracts in comparison with a 59% germination rate of acorns without any treatments. We found that the seed germination of *Q. texana* is mainly inhibited by extracts of its seeds rather than extracts of its pericarps (Supplementary Fig. S40). Both root and shoot development of *Allium sativum* can be totally inhibited by the shredded juice of its cloves (Supplementary Fig. S42).

The soaking of succulent plant tissues in their own extracts can inhibit and eliminate the plant. Only two of six fleshy oval stems (pads or paddles) of *O. ficus-indica* survived after soaking in 5% leaf extracts (Supplementary Information 2.8). None of the six stems of *Cnidoscolus aconitifolius* was survived after soaking in 5% stem extracts (Supplementary Information 2.13).

Our experiments indicated that *Solenopsis invicta* can be killed by the extracts of *S. invicta* without using formic acid. EtOH extracts are particularly effective (Supplementary Figs S47–S49). However, the EtOH extracts (primarily piperidine alkaloids) of *S. invicta* had selective activity against the ants and showed no effects on *Reticulitermes flavipes*. Formic acid commonly occurs in ants (Formicidae), termites (Isoptera), and some other insects. The organic acid is accumulated in exocrine glands of these insects and serves as defensive weapon fighting against attackers. It can be as concentrated as 60% of the secretion of ants, and workers can contain as much as 2 mg each^31^. The acid is a known natural pesticide. Like *S. invicta, R. flavipes,* another formic acid producing species could not avoid toxicity of formic acid during external topical or fumigation application (Supplementary Figs S50 and S51). All adults of *Schistocerca americana* were dead within 24 h after the exposure to 10% EtOH *S. americana* extracts (Supplementary Fig. S52). 33–40% of *Tenebrio molitor* and 37–97% of *Zophobas morio* were dead when treated with the same species extracts at the dosage equivalent to the extraction yield per worm (Supplementary Information 2.34).

## Discussion

Our results indicated that endocides play a negative regulatory role in the producing organism to make sure it has correct growth, development, and reproduction during the normal process. Enhanced biosynthesis or external applications of endocides can induce mutations in the parent organism (e.g., shrubbiness/dwarfism, pleiocotyly, abnormal leaf morphogenesis, disturbed phyllotaxis, fasciated stems, and variegation in plants) and chemical biosynthesis and derivatization of some SMs. The excessive endocides can inhibit the growth, development, and reproduction and even cause death of the producing organism. None of the tested organisms can avoid endogenous autotoxicity by its endocides once available. An organism or propagule contains or produces adequate endocides to kill itself when externally applied. Endocides are usually more toxic to the producer than to non-closely related species. In fact, a non-closely related species may have antidotal action on exogenous autotoxicity of the producing organism induced by endocides.

### Induced Morphological Mutations by Availability or Enhanced Biosynthesis of Endocides

Soaking seeds in water before planting has long been used to stimulate germination. It is recommended to only soak seeds for 12 to 24 h and no more than 48 h for optimal germination^32^,^33^. Prolonged soaking in water (e.g., several weeks) is always avoided^32^,^33^. Soaking, particularly in a large volume of water or flowing water will leash some endocides away from the embryo and thus promote germination, but unconventional prolonged soaking of fruits (seeds) of plants in water will extract more endocides, making them available to embryos. The available endocides at high dosage can inhibit germination and even make propagules not viable. The available endocides at low dosage will allow propagules to reproduce but induce morphological mutations in propagated seedlings or siblings, e.g., shrubbiness, pleiocotyly and/or cotyledon with two lobes. To develop such mutations, it is necessary to soak propagules with minimum amount of water for an extended period of time at a lower temperature so early reproduction can be avoided and adequate but unfatal concentrations of endocides become available to the propagules. An example is that development of cultivars ‘Katie’ and ‘Hicksii by a prolonged soaking of *C. lowreyana* fruits.

Short-time soaking of a propagule in EtOH extracts of the producing organism can yield the same inhibitory effects on its germination or growth and induce similar morphological mutations as is found by prolonged soaking in water. This directly supports the notion that endocides are the causes of the observed inhibition and induced mutations. It also indicates endocides are a major factor in seed dormancy, germination, and development. In normal development, *in planta* germination of mature seeds on plants can be avoided by transport and accumulation of endocides. The secondary dormancy developed after seed/fruit harvest or dispersal is because the level of endocides is still too high. This dormancy may continue until the reduction of endocidal level by degrading or taking them away naturally or artificially. In other words, reduction of endocides is one of the necessary conditions for seed germination. Secondary dormancy can be induced by the availability of endocides (e.g., through prolonged water soaking or external applications of endocides).

**Figure 9:**
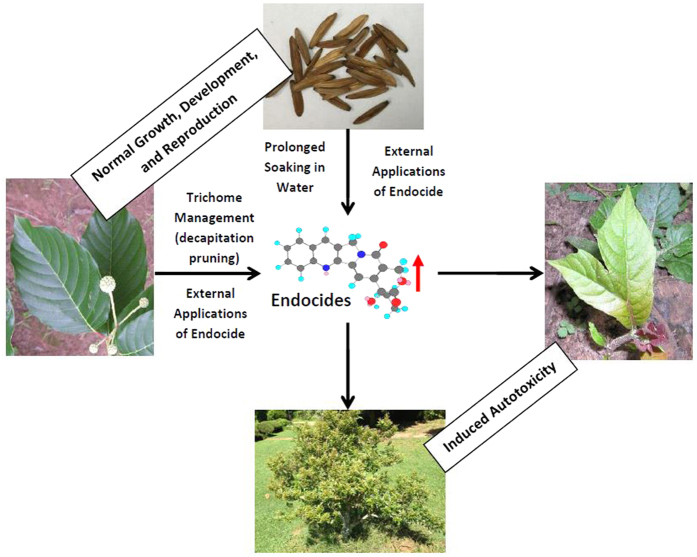
The diagram shows the endogenous autotoxicity in *Camptotheca* trees induced by the enhanced production or external applications of endocidal CPTs or prolonged periods of soaking the fruits in water or *Camptotheca* extracts. These endocide inductions or applications directly reduced apical dominance of *Camptotheca,* resulting in morphological mutations including shrubbiness in *Camptotheca* tree.

Interestingly, the abnormal morphogenesis in seedlings caused by prolonged soaking of propagules in water or external applications of endocides are similar to the reduced apical dominance and mutations caused by enhanced biosynthesis of endocides following decapitation pruning in woody species. Without disturbance, the fast-growing *Camptotheca* can avoid poisoning by its endogenous CPTs, potent DNA TOP1 inhibitors present at concentrations more than 10 times higher than the fatal level of exogenous application^18^. But *Camptotheca* cannot avoid endogenous autotoxicity of suddenly induced production of endocidal CPTs. Decapitation pruning can enhance the concentrations of CPT by several times in newly developed leaves and stems and thus induce endogenous autotoxicity in *C. acuminata*^18^,^34^,^35^. In addition to coppiced plant commonly developing multiple stems (suckering) in which leaf teeth development in the juvenile stage of seedlings, some dramatic deviations from normal morphogenesis such as serrated or lobed leaves, disturbed phyllotaxis, and fasciated stems can also be found due to induced endogenous autotoxicity^18^ (Fig. 9). *Camptotheca acuminata* resumes its normal morphogenesis when CPT is reduced to natural levels after discontinuing treatments^18^. Other woody species demonstrated similar lobed leaves and disturbed phyllotaxis following decapitation pruning as those observed in *Camptotheca.* The similarly bilobed leaves and other abnormalities caused by pruning cross different species may be caused by mutations in the same genes, such as *TOP1, FAS1*, and *FAS2*, as previously reported in some herbaceous plants^18^,^36^,^37^.

It is worth mentioning that the induced autotoxicity may include full mutations (e.g., shrubbiness) or partial mutations restricted to one or more specific tissues with the majority in normal development (chimeras). The distinct characters of full mutations are permanent for the muted individuals and may be fixed by vegetative propagation (e.g., development of shrub ‘Katie’ from the parent tree). The chimeras could also be good sources for development of desirable cultivars (e.g., ‘CT168’, a dwarf cultivar selected from ‘Katie’).

The induced mutations as described above could be observed occasionally in the field with extreme environmental condition. The documented autotoxicity of ‘Hicksii’ seedlings in the field was largely because the fruits had been soaked in water on the soil surface for over three months. During the period of January through March 2015, local rainfall reached more than 500 mm, about 50% more than the average, while the average temperature was about 3 °C lower than the average. In nature, abnormal morphogenesis in plants may be caused by enhanced *in planta* biosynthesis of endocides due to stresses or by exposure of propagules to high levels of endocides. Some observed shrubbiness of tree species or even herbaceous plants may be directly induced by endocidal change or availability. This phenomenon could occur in a place with a long period of saturated water before seed germination. However, this process involves many chance factors and thus it is impossible to make a prediction in nature.

### Induced Chemical Biosynthesis and Derivatization by Endocides

In addition to the enhanced yield of existing endocides, prolonged soaking in water, external applications of endocides, or decapitation pruning can increase chemical diversity of SMs in newly-developed tissues or seedlings. Decapitation pruning of *C. acuminata* trees induced biosynthesis of some alkaloids and triterpenoid glycosides that are not detected or occur at too low a level to be isolated in untreated plants^34^. The induced production of these minor or new compounds allowed us to identify and isolate two new derivatives of CPT^38^. Similarly, six and 12 new triterpenoid glycosides were isolated from the root and stem bark of *Cephalanthus occidentalis* and from fruits of *Aesculus pavia* following the pruning treatments, respectively^39^,^40^. In the present investigation, both normal and bi-lobed leaves from the abnormal *Q. shumardii* seedlings induced by 0.5% extracts of *Q. shumardii* acorns demonstrated a unique compound that was not detected in either acorns or normal seedlings of *Q. shumardii.* The induced chemical biosynthesis and derivatization by endocides is an important approach for enhancing yield of desirable SMs and producing novel molecules. The molecular mechanism of action remains unclear; however, we found auxins play a critical regulatory role in the induced autotoxicity of *Camptotheca*^18^.

### Unavoidance and Commonness of Endogenous Autotoxicity Induced by Endocides

In contrast to existing reports^24^,^25^,^26^,^27^,^41^, we found that all species (Supplementary Tables S1–S4) of plants and animals in our experiments cannot avoid autotoxicity through the external applications of their own extracts or isolates. Our data showed that the 5% EtOH extracts of *S. molesta* contain the amount of endocidal compound salviniside II equivalent to 0.12% of pure salviniside II in the solution. In order to exhibit the endocidal activity of active compounds (particularly minor compounds) in the tests, we thus used high concentrations (5% and even 10%) of crude extracts in the screenings.

These species represent 33 families of Charophyta, Bryophyta, Pteridophyta, Coniferophyta, Anthophyta, and Arthropoda. The plant species selected for extraction and external endocide experiments also represent various groups in terms of other classifications, such as life cycle or longevity (four annuals, one biennial and 27 perennials), habits (14 woody plants and 18 herbaceous species), loss of leaves (seven deciduous and five evergreen trees/shrubs), habitats (seven aquatic and 25 terrestrial species), water content of the environment (seven hydrophytes, one xerophyte, and 24 mesophytes), cultivation (seven food crops, one fruit, two pharmaceutical, and four timber crops), invesiness (17 invasive species and six native but unwanted species). The species selected in pruning experiments represent different habits and leaf types: seven trees, two shrubs, and one woody vine. *Sambacus canadensis* has pinnate compound leaves, while others in the experiment have simple leaves. For insects, some examples address larvae, while others deal with adults. The common response from these species may represent general trend of a broad of spectrum of organisms.

Endocides may primarily accumulate in glands including trichomes in some species. Any agents (e.g., non-toxic surfactants) or practices that can release the endocidal chemicals from glands can induce or enhance the endocidal effects in the producing species.

### A Propagule or Individual Organism Contains Adequate Endocides to Kill Itself When Externally Applied

Our experiments showed that applications of endocides can kill or eliminate the propagules, individual, or species of the parent organism, as well as their closely-related species. The applied dosages of 5% extracts in plant soaking treatments are usually lower or close to the amount can be extracted from the plant matter to be treated. For example, all of the 30 *P. koraiensis* seeds lost viability after they were treated by 0.75 g *P. koraiensis* seed extracts for five weeks while these 30 seeds can produce 1.21 g extracts. 80% of the 60 *T. sebifera* seeds lost viability after soaking in 1.5 g extracts of *T. sebifera* seeds for six weeks while these seeds can produce 2.11 g extracts. All of the 30 *A. hypogaea* seeds treated by 3 g *A. hypogaea* seed extracts for a week lost viability while these seeds can produce 12.47 g extracts. 54.3% of the 30 *Q. shumardii* acorns lost viability after a soaking treatment with 7.5 g of the *Q. shumardii* acorn extracts for 48 h while those acorns can produce 18.96 g extracts. Also, four of the six stems of *O. ficus-indica* were killed after soaking in 5 g *O. ficus-indica* extracts for 12 days while the six *O. ficus-indica* stems can produce 4.93 g extracts. Similar results were found in insect treatments. A larva of *T. molitor* or *Z. morio* can be killed by its own extracts at a dosage less than the chemical contents a worm can produce. Because only a percentage of the actual chemical constituents in an organism can be extracted by a solvent due to limitations in the extraction techniques, the yield of extracts from an organism or its part is always much lower than its actual chemical contents. Further, only a small portion of the extracts in a soaking solution became available to the plant matter to be treated. We also found that longer periods of endocides application or higher dosage of endocides have more effective inhibition and elimination results. For example, approximately 99.8% of *B. oleracea* seeds soaked in 5% *B. oleracea* seed extracts for two weeks lost viability in comparison with 32.2% of *B. oleracea* seeds soaked in the same extracts for 48 h. We thus concluded that a propagule or individual organism contains more than enough endocides to eliminate itself.

### Selective Toxicity of Endocides

In contrast to the existing studies^24^,^25^,^26^, we found that some endogenous metabolites are more toxic to the parent species than non-closely-related species. The extracts or isolates can inhibit or kill the producing species but have limited or no impacts on the growth of other species at the same dosage. Extracts of *C. vulgaris* of Charophyta eliminated all three green algae species (both Chlorophyta and Charophyta) in our tests. At the effective concentrations to the producing species, *C. vulgaris* extracts had no inhibitive activity on flowering plants *N. cristata, Utricularia macrorhiza*, and *Isolepis prolifera* (Supplementary Information 2.1).

This selectivity is especially true in species with glands that serve as the primary accumulation sites of metabolites. Extracts of *S. molesta* can kill *S. molesta, S. minima*, or *Azolla caroliniana* of Salvinales but had no impacts on any other tested species (Supplementary Information 2.3.9). In the intact plant experiment, 10 μL extracts of glyphosate-resistant *Amaranthus palmeri* can kill the tissues of glyphosate-resistant *A. palmeri* but had no impacts on *Q. texana* (Supplementary Fig. S28). In the initial field tests, the extracts of *A. palmeri* at the fatal dosage to the producer had no significant damage on the associated species *Ipomoea pandurata* (Supplementary Fig. S27).

In the field treatments, *T. sebifera* leaf and stem extracts killed *T. sebifera* seedlings, but had no impacts on the emergence and growth of *L. sinense* or *Q. texana* seedlings. Extracts of *C. capitatus* var. *lindheimeri*, a native weed species of Eurphorbiaceae controlled the producer totally (Supplementary Fig. S33) but had no inhibition on *Acalypha rhomboidea* of the same family or the monocots *Cynodon dactylon* and *Curcuma longa* during the same treatment.

*Schinus terebinthifolius* seedlings were damaged by the 10% extracts of *S. terebinthifolius* fruits after the first treatment (Supplementary Fig. S35). Four weeks later, five of the six *S. terebinthifolius* seedlings treated by the extracts alone were dead with one injured. The seedlings of *Toxicodendron radicans* of the same family and *L. styraciflua* or *Q. shumardii* were not impacted by the extracts. Furthermore, *S. terebinthifolius* seedlings were not inhibited by either surfactants or the 10% extracts of *T. sebifera* fruits.

The selectivity was also observed in insects. A larva of *T. molitor* and *Z. morio* contained enough chemicals to kill itself when externally applied (Supplementary Information 2.34). Although both are in the Tenebrionidae family, each is more susceptible to its own extracts over others at the same dosage. Further, the viability of *S. invicta* of order Hymenoptera was not impacted by the extracts of *T. molitor* of order Coleoptera even at the dosage of 1.34 times (on the basis of body weight) higher than the effective concentrations to *T. molitor*.

Our data showed that less evolved plants seems more sensitive to higher plant and endocidal selectivity, increasing from the earliest green algae through bryophytes and ferns to complex seed plants. The endocidal activity of green alga *C. vulgaris* was active in both Chlorophyta and Charophyta. *Salvinia molesta* extracts were active in Salvinales but did not show activities against seed plants or the ferns in Polypodiales of the same class Polypodiosida. However, extracts of flowering plants *S. terebinthifolius* or *C. capitatus* var. *lindheimeri* did not show activity against other genera in its family at effective concentrations to the producer.

We observed that exogenous autotoxicity occurs when a pure population of a single aquatic plant species reaches a certain size in a controlled condition. This does not happen when the species is cultured with its non-closely related species. In comparison with the pure culture of each species, the living biomass of *S. molesta, P. stratiotes, E. crassipes,* and *Myriophyllum aquaticum* in the mixed culture increased by 76.2, 127.6, 133.3, and 664%, respectively, during the three months of observation (Supplementary Fig. S53). *Salvinia minima* plants experienced autotoxicity after eight weeks of mono-culture, but its growth was improved by addition of *N. cristata* and *P. stratiotes*, at which point, all species grew well. These added species are usually not impacted, or are less impacted by the endocides of the producing species. In another experiment, *S. minima* poisoned by endocides were transferred into another container of new tap water with *N. cristata, L. minuta*, and *W. brasillensis*. All four species grew well in the new environment.

The antidotal action on autotoxicity from other non-closely related species does not occur in aquatic species only. Soil sickness or autotoxicity in a land crop field can be alleviated or avoided by proper rotation of different species^42^, and mixed-species plantations over pure plantations in sustainable forest management^43^ may help largely because of the antidotal action of other species. The antidotal action from other species is mainly because these species absorb or adhere some endocides and thus reduce the risk of autotoxicity in the producing species.

### Endocide Concept

Endocide is a biocide derived from an endogenous toxic metabolite that does not cause apparent poison in normal growth of the producing species but will poison or inhibit the parent when induced into production. It can selectively eliminate the parent or other individuals of the same species, as well as its relatives when externally applied. The endocide concept is different from allelopathy. Reported allelopathic and autotoxic examples usually describe chemical interactions between individuals of different species (occasionally for intraspecific), and the chemicals from an individual are more toxic to neighbouring individuals over the producer. However, endocides involve chemical actions within a species or an individual and may not involve other species or individuals. A species produces endocides which are more toxic to the producer than others. Organisms must use endocidal metabolites to negatively regulate themselves in order to have correct growth, development, or reproduction during the normal process.

The endocide theory can be applied broadly in all organisms. Yeasts and *Paramecium* in classic Gause’s experiments^44^ are endocidal regulation examples of kingdoms of Fungi and Protista, respectively. In the Kingdom of Monera, it was found that the accumulation of toxic metabolic waste, lack of nutrients, and unfavorable environmental conditions cause bacterial death in the stationary and death phases of the bacterial life cycle^45^.

Although the endocidal compounds of most species have not been determined, identified isolates responsible for endocidal activities to date are SMs (e.g., phenolics and apocarotenoids of *S. molesta*, coumarin of *T. sebifera*, alkaloids of *Camptotheca*, alkaloids of *S. invicta*, and formic acid of ants). Endocidal compounds are primarily SMs. Furthermore, we found that the majority of chemical components of the extracts in these species have no endocidal activities. We thus speculate that only some of the endogenous SMs play a role of endocidal regulation in the growth, development, and reproduction of an organism. A low level of endocides will allow the producing organism to have normal physiological processes while a higher level of endocides may result in abnormal processes or dormancy and even death.

An organism may usually have more than one or one class of endocidal compounds. Closely-related species may produce the same or similar endocidal compounds, but non-closely related species may produce a different class of endocidal compounds. For example, enhanced biosynthesis of CPTs by pruning or through external applications of CPTs induced the same or similar autotoxicity; thus, the alkaloids CPTs are major endocidal compounds in *Camptotheca*^18^. Pruning and soaking of acorns with acorn extracts in *Quercus* induced similar results as observed in *Camptotheca*, but it is likely the phenolics are the major endocidal compounds in *Quercus*. In some cases, it is probably difficult to determine any single potent endocidal compound from the active extracts and interactions of multiple compounds in the same or different classes that may be responsible for endocidal activities. Toxicity of an endocide on a species may primarily depend on the occurrence of the same or similar endocidal agent in this species.

Endocides are conceptually different from autotoxins which refer to chemicals in the plant exudates or decomposed residues responsible for exogenous autotoxicity. Autotoxins must be released from the producer into the environment and then the sufficient accumulation impacts conspecific individuals via the changed environment (e.g., changes of microorganisms, parasitic organisms, other biotic factors, and physical factors and chemical accumulation). Although they may have direct inhibitory effects on the germination and growth of the producing species, autotoxins are often found to be toxic or even more toxic (allelopathic) to other species. The reports of autotoxins are mainly in some plants and microorganisms. An endocide plays a regulatory role in the producing species of all organisms and can cause endogenous autotoxicity without involvement of any other individuals or environment. When externally applied or massively accumulated in the environment, an endocide can cause exogenous autotoxicity. Unlike the reported autotoxins, however, endocides usually have selective toxicity on producers and their closely-related species.

**Figure 10:**
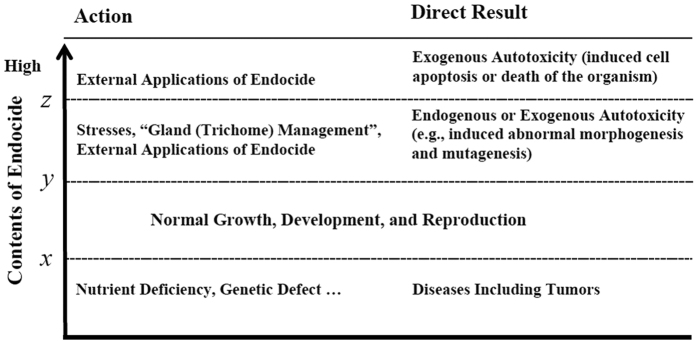
Possible roles of endocides of various concentrations on growth, development, and reproduction of the producing organism.

An organism has normal growth, development, and reproduction at certain levels of endocides (between *x* and *y*, usually relatively low concentrations) (Fig. 10). Due to nutrient deficiency or a gene missing, the endocide in an organism may be below the “*x* level” for normal growth, development, and reproduction. Under this condition, the organism may also develop diseases including tumors. In such a scenario, a supply of endocide would improve the condition to restore its healthy state. Under stress, however, an organism may produce more endocidal chemicals than the upper limit, or “*y* level” for normal growth, development, and reproduction, thus experiencing autotoxicity and abnormal growth, development, and reproduction (e.g., induced tumors by these autotoxic chemicals). To recover its normal (healthy) condition, the organism must decrease or stop the production or supply of endocides.

The question of why an organism increases the biosynthesis of its endocides under stress cannot be answered by current ecological or defense theories. Based on our data, we assert that self-regulation is the primary role of endocides in the producing organism. Endocidal regulation occurs prior to competition in a pure population. Mutations induced by the enhanced production of endocides under stress are a driving force of new species evolution. Endocide-induced mutations may be directional in some plants, e.g., shrubbiness/dwarfism, pleiocotyly, abnormal leaf morphogenesis, disturbed phyllotaxis, fasciated stems, and variegation. An induced mutation may not be the choice of the producing organism, and a muted form of the producing species is not necessarily more adapted to the environment or more defensive against its competitors or enemies than its original form. The fate of an induced mutation is determined by natural selection although endocidal regulation is a chemical process independent of other natural processes in evolution. The detailed evolutionary implications and mechanisms of endocides in the producing organisms will be addressed in the following paper.

Mutations induced by enhancing the biosynthesis of or by directly applying endocides provide a novel approach to developing new varieties of the producing or related species. The development of cultivars ‘Hicksii’, ‘Katie’, and ‘CT168’ with high levels of endocidal CPTs from the tree species *C. lowreyana* are good examples^18^,^29^,^30^. The induced chemical biosynthesis and derivatization by endocides can be used in the management of pharmaceutical crops and in the production of novel molecules. When endocides in an organism reach higher levels (> the level *z*), the organism cannot avoid loss of cells or organs, and it may die. Endocides are usually more selective to the producing organism over non-related species because it uses its endocides for self-regulation. External application of endocides can selectively inhibit and prevent the growth and reproduction of the producing species or its tissues. Thus, the endocide concept provides a novel environmentally friendly approach for controlling invasive or unwanted fast-growing species or tissues.

## Methods

### Morphological Mutations Induced by Prolonged Soaking of Fruits (Seeds) in Water

The species used in the soaking experiments are summarized in the Supplementary Table S1.

#### Camptotheca lowreyana

Fruits of *C. lowreyana* were collected from a single tree. Randomly selected fruits were divided into two groups with 900 fruits each: (1) not treated and stored at 20 °C for nine weeks and (2) soaked in water in nine containers separately (100 fruits in a plastic container with 100 mL of water) at 20 °C for nine weeks. The fruits were then sowed in pots with soil in a greenhouse (30 °C during the day time and 20 °C at night). Seeds received daily water for germination. Germination and seedlings with abnormal true leaves or stems were documented weekly. In the next two years, the seedlings with mutated leaves or stems were propagated from hardwood stem by cutting with rooting hormones. A mist system in the greenhouse was used. CPT contents of the three 3-year old plants of each developed cultivar were analyzed by the established method with ASE 200 Accelerated Solvent Extractor and Agilent/HP 1100 HPLC^35^.

#### Quercus spp

Acorns of *Q. shumardii, Q. texana*, and *Q. michauxii* were collected from Nacogdoches, Texas, USA. Every species had 30 sound acorns in each of the following two treatments with three replications per treatment: controls (no soaking treatment) and soaking in an amount of water just adequate water to cover all acorns for 48 h at 20 °C and then storage in 4 °C for four weeks. The acorns were sowed in the pots with Miracle Grow Potting Mix in the greenhouse. The seedling number with multiple stems (2–3 stems) derived directly from the same radicle in the germinated seedlings was surveyed.

### Morphological Mutations Induced by External Applications of Extracts

#### Triadica sebifera

Preparations of Leaf and Stem Extracts: The leaves and stems were collected from Nacogdoches, Texas, USA and were dried in an oven at 65 °C for 48 h. 11 kg of dried leaves and stems were ground to coarse powders and were extracted two times for 48 h with 95% EtOH (40 L and 24 L, respectively) at 20 °C. Extracts were evaporated under reduced pressure. 410 g EtOH extracts were obtained and then stored in 4 °C. Preparations of Seed Extracts: The seeds were collected from the same source as the leaves and stems and were dried in an oven at 65 °C for 48 h. 110 g dried seeds were ground to coarse powders and extracted two times for 48 h with 95% EtOH (500 mL and 400 mL, respectively) at 20 °C. The extracts were evaporated under reduced pressure. 6.3 g extracts were obtained and then stored in 4 °C. Extracts Yield of Experimental Seeds: Based on the seed weight and extraction rate (5.73%, in dry weight) of the extraction method used, it is estimated that the plant matter for each soaking treatment (60 seeds) could produce 0.41 g EtOH extracts. To further reveal the actual amount of the extracts contained in the plant matter, a more effective extraction of 60 seeds (7.1 g) was performed using a ASE 2000 Accelerated Solvent Extractor (60 °C, 1500 psi, 30 min static time, 100% volume flush, 120 s purge, and 2 cycles). According to this extraction rate, 60 seeds contain at least 2.11 g EtOH extracts. Soaking Treatments: Both leaf and stem extracts and seed extracts were prepared as an experimental solution with nanopure water at 5% concentration each. A total 900 seeds were prepared for the five following treatrments and each treatment included 60 seeds in Petri dishes at 20 °C with three replications per treatment: (1) controls: no soaking treatment, (2) water-24 h: soaked in 30 mL of nanopure water for 24 h, (3) water-6 weeks: soaked in 30 mL of nanopure water for six weeks, (4) 5% stem extracts-6 weeks: soaked in a 30 mL of 5% solution of leaf and stem extracts (1.5 g extracts) for six weeks, and (5) 5% seed extracts-6 weeks: soaked in a 30 mL of 5% solution of seed extracts (1.5 g extracts) for six weeks. Germination: Seeds were sowed in pots in the greenhouse. The number of germinated individuals and cotyledon number were recorded once every week throughout the experimental period. The germination rate and pleiocotyly rate were determined for each replicate.

#### Quercus shumardii

Preparation of EtOH Extracts: Acorns were dried in an oven at 65 °C for 48 h. 1 kg of dried acorns were ground to coarse powders and extracted two times for 48 h with 95% EtOH at 20 °C. Extracts were evaporated under reduced pressure. 50 g EtOH extracts were obtained. The extracts were prepared as experimental solutions with nanopure water at 0.5 and 5% concentration, respectively. Extracts Yield of Experimental Acorns: Based on the acorn weight and extraction rate (5.09%, in dry weight) of the extraction method, it is estimated that the plant matter for each soaking treatment (30 acorns) could produce 12.22 g EtOH extracts. To further reveal the actual amount of the extracts contained in the plant matter, a more effective extraction of small plant sample (24 g) was performed using ASE 2000 method. According to this extraction rate, 30 acorns contain at least 18.96 g EtOH extracts. Soaking Treatments: A total 270 acorns were prepared for the five following treatrments and each treatment included 30 acorns in a plastic container (14 × 15 cm, 0.68 L) at 20 °C with three replications per treatment: (1) controls (no soaking treatment), (2) soaking in a 0.5% solution of acorn EtOH extracts (0.75 g) for 48 h, and (3) soaking in a 5% solution acorn EtOH extracts (7.5 g) for 48 h. Germination Tests: The acorns were then sowed in the pots with Miracle Grow Potting Mix soil in the greenhouse. Survey of seedlings was conducted three months later. HPLC Analysis of Normal and Extracts-induced Abnormal Leaves of Seedlings: One leaf was randomly collected from each of the two two-month-old normal seedlings, and one normal leaf and one bi-lobed leaf were collected from each of the two abnormal seedlings induced by 0.5% acorn extracts. The samples of acorns and leaves were dried in an oven at 65 °C for 48 h. The dried samples were weighed and ground and extracted by using the ASE 2000 method. Each leaf samples (0.2 g) and acorn (10 g) were loaded in 22 mL cells and a 33 mL cell. 95% EtOH was used as the solvent. The extracts were evaporated under reduced pressure, transferred into a 10 mL volumetric flask, then diluted volume with 95% EtOH and mixed as experimental solutions. The HPLC chromatographs of oak leaves and acorn extracts were established by Agilent 1100 HPLC system coupled to an Agilent 1100 diode array detector, and an Eclipse XDB-C18 column (4.6 × 150 mm, 3.5 μM) at a flow rate of 0.6 mL/min. A gradient elution was performed by using nanopure water (A) and CH_3_CN (B) as mobile phases. Elution was performed according to the following conditions: 2% B at time 0, linear increase to 98% B in 22 min, and hold 98% B for 8 min. The injection volumes were equivalent to 0.34 mg plant material for all analyses. The column temperature was maintained at 23 °C. The HPLC chromatogram was standardized on retention times and peak intensities of the peaks observed at a wavelength of 254 nm.

#### Arachis hypogaea

Extracts Preparation: 500 g dried pod shell and 1,500 g dried seeds (nuts) without shell were ground separately to coarse powders and extracted two times for 48 h with 95% EtOH (4.5 L and 2.5 L each, respectively) at 20 °C. Extracts were evaporated under reduced pressure, and 23.4 g shell extracts and 31.2 g seed extracts were obtained. 10 g each of the extracts were dissolved and suspended in water and prepared separately as 200 mL experimental solution at the concentration of 5%. Extracts Yield of Experimental Plant Matter: Based on the plant matter weight and extraction rate (4.68% for shell and 2.08% for seeds, in dry weight) of the above extraction method, it is estimated that the plant matter for each soaking treatment (30 fruits) could produce 0.52 g shell EtOH extracts and 0.73 g seed EtOH extracts. To further reveal the actual amount of the EtOH extracts contained in the plant matter, a more effective extraction of small plant samples (8 g shells and 7.6 g seeds) was performed using above ASE 2000 method. According to this extraction rate, 30 fruits contain at least 0.83 g shell EtOH extracts and 12.47 g seed EtOH extracts. Soaking Treatments: The treatment experiments were conducted at 20 °C. 360 seeds in total were prepared and 30 seeds in a plastic container were subjected to one of the four treatments for one week with three replications per treatment: (1) controls: without any treatment and the seeds were directly sowed in the pots in the greenhouse; (2) soaked in 60 mL of nanopure water; (3) soaked in a 60 mL of 5% solution of shell extracts (3 g); and (4) soaked in a 60 mL of 5% solution of seed extracts (3 g). Germination: All experimental seeds were sowed in pots with Miracle Grow Potting Mix soil in the greenhouse. The morphological variations of each seedling were recorded weekly throughout the experimental period of three months.

#### Opuntia ficus-indica

Extracts Preparation: The fleshy oval stems (300 g in dry weight) were ground to a coarse powder and extracted two times for 48 h each with 95% EtOH (1.2 L each time) at 20 °C. The combined extracts were concentrated to give 16.6 g under reduced pressure. The 5 g extracts were dissolved in nanopure water and prepared as 100 mL experimental solution at the concentration of 5% extracts. Extracts Yield of Experimental Plant Matter: Based on the plant matter weight and extraction rate (5.54%, in dry weight) of the above extraction method, it is estimated that the plant matter for each soaking treatment (six stems) could produce 4.93 g EtOH extracts. Soaking Experiment: 12 pieces of stems (15–17 cm) were prepared and subjected to two treatments. Six stems were cultivated in 100 mL of nanopure water to serve as the controls and six stems were cultivated in 100 mL of 5% extracts (5 g) for 12 days at 20 °C. Growth and Propagation Tests: Each experimental stem was placed in one-gallon pots with Miracle Grow Potting Mix soil in the greenhouse. The living status of individuals was recorded once every week throughout the experimental period.

#### Brassica oleracea

Preparations of Seed Extracts: The seeds were dried in an oven at 65 °C for 48 h. 120 g dried seeds were ground to coarse powders and were extracted two times for 48 h each with 95% EtOH (400 mL each time) at 20 °C. Extracts were evaporated under reduced pressure. 4 g EtOH extracts were obtained and then stored in 4 °C. 1.5 g of seed extracts were dissolved in nanopure water and prepared as 30 mL experimental solution at the concentration of 5%. Extracts Yield of Experimental Plant Matter: Based on the plant matter weight and extraction rate (3.34%, in dry weight) of the above extraction method, it is estimated that the plant matter for each soaking treatment (300 seeds) could produce 0.03 g EtOH extracts. To further reveal the actual amount of the extracts contained in the plant matter, a more effective extraction of small plant sample (0.88 g) was performed using the ASE 2000 method. According to this extraction rate, 300 seeds produced 0.1 g EtOH extracts. Soaking Treatments and Germination Tests: 1,800 sound seeds were selected and 300 seeds in a Petri dish were subjected to one of the following soaking treatments for 48 h at 20 °C with three replications per treatment: a 10 mL of nanopure water (to serve as the controls) and a 10 mL of 5% solution of seed extracts (0.5 g). Seeds were sowed in germination box with soil in the greenhouse. The number of germinated individuals and cotyledon number, leaf morphology, and stem number were recorded once every week throughout the 4-week experimental period.

### Morphological Mutations Induced by Decapitation Pruning

#### Camptotheca lowreyana ‘Katie’

During the cultivar development of ‘Katie’, repeated pruning of the original mutated seedling was conducted to propagate cuttings. The repeated pruning directly induced the mutation in a stem of the original plant. The mutated stem had reduced internodes and smaller leaves. The mutated stem was propagated by a cutting without the use of hormones using a mist system in the greenhouse. In the next three years, repeated propagation by cuttings was made from the rooted plants with rooting hormones or without hormones. CPT contents of the three 3-year old plants of each developed cultivar were analyzed by the established method^39^.

#### Other Woody Species

10 woody species with different habits and leaf types were selected for pruning experiment (Supplementary Table S3). Six mature plants were selected from each species in the field in Nacogdoches, Texas, USA. Three plants of each species were served as the controls receiving no treatment. All main stems of the remaining three plants from each species were removed from above ground at approximately 15–30 cm in December. The first five newly developed leaves in each pruned plant were surveyed and photographed in March of the next year.

### Inhibition and Elimination of the Producing Species by External Applications of Endocides

#### Inhibition and Elimination of S. molesta by Salviniside II, an Isolate from water Extracts of Its Dried Matter

Isolation: Continuous fractionation was based on the combined MeOH/CH_2_Cl_2_ (1:1, v/v) and 100% MeOH fractions of *S. molesta* water extracts which showed potent activity to inhibit growth of *S. molesta* in Supplementary Information 2.3.4. The combined fractions were loaded on a pre-equilibrated open ODS column (60 × 600 mm). The ODS column was eluted successively with 30%, 55%, 75%, and 100% MeOH to yield four fractions F1, F2, F3, and F4, respectively. F3 (75% MeOH elute) was concentrated and separated by a preparative HPLC (MeOH/H_2_O, 35:65, 254 nm) to yield four compounds including active salviniside II^46^ and C3 and inactive C1 and C2, active fraction F32, and inactive fraction F31 (Supplementary Fig. S9). Foliar Sprays: The total 27 healthy and untreated living plants of *S. molesta* (in secondary growth stage, approximately 10 g in fresh weight each) were cultured and tested in plastic containers (14 × 15 cm, 0.68 L) at 20 °C. The treatments were as follows. (1) Controls: nine plants with three in each container were sprayed with 50 mL of water per container; (2) 0.01% salviniside II treatment: nine plants with three in each container were sprayed with 50 mL of 0.01% salviniside II dissolved in water per container; and (3) 0.1% salviniside II treatment: nine plants with three in each container were sprayed with 50 mL of 0.1% salviniside II dissolved in water per container. Plant growth and survival status were documented and photographed weekly after the treatments. By the end of two weeks, new growth biomass of plants in each treatment was measured. The biomass of new growth was a primary factor in measuring the inhibition of each treatment on the target plant. The data were analyzed by SPSS 13.0 for Windows (SPSS, Inc., Chicago, IL). ANOVA with Duncan post-hoc analysis was used to compare the means of the control and salviniside II treatment groups.

#### Phytotoxicity of (+)-3-Hydroxy-β-ionone Isolated from the Dried Matter of S. molesta on S. molesta

Compounds Preparation: Compounds (+)-3-hydroxy-*β*-ionone, (3R, 6R, 7E)-3-hydroxy-4,7-megastigmadien-9-one, annuionone D, and dehydrovomifoliol were isolated from a previous investigation^46^. Bioassays: Each of the four isolated compounds was prepared as 50 μL experimental solution with nanopure water at the 1% concentration. A total of 24 healthy and untreated living plants of *S. molesta* (in tertiary stage, approximately 5 g in fresh weight each) were cultured and tested in eight plastic containers (14 × 15 cm, 0.68 L) with three plants in each container in the greenhouse (30 °C during the day time and 20 °C at night). The three plants in the first container served as the controls without any treatment, three plants in the second container were treated with 1% DMSO, three plants in the third container were treated with 5% DMSO, three plants in the fourth container were treated with 0.5% Dyne-Amic, and the plants in each of the other four containers were treated with each of the four testing isolates, respectively. For each of the three plant in the 0.5% Dyne-Amic treatment, 8 μL of 0.5% Dyne-Amic was applied on the lower surface of each of the two mature leaf blades close to the terminal bud by pipet and 8 μL of 0.5% Dyne-Amic was applied on the upper surface (with trichomes) of each of the two other mature leaf blades close to the terminal bud by pipet. For all three plants in each of the DMSO or testing isolates treatment containers, 8 μL of each experimental solution was applied on the lower surface of each of the two mature leaf blades close to the terminal bud by pipet. The leaf surfaces of the experimental plants were checked daily by a ×60 portable microscope linked to an iPhone.

#### Phytotoxicity of N. cristata EtOH Extracts

Extracts Preparation: The whole plants were dried in an oven at 65 °C for 48 h. The oven-dried plant matter (3,600 g) was ground to a coarse powder and extracted with 95% EtOH at 20 °C twice (each with 20 L and 12 L, respectively) for 48 h each time. The combined extracts were concentrated under reduced pressure to yield 420 g final extracts. 100 g extracts were fractionated by Si-gel column with a gradient of n-hexane/acetone (8:1 to 1:3) to get four fractions: A (12.6 g), B (18.4 g), C (16.9 g), and D (22.3 g). Fraction A of the EtOH extracts was prepared in 1% experimental solution with nanopure water. Bioassays: 10 μL of 1% extracts were applied by pipet on the three locations of per upper leaf surface of the intact *N. cristata* plants. The treated leaves were evaluated and photographed for two weeks.

#### Phytotoxicity of Fractions and 5,6,7,8-Tetramethoxycoumarin Isolated from Leaves and Stems of T. sebifera on T. sebifera Seedlings

Extraction and Isolation: The leaves and stems were dried in an oven at 65 °C for 48 h. 11 kg of dried leaves and stems were ground to coarse powders and were extracted two times for 48 h with 95% EtOH (40 L and 24 L, respectively) at 20 °C. Extracts were evaporated under reduced pressure. 410 g EtOH extracts were obtained. The extracts were suspended in MeOH-H_2_O (1 L, 1:1, v/v), and partitioned successively with hexane and EtOAc. Three fractions were obtained, named hexane fraction (97.8 g), EtOAc fraction (31.9 g), and H_2_O fraction (220 g). 0.4 g hexane fraction was further separated by HPLC (Zorbax SB-C18 column, CH_3_CN/H_2_O:45/55 and 98/2, detection 210 nm) to give five subfractions: F45-1 (12.5 mg), F45-2 (3.8 mg), F45-3 (4.7 mg), F45-4 (8.2 mg), and F100 (67 mg). 5,6,7,8-Tetramethoxycoumarin was purified from F45-3 by HPLC (Zorbax SB-C18 column, CH_3_CN/H_2_O:45/55, detection 280 nm). NMR experiments were performed on a JEOL ECS-400. NMR data were reported as δ (ppm) values and referenced to the solvent used. Bioassays: The experimental plants were four-month-old *T. sebifera* seedlings grown in pots with Miracle Grow Potting Mix soil in the greenhouse with about 30 seedlings on each pot. Each of the fractions, subfractions, and 5,6,7,8-tetramethoxycoumarin was prepared as 50 μL experimental solution with nanopure water at 5% concentration each, respectively. For each testing sample, 10 μL of each experimental solution was applied by pipet on the upper surface of each of the three randomly selected mature leaves and the lower surface of each of three randomly selected mature leaves, respectively. On each seedling, the untreated leaves served as the controls. For active hexane fraction, foliar spray of 0.5, 1, 2.5, and 5% concentrations were applied with a dosage of 20 mL on 15 seedlings in a pot with a total 30 plants in total. The remaining untreated 15 seedlings in each pot served as the controls. The foliar treatments were replicated three times. The same foliar applications of the 5% hexane fraction were also applied on at least three one-year-old seedlings of the following species at the dosage of 10 mL per plant: *P. taeda, Vitis rotundifolia, Myrica heterophylla, Rhus aromatica, Dichanthelium boscii*, and *Desmodium* sp. The leaf surfaces of the experimental plants were checked daily for two weeks by a ×60 portable microscope linked to an iPhone.

## Additional Information

**How to cite this article**: Li, S. *et al*. Endocidal Regulation of Secondary Metabolites in the Producing Organisms. *Sci. Rep.* **6**, 29315; doi: 10.1038/srep29315 (2016).

## Supplementary Material

Supplementary Information

## Figures and Tables

**Table 1:** Major diagnostic characters of ‘Hicksii’ from three species of *Camptotheca*.

Major Diagnostic Characters	*C. acuminata* Decaisne	*C. yunnanensis* Dode	*C. lowreyana* Li	*C. lowreyana* Li ‘Hicksii’
Leaf Shape	oval/ovaloblong	elliptic	cordate/ovate	cordate
Fruit Color (dry) (RHS Color Chart)	red brown or greyed-orange (167D)	gray or greyed-orange (164 B)	gray-brown or greyed-orange (164 C)	brown (200D)
Fruit Length (mean ± s.d., mm)	22.23 ± 2.90a	20.63 ± 2.03a	29.73 ± 3.07b	14.82 ± 4.35c
Fruit Disc Thickness	thick	thin	thin	thin
Fruit Surface (dry)	rugose	smooth and lucid	smooth and lucid	smooth and lucid
Cotyledon Length (mean ± s.d., mm)	36.21 ± 5.81a	26.92 ± 3.29b	34.29 ± 4.93a	22.36 ± 5.07c

Significant differences between means in the same row are indicated by different letters (*P* < 0.05 by one-way ANOVA).
