# Supplementary material for: Endocidal Regulation of Secondary Metabolites in the Producing Organisms
Source: Sci Rep. 2016 Jul 8;6:29315. doi: 10.1038/srep29315 (PMC4937345; doi:10.1038/srep29315)
Supplement: Supplementary Information [file srep29315-s1.pdf]

Supplementary Materials for

Endocidal Regulation of Secondary Metabolites in the Producing Organisms

Shiyou Li*, Ping Wang, Wei Yuan, Zushang Su, and Steven H. Bullard

National Center for Pharmaceutical Crops, Arthur Temple College of Forestry and Agriculture
Stephen F. Austin State University, Nacogdoches, TX 75962, USA
* Corresponding author: Tel.: +1 936 468 2071; fax: +1 936-468-2058. E-mail: lis@sfasu.edu

This PDF file includes:

Materials, Methods, and Results
Supplementary Text
Figs 1to 53
Tables 1 to 7

1. Induced Mutations by Enhanced Biosynthesis or Availability of Endocides

1.1. Abnormal Morphogenesis Induced by Unconventional Prolonged Soaking of Fruits or Seeds in Water

1.1.1. Observations of *Camptotheca* Decaisne Seedlings in Greenhouse and Field

General Experimental Procedure: In December 2014, fruits were collected from a single mature tree from happytree (*Camptotheca acuminata* Decaisne) and *C. lowreyana* 'Hicksii', respectively. 100 fruits of each taxon were sowed in the pots in greenhouse (30°C during the day time and 20°C at night) without soaking in water in February 2015. During the first week of May 2015, survey was conducted on the seedling number with one or more abnormal true leaves in the germinated seedlings in the greenhouse. During this time period, the similar seedling survey was also conducted in randomly selected four plots (1 × 1 m each) under each of the parent trees in the field.

Results: The results are summarized in the Supplementary Table S2 and Supplementary Fig. S1.

1.1.2. Soaking Experiments of Happytrees (*Camptotheca* Decaisne) Fruits

General Experimental Procedure: Experimental fruits of *Camptotheca acuminata* and *C. lowreyana* 'Hicksii' (see 1.1.1) were collected from a single mature tree, respectively. 30 untreated fruits of each taxon were directly sowed in the pots in greenhouse to serve as a control. 30 fruits of each taxon were soaked in Petri dish in water at room temperature (approximately at 20°C) for 24 h and 30 fruits of each taxon were soaked under the same conditions for four weeks. The soaked fruits were then sowed in the pots in greenhouse. Each of the control and soaking treatment experiments had three replications. Weekly germination surveys were conducted and the seedlings with one or more abnormal true leaves were documented. By the end of two months, the total germinated seedling number in each of the control and soaking treatments and the number of seedlings with two or more stems derived directly from the fruit or branch number above the soil surface were counted. The data were analyzed by SPSS 13.0 for Windows (SPSS, Inc., Chicago, IL). ANOVA with Duncan post-hoc analysis was used to compare the means of the control and treatment groups.

*Results***:** For both taxa, the fruits soaked in water for 24 h had much better germination rate than either those without treatment or soaked in water for four weeks (Supplementary Fig. S2). Following the fruits soaked in water for four weeks, 15.6% *C. acuminata* seedlings developed 2-

3 branches compared to no branch development from the fruits with 24 h of soaking or no soaking treatment and 38.5% seedlings of *C. lowreyana* 'Hicksii' had 2-5 branches from the main stem in comparison with no branch development in those germinated from the fruits with 24 h of soaking or no soaking treatment.

2. Inhibition of the Producing Species by External Applications of Endocides

To reveal the actual role of SMs in the producing organisms, we selected 37 common species of organisms in 26 families as the representative of different groups of green algae, ferns, gymnosperms, angiosperms, and insects for extraction and external applications of endocides (Supplementary Table S4) and 51 species of 38 families for selectivity tests (Supplementary Table S5).

2.1. Elimination, Inhibition, and Prevention of Green Algae by EtOH Extracts of Muskgrass (*Chara vulgaris* L.)

The green algae are eukaryotic organisms of the kingdom Plantae consist of divisions Chlorophyta and Charophyta. Filamentous algae of Chlorophya, often referred to as green hair algae, pond scum, or pond moss, are the most common type of noxious algae in ponds. The attached (epiphytic) or free floating filamentous algae often interfere with recreational activities and are accused of damaging the fishing industry. Cotton-like *Cladophora* Kűtz. and horse-hair-like *Pithophora* Wittrock of the family Cladophoraceae are two commonly seen genera of filamentous algae. Muskgrass or skunkweed (*Chara vulgaris* L.) is an advanced multicellular macroalgae species of the family Chraraceae of Charophyta. The species found in fresh water may have given rise to land plants because it has stem-like and leaf-like structures. The fast-growing species with mucky or garlicky odor can be undesirable in ponds and lakes. Common algaecides containing copper can kill algal cells that the copper contacts directly but must be sprayed or broadcasted over the entire area where algae are growing to provide adequate control.

General Experimental Procedures: Extracts Preparation: Experimental *Cladophora* sp., *Pithophora roettleri* (Roth) Wittrock, and *C. vulgaris* were collected from east Texas, USA. The whole plants of *C. vulgaris* were dried in an oven at 65°C for 48 h. 2.5 kg dried plant matter was ground to a coarse powder and extracted two times for 48 h each with 95% EtOH (15 L each time) at room temperature. The combined EtOH extracts were concentrated under reduced pressure and the total 31.2 g extracts were obtained. The treatment experiments were conducted

in the greenhouse (30°C during the day time and 20°C at night). Injection Experiment I: 100 g *C. vulgaris*, 10 g crested floating heart (*Nymphoides cristata* (Roxb.) Kuntze) (Menyanthaceae), and 2 g common bladderwort (*Utricularia macrorhiza* LeConte) (Lentibulariaceae) were placed in each of six plastic containers (23 × 23 cm, 2.7 L) with 1,500 mL of tap water each. 1.5 g *C. vulgaris* extracts were placed into the water of each of the three containers while three other containers received no treatment to serve as the control group. A week later, additional 1.5 g *C. vulgaris* extracts were placed into the water of each of the three treated containers. The response of each plant species was recorded daily after the first treatment for two weeks. Injection Experiment II: Initially 2 g *N. cristata*, 3 g *U. macrorhiza*, and 2 g mixture of *Cladophora* sp. and *P. roettleri* were in each of two plastic containers (14 × 15 cm, 0.68 L) with 300 mL of tap water each. 0.4 g *C. vulgaris* extracts were placed into the water of one container while another had no treatment to serve as a control. On the third and fifth day after the treatment, additional 2 g mixture of *Cladophora* sp. and *P. roettleri* was placed in each container, respectively. The response of each plant species was recorded daily after the treatment for two weeks. Spray Experiment: 10 g extracts were dissolved and suspended in nanopure H_2O and prepared as 200 mL experimental solution at the concentration of 5% *C. vulgaris* extracts. Two plots (35 × 15 cm each) of proliferating bulrush (*Isolepis prolifera* (Rottb.) R. Br.) (Cyperaceae) plants cultured in water container were selected for the experiment. The first plot served as a control without any treatment, the second was sprayed with 20 mL of 5% extracts. The plant status was documented and photographed weekly after the treatment for four weeks.

Results: In response to the the injection of 1.5 g *C. vulgaris* extracts (at approximately 0.1% concentration of *C. vulgaris* extracts in the experimental solution, v/v), most cells of all three green algal species (*C. vulgaris*, *Cladophora* sp., and *P. roettleri*) lost green color and were separated within 48 h and all were dead within a week (Supplementary Fig. S7). At the same time, neither species of flowering plants (*N. cristata* or *U. macrorhiza*) were injured or inhibited. These two flowering plant species had even no any injury after the additional 1.5 g *C. vulgaris* extracts were applied. Additional filamentous algae (*Cladophora* sp. and *P. roettleri*) added to the container treated with *C. vulgaris* extracts on the third and fifth day could not grow. After the spray treatment of 20 mL of 5% *C. vulgaris* extracts, flowering plant species *I. prolifera* had no any injury although *C. vulgaris* was significantly damaged.

2.2. Elimination and Inhibition of Seductive Entodon Moss (*Entodon seductrix* (Hedw.) Müll. Hal.) and Atrichum Moss (*Atrichum angustatum* (Bridel) Bruch & Schimper) by Their EtOH Extracts

Seductive entodon moss (*Entodon seductrix* (Hedw.) Müll. Hal.) (Entodontaceae) is a common, straggling moss found throughout eastern North America, as far north as Ontario, and extending westward from Florida to Texas, USA. Atrichum moss (*Atrichum angustatum* (Bridel) Bruch & Schimper) (Polytrichaceae) is another common small moss species in North America. These two moss species are not invasive species, but may be unwanted by some people. Because the mosses often grow on bricks, roofs, walls, decks, patios, sidewalks, lawns, or trees and shrubs and because they may be viewed as unattractive or destructive to their properties, some homeowners are interested in controlling them.

General Experimental Procedures: <u>Extracts Preparation</u>: A mixture of seductive entodon moss (*Entodon seductrix* C. Müller) (family Entodontaceae) and atrichum moss (*Atrichum angustatum* (Brid.) Bruch & Schimp.) (family Polytrichaceae) (each accounts for approximately 50% of the total biomass in dry weight) were collected from Nacogdoches, Texas, USA in August 2014. The whole plant mixture was dried in an oven at 65°C for 48 h. 180 g dried plant matter was ground to a coarse powder and extracted two times for 48 h each with 95% EtOH (1.5 L and 1.2 L, respectively) at room temperature. The combined EtOH extracts were concentrated under reduced pressure and the total 8.7 g extracts were obtained. 3 g extracts were dissolved and suspended in nanopure H_2O and prepared as 30 mL experimental solution at the concentration of 10% extracts with 1% surfactant Tergitol 15-S-9 (Sigma-Aldrich Co., St. Louis, MO, USA) (v:v). <u>Spray Experiment:</u> Three plots of mixture of both species in 30 × 45 cm each were selected for the experiment. The first plot served as a control without any treatment, the second was sprayed with 1% Tergitol 15-S-9 Surfactant in water solution, and the third one was sprayed with 10% EtOH extracts of mosses with 1% Tergitol. The plant status was documented and photographed weekly after the treatment for four weeks.

Results: The plants in both control and 1% surfactant Tergitol treatment plots had no significant changes during the four weeks of experiments. Both *E. seductrix* and *A. angustatum* responded to 10% EtOH extracts of mosses with approximately 1% Tergitol on the second day after the treatments. Both moss species were dead within the four weeks (Supplementary Fig. S8).

2.3. Elimination, Inhibition, and Prevention of Giant Salvinia (*Salvinia molesta* D. S. Mitchell) by Its Matter, Extracts, and Isolates

Giant salvinia (*Salvinia molesta* D. S. Mitchell) (Salviniaceae), is native to Brazil, but it has invaded lake and river systems in warm climates in the world since 1939[1]. At present, *S. molesta* is one of the most widespread and environmentally, economically and socially destructive invasive plant species. Biological, mechanical, and herbicidal control of invasive *S. molesta* is very expensive but has not been successful. Recently, we have isolated 55 compounds from *S. molesta* with several abietane diterpenes having *in vitro* cytotoxicity again various human cancer cells[1]. To isolate more bioactive compounds, we grew *S. molesta* in greenhouse containers. We observed "suicide" phenomenon of *S. molesta* after months-long culture in containers with water. A series of experiments ruled out other possibilities and found that dry plant matter of *S. molesta* (packed in a bag, called "tea bag") placed at the container bottom can lead to elimination of all living *S. molesta* plants floating in the container. And no new or living *S. molesta* plants were found within the six months of observation thereafter. This demonstrates that *S. molesta* contains some agents that are toxic to the parent plants and that these agents can fully eliminate and inhibit the growth of *S. molesta* at higher concentrations. The "tea bag" treatment killed the *S. molesta* bottom-up by damaging the root-like submerged leaves of *S. molesta* first. Then the trichomes on the upper surfaces of the floating leaves of *S. molesta* were wilted following the damage of the submerged leaves. The wilted trichomes released autotoxic compounds and thus enhanced the endocidal function. The "bottom-up" killing style of the "tea bag" is particularly effective in a shallow and still water body infested with multi-layers of *S. molesta* mat because the dead tissues of the bottom layer of *S. molesta* killed by the "tea bag" would enhance the endocidal function to eliminate the next layer of *S. molesta*.

The endocide development process is summarized in Supplementary Fig. S9. As an example, the authors studied *S. molesta* plants. It was found that endocides derived from either dried or fresh plant matter of *S. molesta* can effectively control the invasive species in all growth stages (primary, secondary, and tertiary stages). It was found that dry plant matter of *S. molesta* can fully eliminate *S. molesta* plants when adequate dried ground matter of giant salvina is placed in the water.

Salvinia species are rootless, free-floating aquatic ferns and they are well-known for their extremely water-repellent floating leaves. On the upper surfaces of the floating leaves of *S.*

molesta, four multicellular glandular trichomes have their apical cells connected to form egg-beater structures. The arrows point to the egg-beater structures of trichomes which were removed by blade. The root-like submerged leaves are highly branched with multicellular trichomes and on which sporocarps developed. Our experiments found that the active agents of *S. molesta* are primarily accumulated in trichomes which are usually intact, and the plant can avoid poison by these toxins. However, *Salvinia* spp. are well-known for their water-repellent floating leaves because of the leaves are densely covered with peculiar trichomes. On the upper surfaces of floating leaves of *S. molesta*, four multicellular trichomes have their apical cells connected to form egg-beater structures (Supplementary Fig. S10). The root-like submergered leaves also have dense multicellular trichomes. The basal cells of the trichomes in *S. molesta* are covered with wax. Therefore, the bioactive compounds in trichomes of the living plants are not released into the water because of this water-repellent feature. The removal or damage of the apical cells (egg-beater structures) of trichomes will not only release the chemicals in storage but also allow water to access the floating leaves to soak the bioactive agents out. This invention emphasizes that breaking the trichomes (e.g., mechanical treatments such as grinding, blending, squeezing, or heavily washing plant tissues) is necessary for effective extraction of the bioactive agents from *S. molesta*.

2.3.1. Elimination and Inhibition of Giant Salvinia (*Salvinia molesta* D. S. Mitchell) by Its Dried Matter

General Experimental Procedures: Preparation of Experimental "Tea Bags": Air-dried whole plants of *S. molesta* were ground to a coarse powder and placed in nylon net bags (called "tea bags"). Treatments: Each of the 12 containers had 400 g living healthy plants of *S. molesta* in all growth stages (primary, secondary, and tertiary stages) in 25 L of tap water in the greenhouse (30°C during the day time and 20°C at night). Three containers had no treatment to serve as controls. The first treatment group had three containers and each had one bag of 100 g *S. molesta* dried matter in the water. The second group of three containers had one bag of 200 g *S. molesta* dried matter, and the last group of three containers had one bag of 400 g *S. molesta* dried matter. There was no significant difference of pH values between the treatments and control during the six months of experiments. Statistic Analysis: The data were analyzed by SPSS 13.0 for Windows (SPSS, Inc., Chicago, IL). ANOVA with Duncan post-hoc analysis was used to compare the means of the control and treatment groups.

Results: Both 200 g and 400 g treatment had significant different effects on the growth of *S. molesta* than either 100 g or no treatment (Supplementary Fig. S11). In the 400 g treatment group, more than 35% of plants were dead by the end of the first week and all plants were dead by the end of the second week and no plants survived or new growth occurred thereafter during the observation of six months (Supplementary Figs. S11 and S12). In this treatment, the root-like submerged leaves of *S. molesta* were totally damaged about a week later and then the floating leaves turned into brown and dead. In contrast to those in the normal plant growth condition, the trichomes on the upper surfaces of the floating leaves of *S. molesta* were soon wilted following the response of submerged leaves to the "tea bag" treatment. The wilted trichomes released autotoxic compounds and thus enhanced the endocidal function. It was also found that the 200 g treatment group inhibited almost 50% of the plants by the end of the four weeks of the experiment. However, no significant effect of the 100 g treatment was observed on salvinia growth.

2.3.2. Prevention of Giant Salvinia (*Salvinia molesta* D. S. Mitchell) Growth by Its Dried Matter

General Experimental Procedures: Air-dried whole plants of *S. molesta* were ground to a coarse powder and placed in nylon net bags (called "tea bags"). Each of the three containers in which all plants were dead and decomposed following the treatment of 400 g "tea bag" (see 2.3.1) was refilled with tap water to 25 L. Then 110 g healthy *S. molesta* plants (10 g in primary stage and 100 g in tertiary stage) were added to each of these three containers. Each of three containers was filled with 25 L of tap water and had the same amount of *S. molesta* plants to serve as the control. Each of these six containers was maintained at 25 L water level during the next three months and the experiments were conducted in the greenhouse (30°C during the day time and 20°C at night).

Results: In all three containers previously treated with 400 g "tea bag", the added young *S. molesta* plants in primary stage experienced initial slight growth but soon became unhealthy within two weeks (Supplementary Fig. S13) and all were dead within the next three months. In these containers, all mature *S. molesta* plants became unhealthy after two weeks of culture and root-like submerged leaves were dead. Although young leaves might develop from some terminal buds, all plants were eventually dead by the end of three months. Further, there was no

new growth of *S. molesta* was observed in containers in the next three months of experiments. In contrast, both young and mature *S. molesta* plants in the control group had significant growth and total fresh biomass increased to 246.9±54.2 g at average from 110 g during the first three months of experiments.

2.3.3. Elimination and Inhibition of Giant Salvinia (*Salvinia molesta* D. S. Mitchell) Plants by H_2O Extracts of Its Dried Matter

General Experimental Procedures: Extracts Preparation: Air-dried whole plants (350 g) were ground to a coarse powder and percolated with H_2O at room temperature to yield a 4 L aqueous solution (0.84%, g/mL). Of the aqueous solution, 119 mL was diluted to 900 mL of 0.1% and 1,000 mL of 0.01% solution (g/mL) for the following experiment. Foliar Sprays: The experiment included 45 healthy and untreated living plants of *S. molesta* (in secondary growth stage, approximately 7 g in fresh weight each). The plants were cultured and tested in plastic containers (14 × 15 cm, 0.68 L) in the laboratory at room temperature. Controls: 15 plants with five in each container (three replications) were cultured with 300 mL of tap water; 0.01% H_2O extracts treatment: 15 plants with five in each container (three replications) were sprayed with 300 mL 0.01% of the H_2O extracts of *S. molesta* dissolved H_2O; and 0.1% H_2O extract treatment: 15 plants with five in each container (three replications) were sprayed with 300 mL of 0.1% the H_2O extracts of *S. molesta* dissolved in H_2O. Plant growth and survival status were documented and photographed weekly after the treatments. By the end of the 13th week, new growth biomass of the plants in each treatment was measured. The biomass of new growth was a primary factor to measure the inhibition of each treatment on the target plant.

Results: By the end of 13 weeks of treatment, each of the 15 plants in the control group had new growth, and 12 of the total 15 plants treated with 0.01% H_2O extracts of *S. molesta* had new growth (Supplementary Fig. S14). All *S. molesta* plants treated with 0.1% H_2O extracts of *S. molesta* were dead and no new growth developed from any of the plants during the eight months of observation. The pH values of the culture solution varied from 7.4 to 7.6 and there were no significant differences among the control and spray treatments.

2.3.4. Elimination and Inhibition of Giant Salvinia (*Salvinia molesta* D. S. Mitchell) Plants by Fractions of H_2O Extracts of Its Dried Matter

General Experimental Procedures: Extracts Preparation: Air-dried whole plants of *S. molesta* (1.4 kg) were ground to a coarse powder and extracted two times for 48 h with H_2O (12 L × 2) at room temperature. The combined H_2O extracts were concentrated to give extracts (88 g) under reduced pressure. The H_2O extracts was applied on a column of silica gel (1,000 g) eluting with the mixture of MeOH/CH_2Cl_2 (1:1, v/v, 3L), 3 L of 100% MeOH, and 1L H_2O to obtain three fractions, respectively**.** The H_2O extracts and three fractions were all prepared as experimental solutions at the concentration of 0.1%. Foliar Sprays: The total 45 healthy and untreated living plants of *S. molesta* (in secondary growth stage, approximately 10 g in fresh weight each) were cultured and tested in the plastic containers (14 × 15 cm, 0.68 L) in the laboratory at room temperature. The five treatments are as follows. Controls: nine plants with three in each container (three replications) were sprayed with 50 mL of tap water per container; H_2O extract treatment: nine plants with three in each container (three replications) were sprayed with 50 mL 0.1% of the H_2O extracts of *S. molesta* dissolved in H_2O per container; H_2O fraction treatment: nine plants with three in each container (three replications) were sprayed with 50 mL of 0.1% H_2O fraction dissolved in H_2O per container; MeOH fraction treatment: nine plants with three in each container (three replications) were sprayed with 50 mL of 0.1% MeOH fraction dissolved in H_2O per container; and MeOH/CH_2Cl_2 fraction treatment: nine plants with three in each container (three replications) were sprayed with 50 mL of 0.1% of MeOH/CH_2Cl_2 (1:1) fraction dissolved in H_2O per container. Plant growth and survival status were documented and photographed weekly after the treatments. By the end of 2nd week, new growth biomass of plants in each treatment was measured. The biomass of new growth was a primary factor to measure the inhibition of each treatment on the target plant.

Results: On the 3rd day after the treatment, all plants treated with MeOH/CH_2Cl_2 or MeOH fraction were dead in comparison to 100% survival of the plants in the control group. During the same day of observation, some leaves of the plants treated with H_2O extracts or H_2O fraction started to turn into brown color. By the end of two weeks, all plants in the control group were still alive (Supplementary Fig. S15). By then, in contrast, no plants survived or new growth in both MeOH/CH_2Cl_2 and MeOH treatments, almost all plants were dead with H_2O extracts, while the biomass of living plants under the H_2O fraction treatment was less than 25% of the plants in

the control group**.** The experiment indicated that $MeOH/CH_2Cl_2$ and MeOH fractions were very active and can fully control *S. molesta* more quickly than its parent H_2O extracts and thus the following isolation of bioactive compounds was focused on these two fractions.

2.3.5. Elimination and Inhibition of Giant Salvinia (*Salvinia molesta* D. S. Mitchell) Plants by Hydraulic Extracts of Its Fresh Matter

General Experimental Procedures: <u>Extracts Preparation</u>: The total 58 kg fresh matter of *S. molesta* plants were chopped and shredded by food processor. Then the processed material was pressed by hydraulic press (Enerpac) at pressure of 27,579 kPa (4,000 psi) for 2 min to get 30 kg extract juice. To obtain the concentration of the extract juice, three 30 mL of juice samples were randomly collected and measured. Each of the three juice samples was centrifuged by accuSpin 3R Benchtop centrifuge (Fisher Scientific, PA, USA) at 3,000 rpm for 10 min. The upper liquid was first dried by SPD 2010 Integrated SpeedVac (Thermo Scientific, NC, USA) for 12 h. Then dried extracts were measured. The average result of the three test samples was used to estimate the concentration of the extract juice. The concentration of hydraulic extract juices were 2 kg fresh plant matter/L. <u>Foliar Sprays</u>: The spray treatment experiment included three plastic containers (20 gal, approximately equal to 75.7 L) with approximately 390 g (in fresh weight) of *S. molesta* living plants (in all growth stages: primary, secondary, and tertiary stages) in 50 L of tap water each in the greenhouse (30°C during the day time and 20°C at night). There were three treatments. Controls: the plants in the first container were sprayed with 150 mL of H_2O only; extract juice treatment: the plants in the second container were sprayed with 150 mL of extract juice; and extract juice and surfactants treatment: the plants in the third container were sprayed with 150 mL of extract juice with 0.375 mL of Inlet (polyalkoxylated and non-alkoxylated aliphatics and derivatives 90%, Helena Chemical Company, Collierville, TN, USA) and 0.1875 mL of Kinetin (proprietary blend of polyalkyleneoxide modified polydimethylsiloxane 99%, Helena Chemical Company, Collierville, TN, USA). The second same spay treatment was applied to each of these six contains a week later, respectively. Plant growth and survival status were documented and photographed weekly after the treatments. By the end of 6th week, the total biomass of living plants in each treatment was measured. To investigate the survival rate of *S. molesta* after hydraulic extraction, the 28 kg (in fresh weight) residual plant material of *S. molesta* expressed by hydraulic press was cultured in five containers with 50 L water each for six weeks.

Results: The extraction rate of hydraulic extracts from the fresh matter of *S. molesta* in this experiment was approximately 51.7%. Based on the three test samples, the estimated concentration of the experimental extract juice was 0.28% (in fresh weight). The spray experiment showed that the hydraulic extract juice, particularly with surfactants effectively inhibited the *S. molesta* after two treatments (Supplementary Figs. S18 and S19). In comparison with those in the control treatment, the trichomes on the upper surfaces of the floating leaves of *S. molesta* treated by the hydraulic extracts had significant damage. The wilted trichomes released autotoxic chemicals to enhance the endocidal function of the hydraulic extract. With surfactants, hydraulic extract juice totally eliminated the *S. molesta* within four weeks and no new growth thereafter. The experiment indicates that the juice expressed from fresh matter of *S. molesta* (e.g., by hydraulic press) is effective enough and can be directly used in control of *S. molesta* without concentrating process. All plant tissues of *S. molesta* after hudraulic extraction were dead and there was no new plant growth in the cultured plant residues from the hydraulic extraction within six weeks of observation.

2.3.6. Elimination and Inhibition of Floating Fern (*Salvinia minima* Baker) Plants by H_2O Extracts of the Dried Matter of Giant Salvinia (*Salvinia molesta* D. S. Mitchell)

General Experimental Procedures: Extracts Preparation: See 2.3.4. The H_2O extracts were prepared as experimental solutions at the concentration of 0.1% and 0.5%. Foliar Sprays: The experiment included nine plastic containers (14 × 15 cm, 0.68 L) and each container had 10 g (in fresh weight) of healthy living and floating fern (*Salvinia minima* Baker) (Salviniaceae) cultured in tap water in the greenhouse (30°C during the day time and 20°C at night). There were three treatments. Controls: the *S. minima* plants in each of three containers were sprayed three times with the total of 90 mL H_2O; 0.1% H_2O extracts treatment: the plants in each of three containers were sprayed three times with the total 90 mL of 0.1% H_2O extracts of *S. molesta*; and 0.5% H_2O extracts treatment: the plants in each of three containers were sprayed three times with the total 90 mL of 0.5% H_2O extracts of *S. molesta*. Plant growth and survival status were documented and photographed weekly after the treatments. By the end of 1st, 2nd, 6th weeks, biomass of living plants in each container of all treatments was measured. Statistic Analysis: The data were analyzed by SPSS 13.0 for Windows (SPSS, Inc., Chicago, IL). ANOVA with Duncan post-hoc analysis was used to compare the means of the control and spray treatment groups.

Results: The growth of the plants treated with either 0.1% or 0.5% H_2O extracts of *S. molesta* were significantly inhibited during the first week (Supplementary Fig. S20). By the end of the 6th week, all plants treated by H_2O extracts of *S. molesta* were dead and there was no new growth thereafter.

2.3.7. Elimination and Inhibition of Carolina Mosequito Fern (*Azolla caroliniana* Willd.) Plants by H_2O Extracts of the Dried Matter and Hydraulic Extracts of the Fresh Matter of Giant Salvinia (*Salvinia molesta* D. S. Mitchell)

General Experimental Procedures: Extracts Preparation: H_2O extracts were prepared from the dried matter of *S. molesta* (the preparation method see 2.3.4) and the hydraulic extracts were prepared from the fresh matter of *S. molesta* (see 2.3.5). The H_2O extracts were all prepared as experimental solutions at the concentration of 0.1, 2.5, 5, and 7.5%. Foliar Sprays: Carolina mosequito fern (*Azolla caroliniana* Willd.) (Azollaceae) was collected from East Texas, USA. The experiments were conducted in the greenhouse (30°C during the day time and 20°C at night). The spray experiment started until the plants fully covered the surface of each of the 18 plastic containers (14 × 15 cm, 0.68 L) with 400 mL of tap water each. The plants were randomly classified into six groups: Controls: each of the three containers was spayed three times with 10 mL of H_2O each time; hydraulic extracts treatment: each of the three containers was spayed with 10 mL of hydraulic extracts; 0.1% H_2O extract treatment: each of the three containers was spayed twice with 10 mL of 0.1% H_2O extracts; 2.5% H_2O extracts treatment: each of the three containers was spayed twice with 10 mL of 2.5% H_2O extracts; 5% H_2O extract treatment: each of the three containers was spayed twice with 10 mL of 5% H_2O extracts; and 7.5% H_2O extract treatment: each of the three containers was spayed twice with 10 mL of 7.5% H_2O extracts. By the end of the 5th, 10th, and 15th day, surface coverage of living plants in each container of all treatments was photographed and measured and EC_{50} (half maximal effective concentration) values were calculated by the PROBIT procedure of SPSS 13.0 for Windows. Statistic Analysis: The data were analyzed by SPSS 13.0 for Windows (SPSS, Inc., Chicago, IL). ANOVA with Duncan post-hoc analysis was used to compare the means of the control and spray treatment groups.

Results: Neither the hydraulic extracts nor the H_2O extracts at 0.1% concentration showed any significant impacts on the plant growth of *A. caroliniana* during the first five days of the experiment but both extracts significantly inhibited the plant growth thereafter. The hydraulic

extracts were able to kill 100% of the *A. caroliniana* by the end of the 15th day (Supplementary Fig. S21). The H_2O extracts at higher concentrations (2.5, 5, or 7.5%) significantly inhibited the growth of *A. caroliniana* soon after the treatments. Both 2.5% and 5% H_2O extracts killed 100% of the *A. caroliniana* plants by the end of the experiment. The 7.5% H_2O extracts eliminated all treated plants within the five days of the experiment. EC_{50} value of H_2O extracts of the dried matter of *S. molesta* against *A. caroliniana* is 4.42, 1.32, and 0.16% by the 5th, 10th, and 15th day, respectively.

2.3.8. Elimination and Inhibition of Giant Salvinia (*Salvinia molesta* D. S. Mitchell) Plants by EtOH Extracts of Its Dried Matter

General Experimental Procedures: Extracts Preparation: Air-dried whole plants (550 g) were ground to a coarse powder and percolated two times with 95% EtOH at room temperature (each 3 L, 24 h). The combined EtOH solution was concentrated to give EtOH extracts (34.0 g) under reduced pressure. 1.0 g *S. molesta* EtOH extracts were dissolved in 2 mL DMSO, and then diluted with water to yield 900 mL of 0.1% and 1,000 mL of 0.01% solutions (g/mL) for further experimental analysis. Foliar Sprays: The experiment included 45 healthy and untreated living plants of *S. molesta* (approximately 7 g in fresh weight each). The plants were cultured and tested in the plastic containers (14 × 15 cm, 0.68 L) in the laboratory at room temperature. Controls: 15 plants with five in each container (three replications) were cultured with 300 mL of tap water; 0.01% EtOH extract treatment: 15 plants with five in each container (three replications) were sprayed with 300 mL of 0.01% of the EtOH extracts of *S. molesta* dissolved in H_2O; and 0.1% EtOH extract treatment: 15 plants with five in each container (three replications) were sprayed with 300 mL of 0.1% of the EtOH extracts of *S. molesta* dissolved in H_2O. Plant growth and survival status were documented and photographed weekly after the treatments. By the end of 13th week, new growth biomass of plants in each treatment was measured. The biomass of new growth was a primary factor to measure the inhibition of each treatment on the target plant.

Results: By the end of 13 weeks after the treatment, the plants in both control and 0.01% EtOH extract treatment groups had new growth while about 60% of all *S. molesta* plants treated with 0.1% EtOH extracts of the dried matter of *S. molesta* were dead and no significant growth on the other treated plants although they were still alive.

2.3.9. Impacts of Giant Salvinia (*Salvinia molesta* D. S. Mitchell) Extracts on Other Plant Species

General Experimental Procedures: Extracts Preparation: H_2O extracts were prepared from the dried matter of *S. molesta* (the preparation method see 2.3.4) and EtOH extracts were prepared from the dried matter of *S. molesta* (the preparation method see 2.3.8). Selectivity Tests: The experimental species included two native land fern species, namely, common lay fern (*Athyrium filix-femina* (L.) Mertens var. aspenioides (Michaux) Farwell) (Dryopteridaceae) and southern shield fern (Thelypteris kunthi (Desv.) C.V. Morton (Thelypteridaceae) and some native and invasive aquatic herbaceous species of angiosperms (flowering plants) including knotweed (*Polygonum* sp.) (Polygonaceae), water primrose (*Ludwigia* sp.) (Onagraceae), Coontail (*Ceratophyllum demersum* L.) (Ceratophyllaceae), cattail (*Typha latifolia* L.) (Typhaceae), rush (*Juncus* sp.) (Juncaceae), sedge (*Carex* sp.) Cyperaceae), least duckweed (*Lemna minuta* Kunth) and Brazillan watermeal (*Wolffia brasiliensis* Weddell) (Araceae), water hyacinth (*Eichharnia crassipes*) (Pontederiaceae) and hydrilla (*Hydrilla verticillata* (L.f.) Royle) (Hydrocharitaceae), and three native tree or shrub species of gymnosperms, namely baldcypress (*Taxodium distichum* (L.) Rich.) (Cupressaceae) and loblolly pine (*Pinus taeda* L.) (Pinaceae), buttonbush (*Cephalanthus occidentalis* L.) (Rubiaceae). These species are often associated with *S. molesta* in the Southeastern United States. The experiments were conducted in the greenhouse (30°C during the day time and 20°C at night) or in the field in summer. Each species of least duckweed and Brazilian watermeal was separately cultured in nine plastic containers (14 × 15 cm, 0.68 L) with 400 mL of tap water each. The spray experiment started until the plants fully covered each of the containers. For each of these two species, the plants were randomly classified into three groups: Controls: each of the three containers was spayed three times with 10 mL of H_2O each time; treatment I: each of the three containers was spayed twice with 10 mL of 0.1% H_2O extracts each time and then a week later with 10 mL of 5% of H_2O extracts; and treatment II: each of the three containers was spayed twice with 10 mL of 0.5% H_2O extracts each time and then a week later with 10 mL of 5% H_2O extracts. Surface coverage of living plants in each container of all treatments was photographed and measured weekly for six weeks. Five plants of each species of water hyacinth and hydrilla were cultured separately in container with 50 L tap water. For each species, three containers served as the control group and the plant leaves in each of the other three containers were sprayed twice with the total 100 mL of 5% H_2O extracts each

time. Plant growth and survival status were documented and photographed weekly after the treatments for six weeks. Six 2-year old seedlings of each of bald cypress and loblolly pine in 2-gal pots were used in the experiment. For each species, three seedlings as controls received water only and the leaves (needles) and stems in each of the other three plants were sprayed twice with the total 100 mL of 5% H_2O extracts each time. Plant growth and survival status were documented and photographed weekly after the treatments for six weeks. For all other species, the tests were conducted on young leaves of at least three plants per species were treated in the field when treated *S. molesta* with 5% EtOH extracts of *S. molesta*. Plant growth and survival status were documented and photographed weekly after the treatments for six weeks.

Results: During the six weeks of experiments, none of the testing species was injured by 0.1%, 0.5% or 5% of H_2O extracts (or EtOH extracts) of *S. molesta*. All testing plants of these species stayed in normal growth.

2.4. Inhibition of Seed Germination of Korean Pine (*Pinus koraiensis* Siebold & Zucc.) by Its Seed EtOH Extracts

General Experimental Procedures: Extracts Preparation: Seeds of Korean pine (*Pinus koraiensis* Siebold & Zucc.) (Pinaceae) was purchased from F.W. Schumacher Co, Inc. in Sandwich, MA, USA. 156 g dried *P. koraiensis* seeds were ground to coarse powders and extracted two times for 48 h with 95% EtOH (600 mL each time) at room temperature. Extracts were evaporated under reduced pressure, and 13 g EtOH extracts were obtained. 5 g EtOH extracts were dissolved and suspended in nanopure H_2O and prepared as 100 mL experimental solution at the concentration of 5%. Extracts Yield of Experimental Seeds: Based on the seed weight and extraction rate (8.34%, in dry weight) of the above extraction method, it is estimated that the plant matter for soaking treatment (30 *P. koraiensis* seeds) could produce 1.05 g EtOH extracts. To further reveal the actual amount of the EtOH extracts contained in the plant matter, a more effective extraction of small plant sample (12.6 g) was performed using a ASE 2000 Accelerated Solvent Extractor (60 ^{o}C, 1500 psi, 30 min static time, 100% volume flush, 120 s purge, and 2 cycles). According to this extraction rate, 30 *P. koraiensis* seeds contain at least 1.2 g EtOH extracts. Soaking Treatments and Germination Tests: 270 *P. koraiensis* seeds in total were selected and 30 seeds in a plastic container (14 × 15 cm, 0.68 L) were subjected to one of the following three treatments with three replications per treatment: stored in 4°C and then directly sowed in the pots in greenhouse (control), soaked in 15 mL of nanopure H_2O at room temperature for five weeks

before sowing in the pots, and soaked in a 15 mL of 5% solution of *P. koraiensis* extracts (0.75 g extracts) at room temperature for five weeks before sowing in the pots. All experimental seeds were sowed in the pots with Miracle Grow Potting Mix soil in greenhouse (30°C during the day time and 20°C at night). The seed germination number of each treatment was recorded weekly throughout the experimental period.

Results: By the end of the 9th week, 34.5% seeds without any treatment germinated vs. 2.2% germination among the seeds treated by nanopure H_2O soaking for five weeks. There was no germination among the seeds treated by 5% *P. koraiensis* extracts for five weeks and all of the treated seeds lost viability (Supplementary Fig. S22).

2.5. Elimination and Inhibition of Crested Floating Heart (*Nymphoides cristata* (Roxb.) Kuntze) by Its Extracts

Crested floating heart (*Nymphoides cristata* (Roxb.) Kuntze) (Menyanthaceae) is native to Asia and was introduced to North America through the aquatic plant nursery trade[2]. It is a rooted, floating-leaved dicotyledonous aquatic plant. The plant can quickly cover the water surface with a canopy of leaves, shading out the plants below. The plant is typically found rooted in the submersed sediments in shallow water with its leaves floating on the surface of the water. It can also grow in moist soils but is capable of a free-floating form for a period of time with tuberous propagules attached to the underside of the leaf[3]. The species can be reproduced by various vegetative methods, such as tubers, daughter plants, rhizomes, and fragmentation. Since 1996, the species has become a serious weed problem in the Southeastern United States, from Florida and South Carolina to east Texas, USA. The infestation in Lake Marion, South Carolina has rapidly increased from an initial find of approximately 20 acres in 2006 to over 2,000 acres in 2011[3]. The bio-control by fish, mechanical harvesting, herbicides with fluridone, diquat, 2,4-D, or triclopyr were found ineffective[3]. Control with combinations of glyphosate and imazapyr or endothall alone was shown to be short-lived; numerous re-treatments were required because regrowth often occur within several weeks after the treatment[3]. The successful control of *N. cristata* much depends on the elimination of tubers rather than foliage damage.

2.5.1. Elimination and Prevention of Crested Floating Heart (*Nymphoides cristata* (Roxb.) Kuntze) by Its EtOH Extracts

General Experimental Procedures: Extracts Preparation: The whole plants of *N. cristata* were

collected from Texas, USA. The plants were dried in an oven at 65°C for 48 h. The oven-dried plant matter (3,600 g) was ground to a coarse powder and extracted with 95% EtOH at room temperature twice (each with 20 L and 12 L, respectively) for 48 h each time. The combined EtOH extracts were concentrated under reduced pressure to yield 420 g final extracts. 100 g extracts were dissolved and suspended in H_2O and prepared as 1,000 mL experimental solution at the concentration of 10%. Of the 1,000 mL of 10% experimental extracts, 300 mL was prepared as the 10% extracts solution with 1% surfactant Dyne-Amic (Methyl esters of C16-C18 fatty acids, polyalkyleneoxide, modified polydimethylsiloxane, alkylphenol ethoxylate, Helena Chemical Company, Collierville, TN, USA) (v:v), 150 mL was further diluted into 300 mL with H_2O and then was prepared as the 5% extracts solution with 0.5% Dyne-Amic (v:v), and 100 mL was further diluted into 400 mL with H_2O and then was prepared as the 2.5% extracts solution with 0.5% Dyne-Amic (v:v). Foliar Sprays: Each of the five containers had 300 g living healthy plants of *N. cristata* (with a mixture of mature and young leaves, tubers, daughter plants, rhizomes, and fragmentation) in 25 L of tap water in the greenhouse (30°C during the day time and 20°C at night). Three containers served as controls: one had no treatment, the second one received foliar treatment of 5% EtOH in the amount of 100 mL on each of the first and second days, respectively, and the third one received 1% Dyne-Amic foliar treatment in the amount of 100 mL on each of the first and second days, respectively. Each of the three treatment containers received foliar spray treatment with 10% EtOH extracts with 1% Dyne-Amic, 5% EtOH extracts with 0.5% Dyne-Amic, or 2.5% EtOH extracts with 0.5% Dyne-Amic, in the amount of 100 mL on each of the first and second days, respectively. Living biomass of *N. cristata* plants in each of the containers were weighed weekly after the treatment for six weeks.

Results: During the six weeks of experiments, no plant death or injury was observed in any plants in the containers of the control group, 5% EtOH, or 1% Dyne-Amic (Supplementary Fig. S23). The plants in the foliar application of 10% EtOH extracts with 1% Dyne-Amic started to die on the second day of the treatment and all plants including mature and young leaves, tubers, daughter plants, rhizomes, and fragments in this container were dead by the 4th day. On the 5th day of the treatment, 150 g additional living *N. cristata* plants were placed in this treatment container and all were dead on the next day. The plants in the foliar applications of 5% EtOH extracts with 0.5% Dyne-Amic or 2.5% EtOH extracts with 0.5% Dyne-Amic had slower responses to the treatments. By the end of the 4th week, all plants that were treated by 5% EtOH

extracts with 0.5% Dyne-Amic were dead. Then 88.34% of the plants in the container treated by 2.5% EtOH extracts with 0.5% Dyne-Amic were dead with only 35 g living plants left.

2.5.2. Production of Active H_2O Extracts from Whole Fresh Matter of Crested Floating Heart (*Nymphoides cristata* (Roxb.) Kuntze)

General Experimental Procedures: Extracts Preparation: The 10 kg whole fresh plants of *N. cristata* was placed in each of the three 50 L containers in the greenhouse (30°C during the day time and 20°C at night) and 40 L of tap water was added to each container. During the nine days of extraction, approximately a total of 48 L of H_2O extracts was collected from each container. HPLC analysis of daily chemical changes: 100 mL of H2O extracts was collected daily from the bottom of each of the containers for nine days. After filtration, the H_2O extracts samples were analyzed by HPLC. The HPLC chromatographs of the H_2O extracts were established by Agilent 1100 HPLC system coupled to an Agilent 1100 diode array detector, and an Eclipse XDB-C18 column (4.6 × 150 mm, 3.5 µM) at a flow rate of 0.6 mL/min. A gradient elution was performed by using H_2O (A) and CH_3CN (B) as mobile phases. Elution was performed according to the following conditions: 2% B at time 0, linear increase to 98% B in 22 min, and hold 98% B for 8 min. The column temperature was maintained at 23°C. The HPLC chromatogram was standardized on retention times and peak intensities of the peaks observed at a wavelength of 254 nm and 280 nm. The injection volume was 50 µL for all analyses. The total chemical contents of the H_2O extracts were estimated by the total peak areas of daily samples. Bioassays: 400 mL of the H_2O extracts of *N. cristata* was collected on day 5 of extraction. Of this sample, 200 mL was prepared as the H_2O extracts solution with 0.5% Dyne-Amic (v:v). The remaining 200 mL was autoclaved at 121°C with a pressure of 103.42 kPa (15 psi) using sterilmatic sterilizer (Market Forge Industries Inc, Everett, MA) for 30 min. The autoclaved sample was then prepared as the sterilized H_2O extracts with 0.5% Dyne-Amic (v:v). Each of the three containers had 200 g living healthy plants of *N. cristata* (with a mixture of mature and young leaves, tubers, daughter plants, rhizomes, and fragmentation) in 25 L of tap water in the greenhouse (30°C during the day time and 20°C at night). One container served as a control (without any treatment), one received the foliar application of 200 mL of H_2O extracts of *N. cristata* with Dyne-Amic, and the last container had foliar application of the autoclaved 200 mL of H_2O extracts of *N. cristata* with Dyne-Amic. Living biomass of *N. cristata* plants in each of the containers were weighed weekly after the treatment for six weeks.

Results: According to daily HPLC chromatographic analyses at both 254 and 280 nm, the total chemical diversity and chemical levels in the H_2O extracts from the whole plants were much higher on day 5 than those in the previous four days and were stabilized thereafter (Supplementary Fig. S24). The HPLC analysis shows that there are no significant changes in chemical constituents in H_2O extracts before and after sterilization. The plants treated with either sterilized or nonsterilized H_2O extracts of *N. cristata* plants with 0.5% Dyne-Amic had significant injury on the third day of the foliar treatment. By the end of the 5th week, all plants treated with nonsterilized H_2O extracts were dead and more than 75% of the plants treated with sterilized H_2O extracts were dead in comparison with the healthy plants in the control group.

2.5.3. Production of Active H_2O Extracts from Shredded Fresh Matter of Crested Floating Heart (*Nymphoides cristata* (Roxb.) Kuntze)

General Experimental Procedures: Extracts Preparation: 1 kg whole fresh plants of *N. cristata* was shredded, blended, and placed in each of the three 5 L containers at room temperature and 4 L of tap water was added to the container. During the nine days of extraction, approximately total 4.9 L of H_2O extracts were collected from each container. HPLC Analysis of Daily Chemical Changes: 10 mL of H_2O extracts were collected daily from the bottom of the container for nine days. After filtration, the H_2O extracts samples were analyzed by HPLC. The HPLC chromatographs of the H_2O extracts were established by Agilent 1100 HPLC system coupled to an Agilent 1100 diode array detector, and an Eclipse XDB-C18 column (4.6 × 150 mm, 3.5 μM) at a flow rate of 0.6 mL/min. A gradient elution was performed by using H_2O (A) and CH_3CN (B) as mobile phases. Elution was performed according to the following conditions: 2% B at time 0, linear increase to 98% B in 22 min, and hold 98% B for 8 min. The column temperature was maintained at 23°C. The HPLC chromatogram was standardized on retention times and peak intensities of the peaks observed at a wavelength of 254 nm and 280 nm. The injection volume was 50 μL for all analyses. The total chemical contents of the H_2O extracts were estimated by the total peak areas of daily samples. Bioassays: 200 mL of the H_2O extracts of *N. cristata* collected on the day 1 of extraction and was prepared as the H_2O extracts with 0.5% Dyne-Amic (v:v). Each of the two containers had 200 g living healthy plants of *N. cristata* (with a mixture of mature and young leaves, tubers, daughter plants, rhizomes, and fragmentation) in 25 L of tap water in the greenhouse (30°C during the day time and 20°C at night). One container served as a control (without any treatment), one received the foliar application of 200 mL of H_2O extracts of

N. cristata with Dyne-Amic. Living biomass of *N. cristata* in each of the containers were weighed weekly after the treatment for six weeks.

Results: According to daily HPLC chromatographic analyses at both 254 and 280 nm, the total chemical diversity and levels in the H_2O extracts reached relatively stable point on day 3, much quicker than those in the whole plant extraction (Supplementary Fig. S25). The plants treated with H_2O extracts of *N. cristata* from the first day of collection with Dyne-Amic had significant injury on the third day of the foliar treatment. By the end of the 4th week, all plants in these containers were dead in comparison with the healthy plants in the control group.

2.5.4. Selective Toxicity of EtOH and H_2O Extracts of Crested Floating Heart (*Nymphoides cristata* (Roxb.) Kuntze) against Crested Floating Heart over Giant Salvinia (*Salvinia molesta* D. S. Mitchell), Caroliana Mosquito Fern (*Azolla caroliniana* Willd), Least Duckweed (*Lemna minuta* Kunth), Brazilian watermeal (*Wolffia brasiliensis* Weddell), and Water Lettuce (*Pistia stratiotes* L.)

General Experimental Procedures: Extracts Preparation: The 10 kg whole fresh plants of *N. cristata* was placed in each of the three 50 L containers in the greenhouse (30°C during the day time and 20°C at night) and 40 L of tap water was added to each container. During the nine days of extraction, approximately a total of 48 L of H_2O extracts was collected from each container. The experimental H_2O extracts were obtained from the containers on the fifth day. 200 mL of the H_2O extracts were prepared as the H_2O extracts with 0.5% Dyne-Amic (v:v). Preparation of the EtOH extracts of *N. cristata*: The whole plants of *N. cristata* were collected from East Texas, USA. The plants were dried in an oven at 65°C for 48 h. The oven-dried plant matter (3,600 g) was ground to a coarse powder and extracted with 95% EtOH at room temperature twice (each with 20 L and 12 L, respectively) for 48 h each time. The combined EtOH extracts were concentrated under reduced pressure to yield 420 g final extracts. 200 mL of 2.5% EtOH extracts with 0.5% Dyne-Amic (v:v) and 200 mL of 5% EtOH extracts with 0.5% Dyne-Amic (v:v) were prepared separately. Selectivity Tests: Each of the six containers had 300 g living healthy plants of *N. cristata* (with mixtures of mature and young leaves, tubers, daughter plants, rhizomes, and fragmentation), 15 g *S. molesta* of the tertiary stage, 5 g *S. molesta* of the primary stage, 5 g *A. caroliniana*, three plants (approximately 30 g) of water lettuce (*Pistia stratiotes* L., Araceae), and 5 g mixture of least duckweed (*Lemna minuta*, Araceae) and Brazilian watermeal (*Wolffia brasiliensis* Waddell) in 25 L tap water in the greenhouse (30°C during the day time and 20°C at

night). One container served as a control without any treatment. Four treatment containers each received either foliar spray treatment with H_2O extracts, H_2O extracts with Dyne-Amic, 2.5% EtOH extracts with 0.5% Dyne-Amic, or 5% EtOH extracts with 0.5% Dyne-Amic, in the amount of 100 mL on each of the first and second days, respectively. For another container, a total of 400 mL (200 mL each of the first and second days) of H_2O extracts (without surfactant) was slowly injected into the water (approximately 5 cm from the surface) by a pipette. Living biomass of *N. cristata* in each of the containers were weighed weekly after the treatment for six weeks.

Results: During the six weeks of experiments, no death or injury of any species was observed in any plants in the control group (Supplementary Fig. S26). In the foliar treatments of H_2O extracts with Dyne-Amic, 2.5% EtOH extracts with 0.5% Dyne-Amic, or 5% EtOH extracts with 0.5% Dyne-Amic, all plants of *S. molesta*, *A. caroliniana*, and *P. stratiotes* sunk into the water and died during the first three days of the experiments. All *N. cristata* plants in these three treatments were dead although some dead leaves were still green in color and floated on the water surface by the end of the 2nd week. At the end of experiment, all plants of *N. cristata*, *S. molesta*, and *P. stratiotes* were dead and decomposed but *L. minuta* plants grew well in all three treatments with *W. brasiliensis* survived in the container treated with H_2O extracts with 0.5% Dyne-Amic.

In both foliar and injection treatments with the H_2O extracts (without surfactant), all *L. minuta* and *W. brasiliensis* grew well. In the foliar treatment with 200 mL of the H_2O extracts (without surfactant), three plants of *N. cristata* were dead and a few others had some injury and approximately 30% of *A. caroliniana* plants were dead by the end of second week. Some plants of both *S. molesta* and *P. stratiotes* had some injury but soon had new growth during the experiment. All *N. cristata* plants in the treatment of injection of the total 400 mL of H_2O extracts (without surfactant) were dead except one plant by the end of second week of experiment. All other species in this treatment were still green in color with significant injury in all submerged tissues of *S. molesta*, *P. stratiotes*, and *A. caroliniana*. However, during the third week, new plants had soon reproduced from these injured plants.

2.5.5. Selective Toxicity of EtOH Extracts of Crested Floating Heart (*Nymphoides cristata* (Roxb.) Kuntze) against Crested Floating Heart over Muskgrass (*Chara vulgaris* L.) and Bladderwort (*Utricularia macrorhiza* LeConte)

General Experimental Procedures: Extracts Preparation: The whole plants of *N. cristata* were collected from East Texas, USA. The plants were dried in an oven at 65°C for 48 h. The oven-dried plant matter (70.3 g) was ground to a coarse powder and extracted with 95% EtOH at room temperature twice (each with 1,000 mL each) for 48 h each time. The combined EtOH extracts were concentrated under reduced pressure to yield 4.5 g final extracts. Selectivity Tests: The treatment experiments were conducted in the greenhouse (30°C during the day time and 20°C at night). 60 g *C. vulgaris*, 10 g *N. cristata*, and 2 g *U. macrorhiza* were placed in each of the six plastic containers (23 × 23 cm, 2.7 L) with 1,200 mL of tap water each. 1.5 g *N. cristata* extracts were placed into the water of each of the three containers while three other containers received no treatment to serve as the control group. Living biomass of each species in each of the containers were weighed 48 h after the treatment.

Results: All leaves and stems of the plants *of N. cristata* treated by *N. cristata* extracts were dead within 48 h of the treatment and only one or two rhizomes were still alive (2.13±2.23 g) in comparison with the vigorous plants in the control group (with average living biomass at 11±0.5 g). The biomass weight of *C. vulgaris* did not showed significant difference between *N. cristata* extracts treatments and controls (59.3±2.03 g vs. 59.5±13.5 g, respectively) but about 40-50% of the *C. vulgaris* plants lost green color in the treatment group. There is no measurable or observable difference for *U. macrorhiza* between the *N. cristata* extracts treatments and controls.

2.6. Elimination and Inhibition of Palmer's Pigweed (*Amaranthus palmeri* S. Wats.) by EtOH Extracts of Its Whole Plants

Palmer's pigweed, Palmer's amaranth or carelessweed (*Amaranthus palmeri* S. Wats.) of the pigweed family (Amaranthaceae) is native to the North American Southwest, from southern California to Texas, USA and northern Mexico. It is a fast-growing and highly competitive invasive species. The species is naturalized in eastern North America, Europe, Australia, and other areas. It is a well-known weed in agricultural crop fields. The weed is considered a threat most specifically to the production of genetically modified cotton and soybean crops because the plant has developed resistance to glyphosate, a widely-used broad-spectrum herbicide.

2.6.1. Elimination and Inhibition of Palmer's Pigweed (*Amaranthus palmeri* S. Wats.) by EtOH Extracts of Its Whole Plants

General Experimental Procedures: Extracts Preparation: The whole plants of Palmer's pigweed

(*Amaranthus palmeri*) were collected from a soybean field in the central Louisiana, USA where *A. palmeri* survived from a recent glyphosate application (Supplementary Fig. S27). The plants were dried in an oven at 65°C for 48 h. 3,200 g dried plant matter was ground to a coarse powder and extracted two times for 48 h each with 95% EtOH (20 L and 12 L, respectively) at room temperature. The combined EtOH extracts were concentrated under reduced pressure and a total of 66 g extracts were obtained. 30 g extracts were dissolved and suspended in H_2O and prepared as a 300 mL experimental solution at the concentration of 10% extracts. Before treatment, 300 mL of 10% H_2O solution was prepared as the 10% extracts with 1% surfactant Tergitol 15-S-9 (Sigma-Aldrich Co., St. Louis, MO, USA) (v:v). Foliar Sprays: 20 healthy glyphosate-resistant plants (c. 30 cm foliage height) of Palmer's pigweed in the field (in the site of plant matter collection) were selected for the experiment. 10 plants served as the controls without any treatment while the other 10 plants were sprayed with 100 mL of 10% EtOH extracts of pigweeds with 1% Tergitol 15-S-9 twice in the first month. In each treatment, the plants of big-rooted morning glory or wild potato vine (*Ipomoea pandurata* (L.) G.F.W. Mey.) (Convolvulaceae) associated with the pigweed were also treated. The plant heights and living biomass of plants in the control or spray treatment were measured by the end of the two-month experiment.

Results: Some leaves and flower spikes of the 10 treated pigweed plants with 10% EtOH extracts of pigweeds with 1% Tergitol were injured five days after the treatments. The EtOH extracts from glyphosate-resistant *A. palmeri* can eliminate glyphosate-resistant *A. palmeri* on the same site within 40 days. At the same dosage, the extracts had no significant damage on the associated *I. pandurata* (Supplementary Fig. S27). The foliage height of the plants in the control group reached 52 cm at average and all produced spikes of seeds.

2.6.2. Phytotoxicity of Palmer's Pigweed (*Amaranthus palmeri* S. Wats.) EtOH Extracts

General Experimental Procedures: Extracts Preparation: The EtOH extracts were prepared from glyphosate-resistant plants of Palmer's pigweed (*A. palmeri*) in the central Louisiana, USA (see 2.6.1). 1 g EtOH extracts of *A. palmeri* were prepared in 10 mL experimental solution with nanopure H_2O at the concentration of 10%. Bioassays: The experimental plants were 3-week-old *A. palmeri* seedlings germinated from the seeds collected from the above described glyphosate-resistant plants. The seedlings were grown in pots with Miracle Grow Potting Mix soil in the

greenhouse (30°C during the day time and 20°C at night). The intact seedling plants of 3-week old *Q. texana* and 2-month-old *L. styraciflua* grown in pots in greenhouse were also tested in this experiment. 10 µL of 0.5% glyphosate and 10% EtOH extracts of *A. palmeri* were applied seprately by pipet on the upper and lower leaf surfaces of three plants of each species, respectively. For each plant species, three upper leaves and three lower leaves were treated separately. The leaf surfaces of the experimental plants were checked daily for two weeks by a ×60 portable microscope linked to an iPhone^{TM}.

Results: Of the three testing species, only *A. palmeri* had no injury by 10 µL of 0.5% glyphosate. The tissues in the leaf spots of *A. palmeri* treated with 10 µL 10% EtOH extracts of *A. palmeri* by either upper or lower surface applications were totally destroyed within two weeks (Supplementary Fig. S28).

2.7. Elimination and Inhibition of Alligator Weed (*Alternanthera philoxeroides* Griseb.) by Its EtOH Extracts

Alligator weed *Alternanthera philoxeroides* Griseb.) (Amaranthaceae) is another invasive member of the pigweed family. It is native to South America but has become an important invasive species in many parts of the world. The species can grow in a variety of habitats, it can survive on dry land, and it can also form dense tangled mats floating on the surface of a body of water and thus block waterways and threaten native species.

General Experimental Procedures: Extracts Preparation: The whole plants of alligator weed were collected from Nacogdoches, Texas, USA. The plants were dried in an oven at 65°C for 48 h. 750 g dried plant matter was ground to a coarse powder and extracted two times for 48 h each with 95% EtOH (12 L and 10 L, respectively) at room temperature. The combined EtOH extracts were concentrated under reduced pressure and the total 43 g extracts were obtained. 30 g extracts were dissolved and suspended in H_2O and prepared as 300 mL experimental solution at the concentration of 10% with 0.25% Dyne-Amic (v:v) and 0.125% Kinetin (v:v). Foliar Sprays: Foliar application of 30 mL of alligator weed extracts with surfactant was made on 0.25 m^2 of alligator weed plants (22 plants) in the field. The foliar applications were also on *T. sebifera* and a species knotweed (*Polygonum* sp.) at the same time. The status of the test plants were documented and photographed daily after the treatment.

Results: The treated *A. philoxeroides* plants had significant injury with two plant deaths five days after being treated with 10% EtOH extracts of alligator weed extracts with surfactants (Supplementary Fig. S29). No leaf injury was found on either *T. sebifera* or *Polygonum* sp.

2.8. Elimination and Inhibition of Nopal Cactus (*Opuntia ficus-indica* (L.) Mill.) Stems by Their EtOH Extracts

General Experimental Procedures: See the method section of "Induced Mutations of Nopal Cactus (*Opuntia ficus-indica* (L.) Mill.) Stems by Their EtOH Extracts" in the text. The living status of individuals was recorded once every week throughout the experimental period.

Results: By the end of second month, all stems of *O. ficus-indica* in the control group alive while only two of the six stems treated with *O. ficus-indica* extracts survived.

2.9. Inhibition of Seed Germination of Broccoli (*Brassica oleracea* L.) by Its Seed EtOH Extracts

General Experimental Procedure: Preparations of Seed Extracts: See the method section of "Morphological Mutations Induced by External Applications of Extracts: *Brassica oleracea*)" in the text. Soaking Treatments and Germination Tests: 2,700 *B. oleracea* sound seeds were selected and 300 seeds in a Petri dish were subjected to one of the following soaking treatments at room temperature with three replications per treatment: a 10 mL of nanopure H_2O for 48 h, a 10 mL of 5% solution of *B. oleracea* seed extracts (0.5 g extracts) for 48 h, and a 10 mL of 5% solution of *B. oleracea* seed extracts (0.5 g extracts) for two weeks. Seed germination in each dish was surveyed by the end of soaking treatment. Then the seeds were sowed in germination box with soil (50 seeds per box) in the greenhouse (30°C during the day time and 20°C at night). The number of germinated individuals and cotyledon number, leaf morphology, and stem number were recorded once every week throughout the 4-week experimental period.

Results: In the water soaking treatment, *B. oleracea* seeds started to germinate soon and approximately 88.5% seeds had germinated in Petri dishes within 48 h. But there was no seed germination in both extracts treatments in Petri dishes during the soaking treatments (48 h or two weeks). By the end of four weeks of germination culture in the soil, only 58.7% seedlings developed from the *B. oleracea* seeds soaked in water and about 30% could not survive after germination because of the newly developed shoots were damaged during the seed sowing process. After removal of the *B. oleracea* seed extracts, 67.8% and 0.2% of *B. oleracea* seeds

developed seedlings after soaked in 5% *B. oleracea* seed extracts for 48 h and for two weeks, respectively. All ungerminated seeds in the extracts treatments lost viability.

2.10. Elimination and Inhibition of Kudzu (*Pueraria lobate* (Wild.) Ohwi) by Its EtOH Extracts

Kudzu (*Pueraria montana* (Lour.) Merr., also known as *P. lobata* (Wild.) Ohwi or *P. montana* (Lour.) Merr. var. *lobata* (Willd.) Maesen & S. Almeida) (Fabaceae) is also known as Japanese arrowroot. It is native to Asia but becomes one of the most common invasive vine species throughout the Southeastern United States. Kudzu can be reproduced by seeds, runners that root at the nodes to form new plants or rhizomes. The plant can climb over trees or shrubs and grows so rapidly that it can cover and kill them by heavy shading.

General Experimental Procedures: Extracts Preparation: The whole plants of Kudzu were collected from Nacogdoches, Texas, USA. The plants were dried in an oven at 65°C for 48 h. 4,000 g dried plant matter was ground to a coarse powder and extracted two times for 48 h each with 95% EtOH (20 L and 12 L, respectively) at room temperature. The combined EtOH extracts were concentrated under reduced pressure and a total of 142.5 g extracts were obtained. 100 g extracts were dissolved and suspended in H_2O and prepared as 1,000 mL experimental solution at the concentration of 10% extracts. 25 g extracts were dissolved and suspended in nanopure H_2O and prepared as 500 mL experimental solution at the concentration of 5% extracts. Foliar Sprays: Three plots (0.5 m^2 each) of kudzu plants on the ground in the field were selected for the foliar applications. One plot served as a control without any treatment, one was treated by 150 mL of 5% kudzu extracts, and the third plot was treated with 150 mL of 10% kudzu extracts. Foliar application of 150 mL of 10% EtOH extracts of kudzu was also made on some nearby species including water elm (*Planera aquatica* J.F. Gmel.) (Ulmaceae), Japanese honeysuckle (*Lonicera japonica* Thunb.) (Caprifoliaceae), and golden bamboo (*Phyllostachys aurea* Riviere & C. Riviere) (Poaceae). The plant status was documented and photographed daily after the treatment.

Results: The EtOH extracts of *P. montana* had significant effects on *P. montana* (Supplementary Fig. S30). 5% EtOH extracts killed more than 50% of the plants a week after the treatment while 10% EtOH extracts eliminated about 80% of the treated plants at the same time period. At the same dosage, *P. montana* extracts did not impact on any other species including *P. aquatica*, *L.*

japonica, and *P. aurea*.

2.11. Elimination and Inhibition of Chinese Tallow (*Triadica sebifera* (L.) Small) Seedlings by Its Extracts

Chinese tallow (*Triadica sebifera*) is native to China and is now a very invasive species in the Southeastern United States. Although manual and mechanical, environmental/cultural, chemical, and biological methods have been used, the control of this species has not been successful. The EtOH extracts of the fruits of *T. sebifera* inhibited and even killed young *T. sebifera* seedlings. However, the *T. sebifera* extracts did not cause any damage in the seedlings of Chinese privet (*Ligustrum sinense* Lour.) of the family Oleaceae. Further, the *L. sinense* extracts did not damage the *T. sebifera* seedlings.

2.11.1. Elimination and Inhibition of Seedlings of Chinese Tallow (*Triadica sebifera* (L.) Small) by EtOH Extracts of Chinese Tallow Fruits

General Experimental Procedures: Extracts Preparation: The fruits of *T. sebifera* (260 g, in dry weight) and Chinese privet (*Ligustrum sinense* Lour.) (family Oleaceae) (400 g, in dry weight) were ground to coarse powders and extracted two times with 70% EtOH at room temperature (2 L each, 48 h), respectively. After evaporated under reduced pressure 27 g EtOH extracts of *T. sebifera* fruits and 25.3 g EtOH extracts of *L. sinense* fruits (both in dry weight) were obtained, respectively. Both extracts were dissolved in nanopure H_2O separately and then was prepared as experimental solutions at the concentration of 10%. Foliar Sprays: The spray treatment experiment included 18 *T. sebifera* seedlings (three weeks old) and 12 *L. sinense* seedlings under the parent trees in the field. There were four treatments. Controls: six seedlings of each species were sprayed with 3 mL of tap water; *T. sebifera* extracts treatment on *T. sebifera* seedlings: six *T. sebifera* seedlings were sprayed with 3 mL of 10% *T. sebifera* fruit extracts; *T. sebifera* extracts treatment on *L. sinense* seedlings: six *L. sinense* seedlings were sprayed with 3 mL of 10% tallow tree fruit extracts; and *L. sinense* extract treatment on *T. sebifera* seedlings: six *T. sebifera* seedlings were sprayed with 3 mL of 10% *L. sinense* fruit extracts. Plant growth and survival status were documented and photographed daily after the treatments.

Results: All six *T. sebifera* seedlings treated by the EtOH extracts of *T. sebifera* fruits were significantly damaged. During the one week of experiments, two *T. sebifera* seedlings were dead

and four were severely injured. However, the *T. sebifera* extracts did not cause any damage in *L. sinense* seedlings while the *L. sinense* extracts did not damage the *T. sebifera* seedlings.

2.11.2. Elimination and Inhibition of Seedlings of Chinese Tallow (*Triadica sebifera* (L.) Small) by EtOH Extracts of Chinese Tallow Stems

General Experimental Procedures: Extracts Preparation: The stems of *T. sebifera* were collected from Nacogdoches, Texas, USA, and were dried in an oven at 65°C for 48 h. 400 g dried stems were ground to coarse powders and each were extracted two times for 48 h with 95% EtOH (2 L and 2 L, respectively) at room temperature. After evaporated under reduced pressure 41 g EtOH extracts were obtained. 30 g each extracts were dissolved and suspended in nanopure H_2O and then prepared as 300 mL experimental solutions at the concentration of 10% extracts. 100 mL of solution was prepared as 10% EtOH extracts with 0.25% Inlet (v:v) and 0.125% mL of Kinetin (v:v). Foliar Sprays: *Triadica sebifera* seedlings (<10 cm in height, approximately three weeks old, germinated from seeds) in the field were selected for the experiment. Six seedlings served as the controls without any treatment, six seedlings were sprayed with 15 mL of 10% EtOH extracts of *T. sebifera* stems two times on the 1st and 3rd days, respectively, and six seedlings were sprayed with 15 mL of 10% EtOH extracts of *T. sebifera* stems with surfactants two times on the 1st and 3rd days, respectively. The same foliar applications were also applied on the seedlings of Nuttall oak or Texas red oak (*Quercus texana* Buckley) (Fagaceae), Japanese honeysuckle (*Lonicera japonica* Thunb.) (Caprifoliaceae), Chinese privet (*Ligustrum sinense* Lour.) (Oleaceae), muscadine grape (*Vitis rotundifolia* Michx.) (Vitaceae), and sugarberry (*Celtis laevigata* Willdenow) (Cannabaceae). Plant statuses were documented and photographed daily for two months after the treatments.

Results: During the two months of foliar treatment experiments, all six seedlings of *T. sebifera* in the control group grew well. Of the seedlings treated with the EtOH extracts of the *T. sebifera* stems (without surfactants), two were dead within days and others were significantly injured (Supplementary Fig. S31). A newly emerged oak seedling showed no damage from the extracts (Supplementary Fig. S32). Two *T. sebifera* seedlings were significantly injured and four were killed by the EtOH extracts of the *T. sebifera* stems with surfactants. During the same period, a treated seedling of Chinese privet grew well.

2.11.3. Elimination and Inhibition of Coppiced Plants and Saplings of Chinese Tallow (*Triadica sebifera* (L.) Small) by the EtOH Extracts of Chinese Tallow Leaves and Stems

General Experimental Procedures: Extracts Preparation: The leaves and stems of *T. sebifera* were collected from Nacogdoches, Texas, USA, in October 2014 and were dried in an oven at 65°C for 48 h. 14 kg dried leaves and stems were ground to coarse powders and each were extracted two times for 48 h with 95% EtOH (40 L and 24 L, respectively) at room temperature. Extracts were evaporated under reduced pressure. 646 g EtOH extracts were obtained. The treatment experiments were conducted in a field in East Texas, USA. Foliar Applications to Coppiced Plants: 10 g extracts were dissolved and suspended in H_2O and prepared as 100 mL experimental solution at the concentration of 10% extracts with 1% vegetable (soybean) oil (Great Value, Walmart) (v:v). 12 young coppiced plants (shoots of about 60 cm in height) of *T. sebifera* in the field were selected for the experiment. Four plants served as the controls without any treatment, four treated with 50 mL of soybean oil each only, and four plants were sprayed with 50 mL of 10% EtOH extracts with vegetable oil two times on the 1st and 3rd days. The same foliar applications were also applied on the seedlings of Nuttall' oak or Texas red oak, Japanese honeysuckle, Chinese privet, muscadine grape, and sugarberry. Plant growth and survival status were documented and photographed daily for three months after the treatments. Hack and Squirt Applications to Saplings: approximately 400 g *T. sebifera* EtOH extracts were dissolved and suspended in bark oil EC Blue (UAP Distribution, Inc., Greeley, CO) to prepare 2,000 mL experimental solution with 20% extracts. 30 saplings (1-2 m tall, with DBH 10-20 mm) in the field were randomly classified into three groups with 10 plants each. All plants were hacked twice using a hatchet. The first group served as the controls without any treatment, each of the second group had 5 mL of bark oil squirted into the hack with a squirt bottle, and each of the third group was sprayed with 5 mL of 20% *T. sebifera* EtOH extracts squirted into the hack spot with a squirt bottle. The plant status was evaluated and photographed monthly after the treatment**.**

Results: During the three months of coppiced plants experiments, all four plants of *T. sebifera* in the control group grew well. Among the four coppiced plants treated with EtOH extracts of *T. sebifera* leaves and stems with soybean oil, most of the leaves fell with one dead stem within 11 weeks. During the experiments, other plant species showed no damage by the extracts. By the end of the second month of the experiment, all 10 plants treated with *T. sebifera* extracts in the

hack and squirt applications were significantly injured with six plants dead above ground. During the same time period, the trees in the control group had no significant change, while those treated with bark oil had about 10-20% of the leaves turn yellow or brown.

2.11.4. Impacts of EtOH Extracts of Chinese Tallow (*Triadica sebifera* (L.) Small) Leaves and Stems on Other Selected Plant Species

General Experimental Procedures: Extracts Preparation: The EtOH extracts of the leaves and stems of *T. sebifera* were prepared as described in the above experiment. EtOH extracts of *T. sebifera* were prepared in 1% experimental solution with nanopure H_2O. Bioassays: The intact seedling plants of *T. sebifera, Q. texana,* and *L. styraciflua* grown in the greenhouse were tested in this experiment. 10 µL 1% EtOH extracts of *T. sebifera* were applied by pipet on the upper and lower leaf surfaces of the intact plants, respectively. For each plant species, three upper leaves and three lower leaves were treated separately. The leaf surfaces of the experimental plants were checked daily for two weeks by a ×60 portable microscope linked to an iPhone^{TM}.

Results: Among all three tested species, only *T. sebifera* leaves were damaged by either upper or lower leaf surface treatment with 10 µL 1% EtOH extracts of *T. sebifera*. Neither *Q. texana* or *L. styraciflua* had any injury following the treatments with the extracts physically accumunation on the leaf surfaces.

2.12. Elimination and Inhibition of Hogwort (*Croton capitatus* var. *lindheimeri* (Engelm. & Gray) Muell.-Arg.) by Its EtOH Extracts

Hogwort (*Croton capitatus* var. *lindheimeri* Michx. var. *lindheimeri* (Engelm. & Gray) Muell.-Arg.) (Euphorbiaceae) is an annual herbaceous plant also commonly known as woolly croton or Lindheimer's hogwort in the central and eastern USA. The plant is poisonous to livestock and honey made from its nectar can be toxic to humans. Although it is a native plant to the USA, the plant is very invasive and becomes a nuisance for pastures and cultivated fields. Croton was controlled by mowing and manual removal, as no herbicide was available which would not also harm the planted forages.

General Experimental Procedures: Extracts Preparation: The whole plants of *C. capitatus* var. *lindheimeri* were collected from Nacogdoches, Texas, USA. The plant sample was dried in an oven at 65°C for 48 h. 2,000 g dried plant matter was ground to a coarse powder and extracted two times for 48 h each with 95% EtOH (20 L and 12 L, respectively) at room temperature. The

combined EtOH extracts were concentrated under reduced pressure and a total of 53 g extracts were obtained. 15 g extracts were dissolved and suspended in nanopure H_2O and prepared as 150 mL experimental solution at the concentration of 10% extracts with 1% Tergitol 15-S-9 (Sigma-Aldrich Co., St. Louis, MO, USA) (v:v). Foliar Sprays: In the field, 10 *C. capitatus* var. *lindheimeri* plants (about 50 cm in height) were selected for the experiments. Of the plants, five individuals served as the controls without any treatment. Five plants were sprayed with the *C. capitatus* var. *lindheimeri* extracts with 1% Tergitol at the amount 15 mL per plant. The same foliar applications were also applied to five plants nearby of the following species: three-seeded mercury (*Acalypha rhomboidea* Raf.), another native weed of the same spurge family (Eurphorbiaceae) in North America; cultivated monocots bermudagrass (*Cynodon dactylon* (L.) Pers.) (Poaceae); and turmeric (*Curcuma longa* L.) (Zingiberaceae). The status of each treated plant was evaluated and photographed every daily after the treatments.

Results: During the four weeks of observations, the five *C. capitatus* var. *lindheimeri* plants in the control group grow normally. For the five *C. capitatus* var. *lindheimeri* plants treated with 10% extracts with 1% Tergitol, most leaves folded and had significant injury on the second day of the treatments. By the end of the first week after the treatments, two plants were dead and the other three plants had leaves that became wilt and turn yellow (vs. the green leaves of the plants in the control group). However, during the experiments the treated *A. rhomboidea* and monocots had no visible injury caused by the *C. capitatus* var. *lindheimeri* extracts treatment (Supplementary Fig. S33).

2.13. Elimination and Inhibition of Chaya (*Cnidoscolus aconitifolius* (Mill.) I.M. Johnst.) by Its EtOH Extracts

General Experimental Procedures: Extracts Preparation: The stems and leaves of chaya (*Cnidoscolus aconitifolius* (Mill.) I.M. Johnst.) (Eurphorbiaceae) (400 g in dry weight) were ground to a coarse powder and extracted two times for 48 h each with 95% EtOH (2.5 L each time) at room temperature. The combined extracts were concentrated to give 24.6 g under reduced pressure. The 5 g extracts were dissolved in nanopure H_2O and prepared as 100 mL experimental solution at the concentration of 5% EtOH extracts of *C. aconitifolius*. Soaking Experiment: The treatment experiments were conducted in laboratory and greenhouse. 12 pieces of *C. aconitifolius* stems (12-14 cm long) with leaves were prepared and subjected to two

treatments. Six pieces of stems were cultivated in 100 mL of nanopure H_2O to serve as the controls and six were cultivated in 100 mL of 5% EtOH extracts of *C. aconitifolius* for 12 days at room temperature. Growth and Propagation Tests: Each experimental stem was placed in one-gallon pots with Miracle Grow Potting Mix soil in the greenhouse. The living status of individuals was recorded once every week throughout the experimental period.

Results: By the end of second month, two of the six stems of *C. aconitifolius* in the control group alive while none under the treatment with *C. aconitifolius* extracts survived.

2.14. Elimination and Inhibition of Yaupon (*Ilex vomitoria* Sol. Ex Alton.) by Its EtOH Extracts or Fractions

Yaupon (*Ilex vomitoria*), also known as yaupon holly, is native to Southeastern United States. The evergreen shrub species is adapted to a wide range of soils and climate conditions. It has been widely cultivated as a landscape species, particularly as a hedge plant. However, yaupon is aggressive and is capable of forming dense pure stands which limit grass and forb production, timber species seedling establishment, and species diversity. Therefore, the species is often considered an unwanted species in the forest or range management of the Southeastern United States. Yaupon is easily top-killed by burning, but can sprout from the base.

2.14.1. Inhibition and Elimination of Yaupon (*Ilex vomitoria* Sol. Ex Alton.) by EtOH Extracts of Yaupon Leaves and Stems

General Experimental Procedures: Extracts Preparation: The leaves and stems of yaupon (*Ilex vomitoria*) were collected from Nacogdoches, Texas, USA. The leaves and stems were dried in an oven at 65°C for 48 h, separately. The dried leaf and stem samples (3,300 g) were ground to a coarse powder and extracted two times for 48 h each with 95% EtOH (30 L and 23 L, respectively) at room temperature. The combined EtOH extracts were concentrated under reduced pressure and a total of 265 g extracts were obtained. Foliar Sprays: 10 g extracts was prepared as 100 mL experimental solution with nanopure H_2O at the concentration of 10% extracts with 1% Firezone surfactant (Helena Chemical Company, Collierville, TN, USA). 10 plants of *I. vomitoria* with a diameter at base of <10 mm were selected for foliar treatments. Five plants without any treatment served as the controls and each of the other five plants was sprayed with 20 mL of *I. vomitoria* EtOH extracts with 1% Firezone. Other Treatments: 225 g the extracts were dissolved and suspended in bark oil EC Blue (UAP Distribution, Inc., Greeley,

CO) and prepared as 900 mL experimental solution at the concentration of 25% leaf and stem extracts of *I. vomitoria*. The treatment experiments were conducted in the field in Nacogdoches, Texas, USA. *Hack and Squirt Applications*: 15 plants of *I. vomitoria* with a diameter at base ranging from 10 to 20 mm were selected for hack and squirt application. Five plants without any treatment served as the controls. Five plants were cut in the trunk using a hatchet and each was squirted with 8 mL of *I. vomitoria* EtOH extracts with a squirt bottle. Five plants had the hack and squirt application with bark oil only. *Cut Stump Treatments*: 15 *I. vomitoria* plants were cut down at about 15 cm above the ground. Five of them without any treatment were used as the controls. Five were treated with *I. vomitoria* EtOH extracts onto the cut stumps at the amount of 8 mL per stump. Another five plants were treated with bark oil only at the amount of 8 mL per stump. The plant status was evaluated and photographed weekly after the treatments.

Results: By the end of four weeks of experiments, the *I. vomitoria* plants in the control group or treatment group with oil in the hack and squirt applications had no observable changes. Some leaves of the plants treated by the foliar applications of 10% *I. vomitoria* extracts with 1% Firezone had injuries. In the hack and squirt treatments, all five *I. vomitoria* plants treated with *I. vomitoria* extracts with bark oil had terminal shoots and some leaves were dead or had significant damage by the end of four weeks and all except one were dead by February next year. No sprouts had come out from any stumps treated by the *I. vomitoria* extracts with bark oil by February next year.

2.14.2. Inhibition and Elimination of Yaupon (*Ilex vomitoria* Sol. Ex Alton.) by t-BuOH Fraction of EtOH Extracts of Yaupon Fruits

General Experimental Procedures: Extracts Preparation: The ripen fruits of yaupon (*Ilex vomitoria*) were collected from a single tree in Nacogdoches, Texas, USA. The fruits were dried in an oven at 65°C for 48 h, separately. The dried fruits samples (7.5 kg) were ground to a coarse powder and extracted three times for 48 h each with 20 L 95% EtOH each at room temperature. The combined EtOH extracts were concentrated under reduced pressure and a total of 430 g extracts were obtained. The extracts were suspended in H_2O and partitioned with EtOAc first and then t-BuOH and obtained three fractions: H_2O fraction (335 g), EtOAc-soluble fraction (42 g) and t-BuOH-soluble fraction (53 g). The 5 g each of the extracts, H_2O fraction, EtOAc fraction, and t-BuOH fraction were dissolved in nanopure H_2O separately and prepared as 50 mL experimental solution with nanopure H_2O at the concentration of 10%, respectively. Bioassays:

The experiment was conducted in the greenhouse (30°C during the day time and 20°C at night). The experimental plants were 6-month-old *I. vomitoria* plants propagated from cuttings. Each experimental solution (extracts, H_2O fraction, EtOAc fraction, and t-BuOH fraction) was applied on three spots of the upper surface of each of the three randomly selected mature leaves per plant by pipet at the dosage of 10 µL each spot, respectively. Three untreated leaves per plant served as controls. The leaf surfaces of the experimental plants were checked daily for two weeks by a ×60 portable microscope linked to an iPhone™. Foliar Sprays: The experiment plant is the mature tree in the field where the fruits were collected from extraction in this example. Each experimental solution (extracts, H_2O fraction, EtOAc fraction, and t-BuOH fraction) was sprayed on each of the three randomly selected 30-cm long branches by spray bottle at the dosage of 10 mL, respectively. Three untreated stems with leaves served as controls. The leaf surfaces of the experimental plants were checked daily for two weeks by a ×60 portable microscope linked to an iPhone™.

Results: The t-BuOH fraction of the EtOH extracts of *I. vomitoria* fruits was found most active in the *I. vomitoria* leaf bioassays, following by the EtOAc fraction and then the whole extracts while the H_2O fraction not active. All leaf spots treated by 10 µL t-BuOH fraction turned brown within 24 h and whole leaves were dead within nine days (Supplementary Fig. S34). The foliar sprays of branch (leaves and stems) in the field showed a similar result. The terminal buds and shoots of the three branches sprayed with 10 mL of t-BuOH fraction each were all dead with some leaf injury within two weeks.

2.15. Elimination and Inhibition of Brazilian Pepper Tree (*Schinus terebinthifolius* Raddi) Seedlings by Its EtOH Extracts

Brazilian pepper tree (*Schinus terebinthifolius* Raddi) (Anacardiaceae) is an evergreen shrub or tree native to South America and was introduced to North America in the 1800s as an ornamental plant. Like *T. radicans* of the same family, this species may also cause dermatitis to people with sensitive skin and even respiratory problems during its bloom period. *Schinus terebinthifolius* has extensively infested landscapes in Florida and south Texas, USA and it produces a dense canopy that shades out other plants particularly native species.

General Experimental Procedures: Extracts Preparation: The fruits of *S. terebinthifolius* (200 g, in dry weight) and *T. sebifera* (260 g, in dry weight) were ground to coarse powders and

extracted two times with 70% EtOH at room temperature (each 2 L, 48 h). After evaporated under reduced pressure, 25.4 g EtOH extracts of *S. terebinthifolius* and 27 g EtOH extracts of *T. sebifera* (both in dry weight) were obtained, respectively. Each extracts were dissolved in nanopure H_2O and then was prepared as experimental solutions at the concentration of 10%, respectively. Foliar Sprays: The spray treatment experiment included 36 *S. terebinthifolius* seedlings (three weeks old) in pots with Miracle Grow Potting Mix soil in the greenhouse (30°C). There were six treatments. Controls: six seedlings were sprayed twice with a total 3 mL of H_2O each time; surfactant treatment: six seedlings were sprayed twice with a total 3 mL of H_2O with 7.5 uL Inlet and 3.75 uL Kinetin each time; *S. terebinthifolius* extracts treatment: six seedlings were sprayed twice with a total 3 mL of 10% *S. terebinthifolius* fruit extracts each time; *S. terebinthifolius* extracts with surfactants treatment: six seedlings were sprayed twice with a total 3 mL of 10% *S. terebinthifolius* fruit extracts with 7.5 uL Inlet and 3.75 uL Kinetin each time; *T. sebifera* extracts treatment: six seedlings were sprayed twice with total 3 mL of 10% *T. sebifera* fruit extracts each time; and *T. sebifera* extracts with surfactants treatment: six seedlings were sprayed twice with a total 3 mL of 10% *T. sebifera* extracts with 7.5 uL Inlet and 3.75 uL Kinetin each time. In addition, newly-spread leaves of six seedlings of each of the three native species in North America, namely poison ivy (*Toxicodendron radicans* (L.) Kuntze or *Rhus toxicodendron* L.) (family Anacardiaceae), sweetgum (*L. styraciflua*), and Shumard oak (*Q. shumardii*) were sprayed twice with a total 3 mL of 10% *S. terebinthifolius* fruit extracts each time, respectively. Plant growth and survival status were documented and photographed daily after the treatments.

Results: All *S. terebinthifolius* seedlings treated with *S. terebinthifolius* extracts or the extracts with surfactants had significant damages three days after the first treatments (Supplementary Fig. S35). Four weeks later, all six *S. terebinthifolius* seedlings were killed by the *S. terebinthifolius* extracts with surfactants while five seedlings were killed and one was significantly damaged with dead apical bud and young leaves by the *S. terebinthifolius* extract. In contrast, the *S. terebinthifolius* plants had no damages in the controls, surfactants, 10% EtOH extracts of *T. sebifera* fruits, or 10% EtOH extracts of *T. sebifera* fruits with surfactants treatment. None of *T. radicans*, *L. styraciflua*, or *Q. shumardii* seedlings had any damages by the *Toxicodendron radicans* extracts.

2.16. Elimination and Inhibition of Tree of Heaven (*Ailanthus altissima* (P. Mill.) Swingle) by Its EtOH Extracts

Tree of heaven (*Ailanthus altissima* (P. Mill.) Swingle) (Simaroubaceae) is native to China. It has been grown for traditional Chinese medicine and as a host plant for the ailanthus silkmoth, which is used for silk production. It is a serious threat to the native ecosystems in the introduced areas in North America and Europe. The rapidly growing species has become one of the most important invasive tree species in North America.

General Experimental Procedures: Extracts Preparation: The leaves and stems of *A. altissima* were collected from Nacogdoches, Texas, USA. The leaves and stems were dried in an oven at 65°C for 48 h. The dried leaf and stem samples (8.6 kg) were ground to a coarse powder and extracted two times for 48 h each with 95% EtOH (32 L and 24 L, respectively) at room temperature. The combined EtOH extracts were concentrated under reduced pressure and a total of 415 g extracts were obtained. 60 g the extracts were dissolved and suspended in bark oil EC Blue (UAP Distribution, Inc., Greeley, CO) and prepared as 300 mL experimental solution at the concentration of 20% leaf and stem extracts. The treatment experiments were conducted in the field of the plant matter collection site. Hack and Squirt Applications: nine plants of *A. altissima* in the field with diameter at breast height (DBH) ranging from 20-45 mm were selected for hack and squirt application. Three trees without any treatment served as controls. Three *A. altissima* trees were cut in the trunk using a hatchet and each was squirt with 5 mL of *A. altissima* EtOH extracts with a squirt bottle. Three trees had the hack and squirt application with bark oil only. Cut Stump Treatments: Nine *A. altissima* trees were cut down at about 19 cm above the ground. Three of them without treatment were used as the controls. Three *A. altissima* trees were treated with *A. altissima* extracts onto the cut stumps at the amount of 5 mL per stump. Another three were treated with bark oil only at the amount of 5 mL per stump. Basal Treatments: The basal bark application group had nine plants. Three without any treatment were used as the controls. Three were sprayed with *A. altissima* EtOH extracts on the lower 40 cm of the trunk at the amount of 5 mL per tree. Three were sprayed with bark oil only at the amount of 5 mL per tree. Plant status was evaluated and photographed every two weeks after the treatment.

Results: By the end of 4th week after the treatments, no coppice growth was seen from the *A. altissima* stumps treated with the *A. altissima* extracts in the cut stump experiments. At the same

time, about 57% of the leaves of all three *A. altissima* trees in the hack and squit treatment with *A. altissima* extracts had turned yellow or fallen in comparison with 50% in the trees treated with oil or 0% in the trees in the control group. Also at the same time, about 87% of the leaves of all three trees in the basal bark applications of *A. altissima* extracts had turned yellow or fallen in comparison with 10% in the trees treated with oil or 0% in the trees in the control group.

2.17. Elimination and Inhibition of Chinese Privet (*Ligustrum sinense* Lour.) by Its EtOH Extracts

Chinese privet (*Ligustrum sinense* Lour.) (Oleaceae) is a deciduous shrub native to China. It has invaded mesic forests throughout the Southeastern United States during the past century. It is estimated that Chinese privet now occupies over one million hectares of land in USA.

General Experimental Procedures: Extracts Preparation: The fruits of Chinese privet were collected from Nacogdoches, Texas, USA. The fruits were dried in an oven at 65°C for 48 h, separately. 400 g dried fruits were ground to a coarse powder and extracted two times for 48 h each with 95% EtOH (2 L and 1 L, respectively) at room temperature. The combined EtOH extracts were concentrated under reduced pressure and a total of 32 g Chinese privet extracts were obtained. 20 g extracts were dissolved and suspended in H_2O and prepared as 200 mL experimental solution at the concentration of 10% extracts with 0.25% Dyne-Amic (v:v) and 0.125% Kinetin (v:v). Cut Stump Treatments: The treatment experiments were conducted in the field of the plant matter collection site. 10 Chinese privet plants (2.5 to 3 m in height, approximately 5 cm in basal diameter) were cut 15 cm from the ground. A 3 cm long and 1 cm wide hole was made in the center of each stump. Five stumps served as the controls and each received 10 mL of tap water injection into the hole. Each of the other five stumps received 10 mL of extracts with surfactants injection. Two additional applications of the same amount were made on the second and 5th day. The regrowth of plants in the control or cut stump treatment was measured monthly for six months.

Results: All treated stumps had about 2 weeks delayed coppice growth than those in the control group by the end of the two months after the treatments. Four months later, stumps in the control group had 13.5 coppices with 283.5 cm in height at average while stumps in treatment had 3.5 coppices with 97.8 cm in height at average (Supplementary Fig. S36). Of the five treated stump, one had no coppice growth during the six months of observation.

2.18. Inhibition on Seed Germination of Red Kidney Bean (*Phaseolus vulgaris* L.) by Its EtOH Extracts

General Experimental Procedures: Extracts Preparation: 906 g dried red kidney bean (*Phaseolus vulgaris* L.) (Fabaceae) were ground to coarse powders and extracted two times for 48 h with 95% EtOH (4 L and 3 L, respectively) at room temperature. Extracts were evaporated under reduced pressure, and 7.1 g EtOH extracts were obtained. 7.1 g EtOH extracts were dissolved and suspended in nanopure H_2O and prepared as 140 mL experimental solution at the concentration of 5%. Then 20 mL of 5% EtOH extracts was diluted to 100 mL and prepared as 100 mL experimental solution at the concentration of 1%. The treatment experiments were conducted in the laboratory under room temperature. Extracts Yield of Experimental Plant Matter: Based on the plant matter weight and extraction rate (0.79%, in dry weight) of the above extraction method, it is estimated that the plant matter for soaking treatment (30 beans) could produce 0.13 g EtOH extracts. To further reveal the actual amount of the EtOH extracts contained in the plant matter, a more effective extraction of 30 beans (16.9 g) was performed using a ASE 2000 Accelerated Solvent Extractor (60 ^{o}C, 1500 psi, 30 min static time, 100% volume flush, 120 s purge, and 2 cycles). According to this extraction rate, 30 beans contain at least 0.9 g EtOH extracts. Soaking Treatments and Germination Tests: 270 beans were prepared and subjected to one of the three treatments for 4 days. (1) 30 beans were placed in a Petri dish with 30 mL of nanopure H_2O; (2) 30 beans were placed in a Petri dish with 30 mL of 1% EtOH extracts (0.3 g extracts); (3) 30 beans were placed in a Petri dish with 30 mL of 5% EtOH extracts (1.5 g extracts). Each experiment had three replications. The germination of bean was recorded every day throughout the experimental period.

Results: Germination of *P. vulgaris* beans was inhibited and delayed by the bean extracts. More than 70% of beans did not germinate and lost viability after soaked in a 5% solution of *P. vulgaris* extracts for 4 days (Supplementary Fig. S37).

2.19. Inhibition on Seed Germination of Peanut (*Arachis hypogaea* L.) by EtOH Extracts of Its Seeds

General Experimental Procedures: The experimental material and methods were described in the method section of "Induced Mutations in Peanut (*Arachis hypogaea* L.) by Its Extracts" in the text The seed germination was recorded weekly throughout the experimental period of four weeks. The data were analyzed by SPSS 13.0 for Windows (SPSS, Inc., Chicago, IL). ANOVA

with Duncan post-hoc analysis was used to compare the means of the control and treatment groups.

Results: By the end of four weeks after sowing in the pots, the *A. hypogaea* seeds without any treatment had 73.3% germination rate at average. The seed soaked in nanopure H_2O for a week had about 22.3% germination rate average. The seeds soaked in a 5% solution of *A. hypogaea* shell extracts or seed extracts had only 7.7% or no germination, respectively (Supplementary Fig. S38). The ungerminated seeds in these two treatments lost viability and never germinated in the next six months of observation.

2.20. Inhibition of Shumard Oak (*Quercus shumardii* Buckley) and Other Oak Species by EtOH Extracts of Shumard Oak Acorns

2.20.1. Inhibition on Acorn Germination of Shumard Oak (*Quercus shumardii* Buckley) and Nuttall Oak (*Q. texana* Buckley) by EtOH Extracts of Shumard Oak Acorns

General Experimental Procedures: Extracts Preparation: The acorns of shumard oak (*Quercus shumardii*) were collected from Nacogdoches, Texas, USA, in November 2014 and were dried in an oven at 65°C for 48 h. 1.2 kg dried acorn were ground to coarse powders and extracted two times for 48 h with 95% EtOH (4.8 L and 4 L, respectively) at room temperature. Extracts were evaporated under reduced pressure. 61 g EtOH extracts were obtained. 25 g extracts were dissolved and suspended in nanopure H_2O and prepared as 500 mL experimental solution at the concentration of 5% EtOH extracts of acorns of *Q. shumardii*. 45 mL of 5% EtOH extracts of acorns was diluted to 450 ml and prepared as 450 mL experimental solution at the concentration of 0.5% EtOH extracts of acorns. Extracts Yield of Experimental Acorns: Based on the acorn weight and extraction rate (5.09%, in dry weight) of the above extraction method, it is estimated that the plant matter for soaking treatment (30 *Q. shumardii* acorns) could produce 12.22 g EtOH extracts. To further reveal the actual amount of the EtOH extracts contained in the plant matter, a more effective extraction of small plant sample (24 g) was performed using a ASE 2000 Accelerated Solvent Extractor (60 ^{o}C, 1500 psi, 30 min static time, 100% volume flush, 120 s purge, and 2 cycles). According to this extraction rate, 30 *Q. shumardii* acorns contain at least 18.96 g EtOH extracts. Soaking Treatments: The treatment experiments were conducted in the laboratory. 270 acorns from each species of *Q. shumardii* and *Q. texana* were prepared and each group consisting of 30 acorns in a plastic conainer (14 × 15 cm, 0.68 L) were subjected to one of

the three soaking treatments for 48 h at room temperature: 150 mL of nanopure H_2O (as the controls), a 150 mL of 0.5% solution of EtOH extracts of *Q. shumardii* acorns (0.75 g acorn extracts), and a 150 mL of 5% solution of EtOH extracts of *Q. shumardii* acorns (7.5 g acorn extracts). Each treatment included three replicates. Germination Tests: The 30 soaked acorns in each replicate per treatment were placed in the germination box with moist sand. Acorns were considered germinated when the radical protruded through the pericarp. The number of germinated individuals and the length of the radical were recorded once every week throughout the experimental period of 11 weeks. The percentage of germination and the mean germination time were determined for each replicate. The acorns with the radical were planted in one gallon pots with Miracle Grow Potting Mix soil. The number of germinated seedlings and the height of the seedling were recorded once every week throughout the experimental period. Statistic Analysis: The data were analyzed by SPSS 13.0 for Windows (SPSS, Inc., Chicago, IL). ANOVA with Duncan post-hoc analysis was used to compare the means of the control and soaking treatment groups.

Results: The germination rates of both *Q. shumardii* and *Q. texana* acorns soaked in a 5% solution of EtOH extracts of *Q. shumardii* acorns were significantly decreased in comparison with those soaked in a 0.5% solution of EtOH extracts of *Q. shumardii* acorns or the control group. By the end of the eleventh week, about 45.7% of *Q. shumardii* acorns germinated after treatment with 5% *Q. shumardii* extracts in comparison with 63.3% germination rate of acorns soaked in water (Supplementary Fig. S39). At the same time, 50% of *Q. texana* acorns germinated after treatment with 5% *Q. shumardii* extracts in comparison with 75.7% germination rate of acorns soaked in water. The ungerminated acorns in all treatments lost viability and never germinated in the next six months of observation.

2.20.2. Phytotoxicity of EtOH Extracts of Shumard Oak (*Quercus shumardii* Buckley) Acorns on Shumard Oak, Nuttall Oak (*Q. texana* Buckley), and Swamp Chestnut Oak (*Q. michauxii* Nutt.)

General Experimental Procedures: Extracts Preparation: 1 g EtOH extracts of Shumard oak (*Quercus shumardii*) acorns (see 2.20.1) were prepared in 10 mL experimental solution with nanopure H_2O at the concentration of 10%. Bioassays: The experimental oaks were 3-week-old seedlings of *Q. shumardii*, Nuttall oak (*Q. texana*), and swamp chestnut oak (*Q. michauxii*) germinated from the seeds collected from Nacogdoches, Texas, USA, USA. The seedlings were

grown in pots with Miracle Grow Potting Mix soil in the greenhouse (30°C during the day time and 20°C at night). 10% EtOH extracts of *Q. shumardii* acorns were applied seprately by pipet on the upper or lower leaf surfaces of three plants of each species, respectively. For each plant species, three upper leaves and three lower leaves were treated separately. The leaf surfaces of the experimental plants were checked daily for two weeks by a ×60 portable microscope linked to an iPhone^{TM}.

Results: The leaves of all three testing oak species had significant tissues damaged by 10 µL 10% EtOH extracts of *Q. shumardii* acorns when applied on lower leaf surfaces. In *Q. michauxii*, application of 10 µL 10% EtOH extracts of *Q. shumardii* acorns on upper leaf surfaces also caused some tissue damages, but less serious than by lower leaf surface application. However, both *Q. texana* and *Q. michauxii* leaves had no been affected by applications of 10 µL 10% EtOH extracts of *Q. shumardii* on upper leaf surfaces.

2.21. Inhibition on Acorn Germination of Nuttall Oak (*Quercus texana* Buckley) by EtOH Extracts of Different Parts of Nuttall Oak Acorns

General Experimental Procedures: Extracts Preparation: The acorns of Nuttall oak (*Quercus texana*) were collected from Nacogdoches, Texas, USA, in November 2014. Some acorns were separated into the pericarp and the embryo parts. Three samples (whole acorns, pericarps, and embryos) were dried in an oven at 65°C for 48 h. The dried samples were ground and obtained 300 g whole acorns, 340 g pericarps, and 240 g embryos. Each sample was extracted two times for 48 h each with 95% EtOH (1,500 mL each time) at room temperature. The combined EtOH extracts were concentrated to give three extracts: 16.0 g whole acorn extracts, 15.2 g acorn pericarp extracts, and 15.1 g acorn embryo extracts under reduced pressure, respectively. The 15 g each extracts were dissolved in nanopure H_2O and prepared as 300 mL experimental solution at the concentration of 5% EtOH extracts of *Q. texana*. Extracts Yield of Experimental Plant Matter: Based on the plant matter weight and extraction rate (5.34% for whole acorns, 4.47% for acorn pericarps, and 6.30% for embryos, in dry weight) of the above extraction method, it is estimated that the plant matter for soaking treatment (15 *Q. texana* acorns) could produce 0.8 g EtOH extracts of whole acorns or 0.4 g EtOH extracts of acorn pericarps and 0.4 g EtOH extracts of acorn embryos. Soaking Treatments: The treatment experiments were conducted in the laboratory. 180 acorns and 180 embryos of *Q. texana* were prepared and each group consisting of 15 acorns or embryos in a plastic conainer (14 × 15 cm, 0.68 L) were subjected to one of the

four soaking treatments for 48 h at room temperature: 15 mL of nanopure H_2O (as the controls), 15 mL of 5% solution of EtOH extracts of *Q. texana* acorns (0.75 g whole acorn extracts), 15 mL of 5% solution of EtOH extracts of *Q. texana* acorn pericarps (0.75 g pericarp extracts), and a 15 mL of 5% solution of EtOH extracts of *Q. texana* acorn embryos (0.75 g embryo extracts). Each treatment included three replicates. Germination Tests: The soaked acorns or embryos in each treatment were sowed in the pots with Miracle Grow Potting Mix soil in the greenhouse. The number of germinated individuals and the height of the seedlings were recorded once every week throughout the experimental period. Statistic Analysis: The data were analyzed by SPSS 13.0 for Windows (SPSS, Inc., Chicago, IL). ANOVA with Duncan post-hoc analysis was used to compare the means of the control and treatment groups.

Results: By the end of 10 weeks of planting, the germination rates (73.3-91.1%) of the acorn embryos of *Q. texana* had no significant difference among the treatments of water, extracts of *Q. texana* whole acorns, and extracts of *Q. texana* acorn pericarps but all were significantly higher than the embryos treated with extracts of *Q. texana* acorn embryos (48.9%) or whole acorns of *Q. texana* in any treartments (48.9-57.8%) (Supplementary Fig. S40). This shows that the endocidal ingredients in acorns may be primarily stored in embryos rather than pericarps. The ungerminated acorns or embryos in all treatments lost viability and never germinated in the next six months of observation. The height growth of seedlings in different treatments exibited a similar pattern (Supplementary Fig. S40).

2.22. Inhibition on Acorn Germination of Swatooth Oak (*Quercus acutissima* Carruth) by Its Acorn EtOH Extracts

General Experimental Procedures: Extracts Preparation: The acorns of sawtooth oak (*Quercus acutissima* Carruth) (350 g in dry weight) were ground to a coarse powder and extracted two times for 48 h each with 95% EtOH (1 L each time) at room temperature. The combined extracts were concentrated to yield 13 g under reduced pressure. The 10 g extracts were dissolved in nanopure H_2O and prepared as 200 mL experimental solution at the concentration of 5% EtOH extracts of acorns. Extracts Yield of Experimental Acorns: Based on the acorn weight and extraction rate (3.72%, in dry weight) of the above extraction method, it is estimated that the plant matter for soaking treatment (25 *Q. acutissima* acorns) could produce 5.8 g EtOH extracts. To further reveal the actual amount of the EtOH extracts contained in the plant matter, a more effective extraction of small plant sample (20 g) was performed using a ASE 2000 Accelerated

Solvent Extractor (60 ^{o}C, 1500 psi, 30 min static time, 100% volume flush, 120 s purge, and 2 cycles). According to this extraction rate, 25 *Q. acutissima* acorns contain at least 11.69 g EtOH extracts. Soaking Treatments: The treatment experiments were conducted in the laboratory and greenhouse. 75 healthy acorns of *Q. acutissima* were selected with 25 in a plastic container (14 × 15 cm, 0.68 L). The acorns were subjected to one of the three treatments for two weeks at room temperature: controls (without any treatment), soaked in 200 mL of nanopure H_2O, and soaked in a 200 mL of 5% solution of *Q. acutissima* acorn EtOH extracts (10 g extracts). Germination Tests: The experimental acorns were planted in 1-gallon pots with Miracle Grow Potting Mix soil with three acorns each. The number of germinated individuals was recorded once every week throughout the experimental period.

Results: 24 seedlings had germinated from the 25 corns without any soaking treatment within eight weeks. By the same time, 16 seedlings germinated from the 25 acorns under the water soaking treatment, and only one seedling germinated from the 25 acorns soaked in a 5% solution of EtOH extracts of *Q. acutissima* acorns. The ungerminated acorns in this treatment lost viability and never germinated in the next three months of observation.

2.23. Phytotoxicity of EtOH of Water Lettuce (*Pistia stratiotes* L.) on Water Lettuce

General Experimental Procedures: Extracts Preparation: The whole plants of water lettuce (*Pistia stratiotes* L.) (Araceae) were collected from Texas, USA. The plants were dried in an oven at 50°C for 48 h. 20 g dried plant matter was ground to a coarse powder and extracted three times for 48 h each with 95% EtOH (1 L each time) at room temperature. The combined EtOH extracts were concentrated under reduced pressure and the total 160 mg extracts were obtained. 50 mg extracts were dissolved and suspended in nanopure H_2O and prepared as 500 mL experimental solutions at the concentration of 10% (v:v). Bioassays: The experiment was conducted in the greenhouse (30°C during the day time and 20°C at night). The experimental solution was applied on upper and lower surfaces of three 6-month-old plants (three spots each side per leaf, three leaves per plant) by pipet at the dosage of 10 µL each spot, respectively. Three untreated leaves per plant served as the controls. The leaf surfaces of the experimental plants were checked daily for two weeks by a ×60 portable microscope linked to an iPhone™.

Results: The leaves of *P. stratiotes* had response to the treatment of *P. stratiotes* extracts, particularly applications on lower leaf surface within 24 h. By the three days, the leaf spots

treated by *P. stratiotes* extracts on both sides turned into brown in color and almost all treated leaves died within two weeks (Supplementary Fig. S41).

2.24. Inhibition on Clove Germination of Garlic (*Allium sativum* L.) by Its Fresh Clove Juice

General Experimental Procedures: Extracts Preparation: The total of 105 cloves of garlic (*Allium sativum* L.) (Amaryllidaceae) were carefully collected and subjected to 5 treatments. Each of the five treatments included three replicates with 5 or 10 cloves in each replication. Soaking Treatment and Germination Tests: All experiments were conducted under room temperature (1) 5 cloves were placed in a Petri dish without water; (2) 5 cloves were placed in a Petri dish and grown in 30 mL of nanopure H_2O; (3) 10 cloves were placed in a Petri dish and grown in 30 mL of nanopure H_2O; (4) 5 cloves and 5 shredded cloves with 30 mL of nanopure H_2O were added to a Petri dish with three replicates; (5) 5 cloves and 5 shredded beans of *P. vulgaris* with 30 mL of nanopure H_2O were added to a Petri dish. The length of roots and shoots were measured daily for 12 days. Statistic Analysis: The data were analyzed by SPSS 13.0 for Windows (SPSS, Inc., Chicago, IL). ANOVA with Duncan post-hoc analysis was used to compare the means of the control and treatment groups.

Results: Both shoot and root development of *A. sativum* was totally inhibited by shredded *A. sativum* cloves (Supplementary Figs. S42 and S43). The shredded beans of *P. vulgaris* had less significant impact on *A. sativum* growth.

2.25. Inhibition of Water Hyacinth (*Eichhornia crassipes* (Mart.) Solms) by Its EtOH Extracts

Water hyacinth (*Eichhornia crassipes* (Mart.) Solms) (Pontederiaceae) is an aquatic species native to South America. It has become one of most prevalent invasive aquatic species in North America, Asia, Africa, Australia, and New Zealand. This free-floating plant commonly forms dense, interlocking mats due to its rapid reproductive rate and complex root structure. It reproduces both sexually and asexually. The species has caused significant ecological and socio-economic effects in the world.

General Experimental Procedures: Extracts Preparation: The whole plants of water hyacinth were collected from Lake Sam Rayburn, Texas, USA. The plants were dried in an oven at 65°C for 48 h. 2.4 kg of dried plant matter was ground to a coarse powder and extracted two times for

48 h each with 95% EtOH (15 L and 12 L, respectively) at room temperature. The combined EtOH extracts were concentrated under reduced pressure and a total of 104 g extracts were obtained. 60 g extracts were dissolved and suspended in nanopure H_2O and prepared as 600 mL experimental solutions at the concentration of 10% extracts with 0.25% Dyne-Amic (v:v) and 0.125% Kinetin (v:v). Foliar Sprays: Each of the six containers had three healthy mature plants of water hyacinth in 25 L of tap water in the greenhouse (30°C during the day and 20°C at night). Three containers served as the controls without any treatment, and the other three received foliar application of 10% EtOH extracts of water hyacinth with surfactants in the amount of 30 mL each. For these three treatment containers, each had two additional treatments in the same amount on the second and third weeks. Foliar application of 90 mL of 10% EtOH extracts of water hyacinth with surfactants was also made on *S. molesta* (at the tertiary stage) and seedlings of sweetgum and swamp chestnut oak bald cypress, and loblolly pine. The plant status was documented and photographed daily after the treatment.

Results: During the experiments, water hyacinth plants in the control group grew well. After treatment by EtOH extracts of water hyacinth with surfactants, water hyacinth plants had significant injury (Supplementary Fig. S44). By the end of the third week, most leaves and spongy and bulbous stalks of water hyacinth were dead. The extracts did not show inhibition activity on any other test species.

2.26. Inhibition of Sorghum (*Sorghum bicolor* (L.) Moench) by Its EtOH Extracts

2.26.1. Inhibition on Seed Germination of Sorghum (*Sorghum bicolor* (L.) Moench) by Its Seed EtOH Extracts

General Experimental Procedures: Extracts Preparation: The 2 kg of seeds of sorghum (*Sorghum bicolor* (L.) Moench) (Poaceae) were purchased from Wicked Whitetails (Elko, MN, USA). 1 kg seeds were dried in an oven at 65°C for 48 h. The dried plant matter was ground to a coarse powder and extracted two times for 48 h each with 95% EtOH (6 L and 12 L, respectively) at room temperature. The combined EtOH extracts were concentrated under reduced pressure and a total of 52 g extracts were obtained. 3 g extracts were dissolved and suspended in nanopure H_2O and prepared as 60 mL experimental solutions at the concentration of 5% extracts. 6 g extracts were dissolved and suspended in nanopure H_2O and prepared as 60 mL experimental solutions at the concentration of 10% extracts. Extracts Yield of Experimental

Seeds: Based on the seed weight and extraction rate (5.2%, in fresh weight) of the above extraction method, it is estimated that the plant matter for soaking treatment (300 *S. bicolor* seeds) could produce 0.36 g EtOH extracts. To further reveal the actual amount of the EtOH extracts contained in the plant matter, a more effective extraction of small plant sample (6.9 g) was performed using a ASE 2000 Accelerated Solvent Extractor (60 ^{o}C, 1500 psi, 30 min static time, 100% volume flush, 120 s purge, and 2 cycles). According to this extraction rate, 300 *S. bicolor* seeds contain at least 0.4 g EtOH extracts. Soaking Treatments and Germination Tests: All experiments were conducted under room temperature. 2,700 sound seeds were selected and placed in nine Petri dishes with 300 seeds each. The seeds in each Petri dish were subjected to one of the following three treatments with three replications per treatment: soaked with 20 mL of nanopure H_2O (controls), soaked with a 20 mL of 5% solution of EtOH extracts of *S. bicolor* seeds (1 g extracts), and soaked with a 20 mL of 10% solution of EtOH extracts of *S. bicolor* seeds (2 g extracts). The seed germination was surveyed 72 h after the treatment. The data were analyzed by SPSS 13.0 for Windows (SPSS, Inc., Chicago, IL). ANOVA with Duncan post-hoc analysis was used to compare the means of the control and soaking treatment groups.

Results: The germination rates of *S. bicolor* seeds significantly decreased with the increasing concentration of the EtOH extracts of *S. bicolor* seeds. After 72 h of soaking treatmeant, 93.7% (±1.35), 56.1% (±2.7), and 18.0% (±3.48%) of seeds had germinated in the treatment of water, 5% EtOH extracts of *S. bicolor* seeds, and 10% EtOH extracts of *S. bicolor* seeds, respectively.

2.26.2. Phytotoxicity of EtOH Extracts of of Sorghum (*Sorghum bicolor* (L.) Moench) on Sorghum

General Experimental Procedures: Extracts Preparation: The seeds of sorghum (*Sorghum bicolor*) were collected from Nacogdoches, Texas, USA. The seeds were sowed in the pots in the greenhouse (30°C during the day time and 20°C at night). The whole plants of six three-month-old seedlings (before flowering) were harvested and dried in an oven at 65°C for 48 h. 80 g dried ground matter of *S. bicolor* was extracted 3 times for 48 h each with 95% EtOH (2.5 L each time) at room temperature. The combined EtOH extracts were concentrated under reduced pressure and the total 4.4 g extracts were obtained. 1 g extracts were dissolved and suspended in nanopure H_2O and prepared as 20 mL experimental solutions at the concentration of 5% extracts. Bioassays: The four-month-old *S. bicolor* seedlings were tested for phytotoxicity assays in the greenhouse. Mature plants of Johnsongrass (*Sorghum halepense* (L.) Pers.), *A. donax* and *P.*

aurea, three other species of Poaceae were also tested in the field. The experimental solution of 5% *S. bicolor* extracts was applied on upper and lower surfaces (three spots each side per leaf, three leaves per plant) of three plants each species by pipet at the dosage of 10 µL each spot, respectively. Three untreated leaves per plant served as the controls. The leaf surfaces of the experimental plants were checked daily for two weeks by a ×60 portable microscope linked to an iPhone^{TM}.

Results: By the end of the experiment, each of the testing *S. bicolor* leaf spot and its surrounding area (2-3 times of the spot in size each) were significantly damaged by 5% *S. bicolor* extracts on either upper or low surface and about 30% of the test leaf spots of *S. halepense* particularly by the application on lower leaf surface had somewhat damages. About 25.9% of *A. donax* leaf spots treated with 5% *S. bicolor* extracts on upper leaf surface had somewhat damages but primarily limited to the treating spot. The *S. bicolor* extracts had no effects on the leaves of *S. halepense* when applied on upper leaf surface, *A. donax* on lower leaf surface, and *P. aurea* on either upper or lower surface.

2.27. Inhibition of Johnsongrass (*Sorghum halepense* (L.) Pers.) by Its EtOH Extracts

Johnsongrass (*Sorghum halepense*) is native to the Mediterranean region, and has been introduced to all continents except Antarctica. Through its rapid reproduction by rhizomes and seeds, the grass can quickly invade crop fields, pastures, disturbed sites, forest edges, and along stream banks. The species was recognized as one of the six most damaging weeds in USA by the turn of the 20th Century, and was the first weed targeted by the USDA for research on control methods. Recently, glyphosate-resistant Johnsongrass has evolved.

General Experimental Procedures: Extracts Preparation: Extracts Preparation: (1) Extracts for Foliar Treatment: The whole plants of *S. halepense* were collected from Nacogdoches, Texas. The plants were dried in an oven at 65°C for 48 h. 3,600 g dried plant matter was ground to a coarse powder and extracted two times for 48 h each with 95% EtOH (20 L and 12 L, respectively) at room temperature. The combined EtOH extracts were concentrated under reduced pressure and the total 110 g extracts were obtained. 30 g extracts were dissolved and suspended in nanopure H_2O and prepared as 100 mL experimental solutions at the concentration of 10% with 1% Tergitol 15-S-9 (Sigma-Aldrich Co., St. Louis, MO, USA) (v:v). (2) Extracts for Bioassays: 800 g dried ground matter of *S. halepense* was extracted 3 times for 48 h each

with 95% EtOH (3 L each time) at room temperature. The combined EtOH extracts were concentrated under reduced pressure and the total 46 g extracts were obtained. 1 g extracts were dissolved and suspended in nanopure H_2O and prepared as 20 mL experimental solutions at the concentration of 5% extracts. Foliar Sprays: Three *S. halepense* plants were sprayed with 10 mL of the experimental solution twice each. The plant status was documented and photographed weekly after the treatment. Bioassays: The phytotoxicity experiment for four species of Poaceae was conducted in the greenhouse (30°C during the day time and 20°C at night) or field. Four-month-old *S. bicolor* seedlings in the greenhouse and mature plants of *A. donax*, *P. aurea*, *S. bicolor*, and *S. halepense* in the field were selected for the bioassays. The experimental solution of 5% *S. halepense* extracts was applied on upper and lower surfaces (three spots each side per leaf, three leaves per plant) of three plants each species by pipet at the dosage of 10 µL each spot, respectively. Three untreated leaves per plant served as the controls. The leaf surfaces of the experimental plants were checked daily for two weeks by a ×60 portable microscope linked to an iPhone™.

Results: The *S. halepense* plants showed significant injury to the leaves within two weeks after the foliar treatment with *S. halepense* extracts and no new growth was observed during the six weeks of investigation (Supplementary Fig. S45). In bioassays, *S. halepense* extracts showed toxicity on *S. halepense* and *S. bicolor* within 48 h. All leaf spots and their surrounding areas (usually 5 times or more of the spot in size each) were significantly damaged by the application of *S. halepense* extracts. *S. halepense* extracts did not show any effects on either *A. donax* or *P. aurea.*

2.28. Inhibition of Giant Reed (*Arundo donax* L.) by Its EtOH Extracts

General Experimental Procedures: Extracts Preparation: The rhizomes of giant reed (*Arundo donax* L.) (Poaceae) was collected from Nacogdoches, Texas, USA. The plant matter were dried in an oven at 65°C for 48 h. 3.5 kg dried plant matter was ground to a coarse powder and extracted three times for 48 h each with 95% EtOH (10 L each time) at room temperature. The combined extracts were concentrated to yield 53 g under reduced pressure. 1.25, 2.5, and 5 g *A. donax* extracts were dissolved and suspended in nanopure H_2O separately and prepared as 100 mL experimental solutions at the concentration of 1.25, 2.5, and 5% (v:v), respectively. 15 g *A. donax* extracts were dissolved and suspended in nanopure H_2O and prepared as 150 mL

experimental solutions at the concentration of 10% (v:v). Foliar Sprays: Each of the three plants of *A. donax* in the field were sprayed with 15 mL of 10% *A. donax* EtOH extracts and three plants had no any treatment. The plant status was documented and photographed daily after the treatment. Bioassays: 15 plants of *A. donax* in the field were selected for the experiment. Each concentration of 1.25, 2.5, 5, and 10% *A. donax* EtOH was applied on three spots of both upper and lower surfaces of each of the three randomly selected mature leaves per plant by pipet at the dosage of 10 µL each spot, respectively. Three untreated plants served as the controls. The leaf surfaces of the experimental plants were checked daily for two weeks by a ×60 portable microscope linked to an iPhone™.

Results: Leaves of *A. donax* treated with *A. donax* extracts at 2.5% or above concentrations showed some damage within 24 h of treatment in both foliar spray and bioasays of intact plants. By the of the two week experiment, the tissues in each leaf spot and its surrounding area (several times of the spot in size each) treated by all concentrations of *A. donax* extracts on either upper or low surface were totally killed.

2.29. Inhibition of Golden Bamboo (*Phyllostachys aurea* Carr. ex A. & C. Rivière) by Its EtOH Extracts

General Experimental Procedures: Extracts Preparation: The arial part of plants of golden bamboo (*Phyllostachys aurea* Rivière & C.Rivière) (Poaceae) was collected from Nacogdoches, Texas, USA. The plant matter were dried in an oven at 65°C for 48 h. 800 g dried plant matter was ground to a coarse powder and extracted two times for 48 h each with 95% EtOH (8 L each time) at room temperature. The combined EtOH extracts were concentrated under reduced pressure and the total 25.6 g extracts were obtained. EtOH extracts of giant reed (*A. donax*) were from the experiment of 2.28. 15 g *P. aurea* and *A. donax* extracts were separately dissolved and suspended in nanopure H_2O and prepared as 150 mL experimental solutions at the concentration of 5% (v:v), respectively. Cut Stump (Culm) Treatments: Nine *P. aurea* plants with about 5 cm in culm diameter were cut down at about 100 cm above the ground. Three culms without treatment were used as the controls. Three culms were treated with 10% *A. donax* EtOH extracts per onto the cut culms at the amount of 5 mL per culm. The rest three culms were treated with 10% *P. aurea* EtOH extracts per onto the cut culms at the amount of 5 mL per culm. The plant status was evaluated and photographed weekly after the treatments.

Results: The culms in both control and *A. donax* extracts treatment groups had no any observable

changes a week after the treatments. By the same time, all three culms treated with *P. aurea* EtOH extracts had significant damages. The tissues in the first nodes from the culm cut and adjacent 2-3 cm areas in this treatment turned from normal green into brown and soon died (Supplementary Fig. S46).

2.30. Elimination and Inhibition of Proliferating Bulrush (*Isolepis prolifera* (Rottb.) R. Br.) by Its EtOH Extracts

General Experimental Procedures: Extracts Preparation: The whole plants of proliferating bulrush (*Isolepis prolifera* (Rottb.) R. Br.) (Cyperaceae) were collected from Texas, USA. The plants were dried in an oven at 65°C for 48 h. 12 g dried plant matter was ground to a coarse powder and extracted two times for 48 h each with 95% EtOH (150 mL each time) at room temperature. The combined EtOH extracts were concentrated under reduced pressure and the total 0.72 g extracts were obtained. 0.7 g extracts were dissolved and suspended in nanopure H_2O and prepared as 14 mL experimental solutions at the concentration of 5% (v:v). Foliar Sprays: One plot (35 × 15 cm) of plants of *I. prolifera* was sprayed with 14 mL of the extracts by foliar spray. The plant status was documented and photographed weekly after the treatment.

Results: The terminal shoots of all testing plants of *I. prolifera* turned into brown and dead within two weeks of the treatment with *I. prolifera* extracts.

2.31. Elimination and Inhibition of the Red Important Fire Ant (*Solenopsis invicta* Buren) by Its Extracts and Formic and Acetic Acids

Since its introduction from its native South American range in the 1930s, the red imported fire ant (*Solenopsis invicta* Buren) (family Formicidae) has rapidly widespread throughout the southern USA. It has also recently invaded other regions of the world, including the Caribbean, Mexico, Australia, New Zealand, Malaysia, Singapore, and China[4-7]. Unlike other insects in Hymenoptera, the red imported fire ant contains a small fraction of proteins in its venom. About 95% of the fire ant venom consists of alkaloids (primarily 2-methyl-6-alkyl or alkenyl piperidines), which are responsible for the immediate hive formation and the development of the sterile pustule at the sting site. The pest detrimentally impacts human health, livestock, wildlife, crops, machinery, and electrical equipment. The estimated cost of control, medical treatment, and damage to property by fire ant in the USA alone is more than $6 billion annually[5]. An effective measure for control is needed for both ecological and economic reasons.

General Experimental Procedures: <u>Preparation of *S. invicta* exctracts</u>: (1) Acetone extract: 97 g (in fresh weight) of the red imported fire ant workers were extracted two times with acetone at root temperature (each 250 mL, 24 h). After evaporated under reduced pressure, the combined extractions yielded 2.13 g (in dry weight) acetone extract. (2) EtOH extract: 110 g (in fresh weight) of the whole worker bodies of *S. invicta* were extracted two times with 95% EtOH at room temperature (each 250 mL, 24 h). After evaporated under reduced pressure, the combined extractions yielded 2.72 g (in dry weight) EtOH extract. Both extracts were dissolved in nanopure H_2O and then prepared as experimental solutions at the concentration of 0.1%, 1%, 5%, and 10%, respectively. <u>Preparation of the formic and acetic acids</u>: Formic acid (88%, Sigma Aldrich©) and acetic acid (9.7, ACS reagent grade, VWR International LLC) were prepared as solutions at the concentration of 0.1%, 1%, and 5% immediately before the bioassay experiments. <u>Detection of formic acid by NMR analysis</u>: Approximately 100 *S. invicta* workers were anesthetized by CO_2 and dissolved in 1 mL deuterated $CHCl_3$ (chroroform). The mixture was put under ultrasound for 30 min extraction. The ants were then removed and the extracts were dried with anhydrous magnesium sulfate. 500 µL of the extracts were transferred to NMR tube for detection. ^{1}H-NMR experiments were performed on a JEOL ECS 400 spectrometer, with spectroscopic data referenced to the solvent used. According to standard formic acid ^{1}H-NMR spectrum, the unique singlet of the aldehyde proton should appear at δ_H 8.02. <u>Contact Toxicity Assays</u>: (1) Impacts of acetone and EtOH extracts of *S. invicta* on *S. invicta*: the experiment includes nine treatments and each treatment had the total 150 workers of *S. invicta* with 50 ants in each of the three 100 mL beakers (as three replications). The ants in each beaker were topically sprayed with a total 1 mL of 0.1%, 1%, 5%, or 10% solutions of either acetone or EtOH extract, respectively. The control group was sprayed with 1 mL of nanopure H_2O. The beakers in all treatments were covered by cloth to prevent the escape of *S. invicta*. The surviving number of *S. invicta* in each treatment was counted by 1 h interval for 7 h. (2) Impacts of formic and acetic acids on *S. invicta*: the experiment had seven treatments and each treatment had the total 150 workers of *S. invicta* with 50 ants in each of the three 100 mL beakers (as three replications). The ants in each beaker were topically sprayed with a total 1 mL of 0.1%, 1%, or 5% solutions of either formic or acetic acids, respectively. The control group was sprayed with 1 mL nanopure H_2O. The beakers in all treatments were covered by cloth to prevent the escape of *S. invicta*. The surviving number of *S. invicta* in each treatment was counted by 1 h interval for 7

h. For extracts and acids, LD_{50} (the dose required to kill half of the exposed *S. invicta*) and LD_{90} (the dose required to kill 90% of the exposed *S. invicta*) were calculated by the PROBIT procedure of SPSS 13.0 for Windows. (3) Impacts of combined application of EtOH extracts and formic acid on *S. invicta*: The experiment had the total 720 workers of *S. invicta* with 30 ants in each of the 24 Petri Dishes (85 mm in diameter). Controls: the ants in three dishes had no treatment; Water treatment: the ants in each of the three dishes were in contact with 1 mL of nanopure H_2O for 10 sec; 2.5% EtOH extracts treatment: the ants in each of the three dishes were in contacted with 1 mL of 2.5% EtOH extracts for 10 sec; 2.5% formic acid treatment: the ants in each of the three dishes were in contacted with 1 mL of 2.5% formic acid for 10 sec; mixture of EtOH extracts (2.5%) and formic acid (2.5%) treatment: the ants in each of the three dishes were in contacted with 1 mL of a mixture of EtOH extracts and formic acid (each had 2.5% in concentration in the mixture) for 10 sec; 5% EtOH extract treatment: the ants in each of the three dishes were in contacted with 1 mL of 5% EtOH extracts for 10 sec; 5% formic acid treatment: the ants in each of the three dishes were in contacted with 1 mL of 5% formic acid for 10 sec; and mixture of EtOH extracts (5%) and formic acid (5%) treatment: the ants in each of the three dishes were in contacted with 1 mL of a mixture of EtOH extracts and formic acid (each had 5% in concentration in the mixture) for 10 sec. The surviving number of *S. invicta* in each treatment was counted by 15 min interval for 90 min. (4) Impacts of combined application of EtOH extracts and formic acid on the injured fire ants: The 450 workers of the red imported fire ants were placed in 15 Petri Dishes (85 mm in diameter) with 30 in each dish. The ants were anesthetized by CO_2 and then each ant was injured in its gaster by pin. Controls: the ants in three dishes had no treatment; Water treatment: the ants in each of the three dishes were in contact with 1 mL of nanopure H_2O for 10 sec; 1.25% EtOH extract treatment: the ants in each of the thee dishes were in contacted with 1 mL of 1.25% EtOH extracts for 10 sec; 1.25% formic acid treatment: the ants in each of the thee dishes were in contact with 1 mL of 1.25% formic acid for 10 sec; and mixture of EtOH extracts (1.25%) and formic acid (1.25%) treatment: the ants in each of the three dishes were in contacted with 1 mL of a mixture of EtOH extracts and formic acid (each had 1.25% in concentration in the mixture) for 10 sec. The surviving number of *S. invicta* in each treatment was counted by 15 min interval for 90 min. (5) Impacts of EtOH extracts of *S. invicta* on subterranean termite (*Reticulitermes flavipes* (Kollar)) (family Rhinotermitidae): the experiment included five treatments and each treatment had 150

subterranean termites with 20 workers, 10 soldiers and 5 winged reproductive ants in each of the three 100 mL beakers (as three replications). The termites in each beaker were topically sprayed with a total 1 mL of 0.1%, 1%, 5%, or 10% solutions of EtOH extract, respectively. The control group was sprayed with 1 mL of pure H_2O. The beakers in all treatments were covered by cloth to prevent the escape of *S. invicta*. The surviving number of *S. invicta* in each treatment was counted by 1 h interval for 7 h. Fumigation Toxicity Assays of Formic Acid: The experiment included approximately 300 workers of *S. invicta* in each container with the filter paper treated with 10 mL of 0.1%, 1%, or 5% formic acid, respectively. The surviving number of *S. invicta* in each treatment was counted by 1 h interval for 2 h. LD_{50} (the dose required to kill half of the exposed *S. invicta*) and LD_{90} (the dose required to kill 90% of the exposed *S. invicta*) were calculated by the PROBIT procedure of SPSS 13.0 for Windows. The data were analyzed by SPSS 13.0 for Windows (SPSS, Inc., Chicago, IL). ANOVA with Duncan post-hoc analysis was used to compare the means of the control and treatment groups.

Results: The topical applications of both the extracts of *S. invicta* and the organic acids inhibited *S. invicta*. Both formic acid and acetic acid were not detected in chloroform, acetone, or EtOH extracts according to the NMR analysis. The EtOH extracts showed more significant toxicity against *S. invicta* than the acetone extract. During the 7 h contact toxicity bioassays, an average of approximately 60%, 70%, or 80% of *S. invicta* in contact with 1%, 5%, or 10% EtOH extracts were dead, respectively (Supplementary Fig. S47). In contact toxicity bioassays, LD_{50} and LD_{90} of formic acid, acetic acid, EtOH extract, and acetone extracts in 7 h for *S. invicta* was 1.9% and 5.09%, 6.42% and 9.96%, 1.81% and 15.58%, and 18.67% and 34.18%, respectively. Formic acid had a more significant impact on survival of *S. invicta* than acetic acid. During the 90 min of contact toxicity bioassay, the average mortality with 2.5% and 5% formic acid was more than 70% or 100%, respectively (Supplementary Fig. S48). In fumigation bioassays, LD_{50} and LD_{90} of formic acid for *S. invicta* in 2 h was 0.5% and 0.9%, respectively. The combined application of EtOH extracts of *S. invicta* and formic acid had more significant effects on *S. invicta* survival than use of either EtOH extracts or formic acid alone (Supplementary Figs. S48 and S49). However, the EtOH extracts of *S. invicta* had no impacts on subterranean termite.

2.32. Elimination and Inhibition of the Subterranean Termite (*Reticulitermes* flavipes (Kollar)) by Formic Acid

Eastern subterranean termite (*Reticulitermes flavipes* (Kollar)) (family Rhinotermitidae) is the most common termite found in North America. This native termite is one of the most economically important wood destroying pests in the USA and it causes billions of dollars in home damage each year. Currently, termite management includes reducing the potential for termite infestation, preventing entry, and chemical control.

General Experimental Procedures: Preparation of Experimental Agent*:* Formic acid (90.4%, certified ACS reagent grade, Fisher Scientific Company, Fair Lawn, NJ, USA) was prepared as solutions at the concentration of 0.1%, 1%, and 5% with nanopure H_2O immediately before the bioassay experiments. Detection of formic acid by NMR analysis: Approximately 100 termites were anesthetized by CO_2 and dissolved in 1 mL of deuterated $CHCl_3$ (chroroform). The mixture was put under ultrasound for 30 min extraction. The termites were then removed and the extracts were dried with anhydrous magnesium sulfate. 500 µL of the extracts were transferred to NMR tube for detection. ^{1}H-NMR experiments were performed on a JEOL ECS 400 spectrometer, with spectroscopic data referenced to the solvent used. According to standard formic acid ^{1}H-NMR spectrum, the unique singlet of the aldehyde proton should appear at δ_H 8.02. Contact Toxicity Assays: The experiment included five treatments and each treatment had total 105 termites including 20 workers, 10 soldiers and 5 winged reproductives (alates) in each of the three 100 mL beakers (as three replications). The termites in each beaker were topically sprayed with a total 1 mL of 0.1%, 1%, 5%, or 10% solutions of formic acid, respectively. The control group was sprayed with 1 mL of pure H_2O. The beakers in all treatments were covered by cloth to prevent the escape of termites. The surviving number of the termites in each treatment was counted by 1 h interval for 7 h. The data were analyzed by SPSS 13.0 for Windows (SPSS, Inc., Chicago, IL). ANOVA with Duncan post-hoc analysis was used to compare the means of the control and treatment groups.

Results: The NMR analysis indicated that *R. flavipes* contained significant amount of formic acid (Supplementary Fig. S50). Formic acid at higher concentrations (5 or 10%) killed all termites (including workers, soldiers, and reproductive) during the first hour of contact experiment. During the 7 h of contact toxicity bioassays, formic acid at 1% concentration killed more than 40% of the termites (Supplementary Fig. S51). However, formic acid at 0.1% concentration had

no impact on the termites. In the contact toxicity bioassays, LD_{50} and LD_{90} of formic acid in 7 h for the termite was 4.71% and 9.08%, respectively.

2.33. Elimination and Inhibition of American Grasshopper (*Schistocerca americana* Drury) by its EtOH Extracts

American grasshopper or American bird grasshopper (*Schistocerca americana* Drury) (Acrididae) is native to the Eastern USA, Mexico, and the Bahamas. The grasshopper can completely defoliate plants and can cause serious damage to crops or ornamental plants. There are occasional, localized outbreaks of this grasshopper; it is often referred to as a locust. It is considered to be the most destructive grasshopper in Florida. It is recommended to apply insecticides before the grasshoppers are adults because it is easier to kill the nymphs.

General Experimental Procedures: Extracts Preparation: Adults of *S. americana* were collected from Nacogdoches, Texas, USA. The whole insects were frozen for 6 h and then were dried in an oven at 65°C for 48 h. The dried insects (64 g) were ground to a coarse powder and extracted two times for 48 h each with 95% EtOH (0.7 L and 0.5 L, respectively) at room temperature. The combined EtOH extracts were concentrated under reduced pressure and a total of 3.5 g *S. americana* extracts were obtained. 2.25 g extracts were dissolved and suspended in nanopure H_2O and prepared as 22.5 mL experimental solution at the concentration of 10% extracts. Bioassays: The experiment included 60 living grasshopper adults, cultured and treated in plastic containers (34 × 27 cm) in the laboratory at room temperature. 30 *S. americana* served as the controls with 10 insects in each of the three containers and the insects in each control container were sprayed with 7.5 mL of H_2O only. 30 *S. americana* with 10 insects in each of the three containers were sprayed with 7.5 mL of 10% EtOH extracts. In both control and spray treatment containers, insects were fed with lettuce purchased from the local grocery store. Survival status was documented and photographed daily after the treatment.

Results: All 30 grasshoppers in the control group survived for seven days. In all three treatment replications, however, all *S. americana* quickly responded to 10% EtOH *S. americana* extracts. The insects became inactive soon after the treatments and all were dead within 24 h (Supplementary Fig. S52).

2.34. Elimination and Inhibition of Mealworm (*Tenebrio molitor* L.) and Superworm (*Zophobas morio* Fabricius) Larvae by Their EtOH Extracts

Tenebrio molitor L. and *Zophobas morio* Fabricius are members of darkling beetles family (Tenebrionidae). Their larvae are known as mealworms and superworms, respectively. *Tenebrio molitor* is originated from Europe and *Z. morio* is native tropical regions of Central and South America. Both are invasive pests in most regions of the world.

General Experimental Procedures: The experimental larvae of both *T. molitor* and *Z. morio* were purchased from reptilefood.com. Preparation of *T. molitor* Extracts: *Lot T15813*: 550 *T. molitor* larvae (51.4 g in fresh weight) were dried in an oven at 55°C for 24 h to obtain 15.2 g dried matter. The dried matter was ground to a coarse powder and extracted three times for 24 h each with 100 mL of 95% EtOH each time at room temperature. The combined EtOH extracts were concentrated under reduced pressure to produce 1.47 g extracts with the yield of about 2.7 mg extracts per worm. *Lot T15911*: 1,500 *T. molitor* larvae (118.7 g in fresh weight) were dried in an oven at 55°C for 24 h to obtain 55.2 g dried matter. The dried matter was ground to a coarse powder and extracted three times for 24 h each with 100 mL of 95% EtOH each time at room temperature. The combined EtOH extracts were concentrated under reduced pressure to produce 5.67 g extracts with the yield of about 3.8 mg extracts per worm. Preparation of *Z. morio* Extracts: *Lot Z15721*: 100 *Z. morio* larvae (66.14 g in fresh weight) were dried in an oven at 55°C for 24 h to obtain 24.2 g dried matter. The dried matter was ground to a coarse powder and extracted three times for 24 h each with 100 mL of 95% EtOH each time at room temperature. The combined EtOH extracts were concentrated under reduced pressure to produce 2.03 g extracts with the yield of about 20.3 mg extracts per worm. *Lot Z15911*: 300 *Z. morio* larvae (61.2 g in fresh weight) were dried in an oven at 55°C for 24 h to obtain 27.5 g dried matter. The dried matter was ground to a coarse powder and extracted three times for 24 h each with 100 mL 95% of EtOH each time at room temperature. The combined EtOH extracts were concentrated under reduced pressure to produce 2.59 g extracts with the yield of about 8.63 mg extracts per worm. Spray experiment of *T. molitor* Extracts (T15813): 360 mg *T. molitor* extracts were dissolved and suspended in nanopure H_2O and prepared as 7.2 mL experimental solution at the concentration of 5% extracts. The larvae of *T. molitor* (T15813) and *Z. morio* (Z15721) were cultured separately in plastic containers fed with fresh apples purchased from the local grocery store. 90 larvae of each species were sprayed with 3.6 mL of H_2O only to serve as controls. 90 larvae of each species were sprayed with 3.6 mL of 5% *T. molitor* extracts, respectively (2 mg *T. molitor* extracts per worm). Survival status of each worm was documented and photographed 24

h after the treatment. Dermal Contact Experiment of *T. molitor* and *Z. morio* Extracts: Randomly selected 10 active larvae of *T. molitor* or *Z. morio* cultured in a petri dish were subjected to one of the following applications on the worm body with a pipettor: 10 µL H_2O per worm (controls), 1, 2, 3, 5, 10, or 20 mg *T. molitor* extracts (T15721 or T15911) or *Z. morio* extracts (Z15813 or Z15911) per worm of *T. molitor* and *Z. morio*. Each experiment had three replications. Survival status of each worm was documented and photographed 24 h after the treatment. Impact of *T. molitor* Extracts (T15813) on Red Imported Fire Ant: The red imported fire ants (*S. invicta*) were collected at Nacogdoches, Texas, USA. 1,000 ants (approximately 1 g in fresh weight) were put in 5 L plastic beaker and sprayed with 1 mL of 5% *T. molitor* extracts. Another 1,000 ants in 5 L plastic beaker were sprayed with nanopure H_2O as the controls. The ant viability of the treatment and control groups were observed for 24 h. The data were analyzed by SPSS 13.0 for Windows (SPSS, Inc., Chicago, IL). ANOVA with Duncan post-hoc analysis was used to compare the means of the control and treatment groups.

Results: During these experiments, all worms of each species in the control group were actively alive by 24 h after the treatment. By the same time, 35.6% of *T. molitor* and 10% of *Z. morio* worms that were sprayed with 3.6 mL of 5% *T. molitor* extracts were dead. The spray dosage (2 mg per worm or 21.4 µg per mg of fresh worm body weight) was lower than the extraction yield per *T. molitor* worm (2.7 mg). During the 24 h of dermal contact experiments, the larvae of each species was more susceptible to its own extracts than to the other at the same dosage. In resoponse to the extracts of the same species, larval fatality of each species increased with application dosage. 33-40% of *T. molitor* and 37-97% of *Z. morio* were dead when treated with the same species extracts at the dosage equivalent to the extraction yield per worm (Table S7). Effects of the extracts also depend on the body weight of testing worm. Adult beetles of *T. molitor* were also susceptible to the extracts of both species, particularly at higher dosage (5 mg per worm). The 5% *T. molitor extracts* (at the dosage of 50 µg per mg of fresh ant body weight, about 2.34 times of spray dosage in *T. molitor*) had no effects on viability of the red imported fire ant (*S. invicta*) compared with the control experiment during the 24 h of observation.

3. Antidotal Action on Autotoxicity of a Species by Its Non-closely Related Species

3.1. Growth Differences of Four Aquatic Plant Species in Water Culture of Pure Population and in Mixed Population

General Experimental Procedures: The experiments were conducted in the greenhouse (30°C during the day time and 20°C at night) with four aquatic plant species that are non-closely related species each other but often grow together in nature. The healthy plants of giant salvinia (*S. molesta*) (10 g primary stage and 100 g tertiary stage), water lettuce (*P. stratiotes*) (20 g), water hyacinth (*E. crassipes*) (100 g), and Parrot feather (*Myriophyllum aquaticum* (Vell.) Verdc., Haloragaceae) (50 g) were cultured alone each species or mixed population in the container with 25 L tap water for 3 months, respectively. Each experiment had three replications with a total 15 containers. By the end of three months, biomass of living plants in each container was measured. The data were analyzed by SPSS 13.0 for Windows (SPSS, Inc., Chicago, IL). ANOVA with Duncan post-hoc analysis was used to compare the means of the control and treatment groups.

Results: During the three months of observation, all species of *P. stratiotes, E. crassipes,* and *M. aquaticum* experienced significant decrease in living biomass in pure culture. *S. molesta* had growth in living biomass because the addition of 10 g primary stage of plants which were less affected as the plants in tertiary stage by the salvinia endocide. During the three months of experiments, living biomass of *S. molesta*, water lettuce (*P. stratiotes* L., Araceae), water hyacinth (*Eichhornia crassipes* (Mart.) Solms, Pontederiaceae), and Parrot feather (*Myriophyllum aquaticum* (Vell.) Verdc., Haloragaceae) in the mixed culture of four species increased by 76.2%, 127.6%, 133.3%, and 664% than that in the pure culture of single species, respectively (Supplementary Fig. S53).

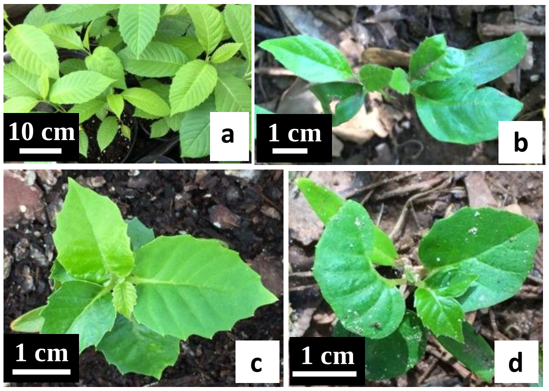

Supplementary Figure S1 Effects of *Camptotheca* fruit soaking in water on leaf development. (**a**) Normal leaves of *C. acuminata* seedling in the greenhouse without fruit soaking. (**b**) Abnormal leaves of *C. acuminata* seedling under the parent in the field after three months of saturated water soaking by rain water. (**c**) Leaves of *C. lowreyana* 'Hicksii' seedling germinated in the greenhouse without fruit soaking. (**d**) 'Hicksii' seedling germinated under the mature 'Hicksii' tree after three months of saturated water soaking by rain water.

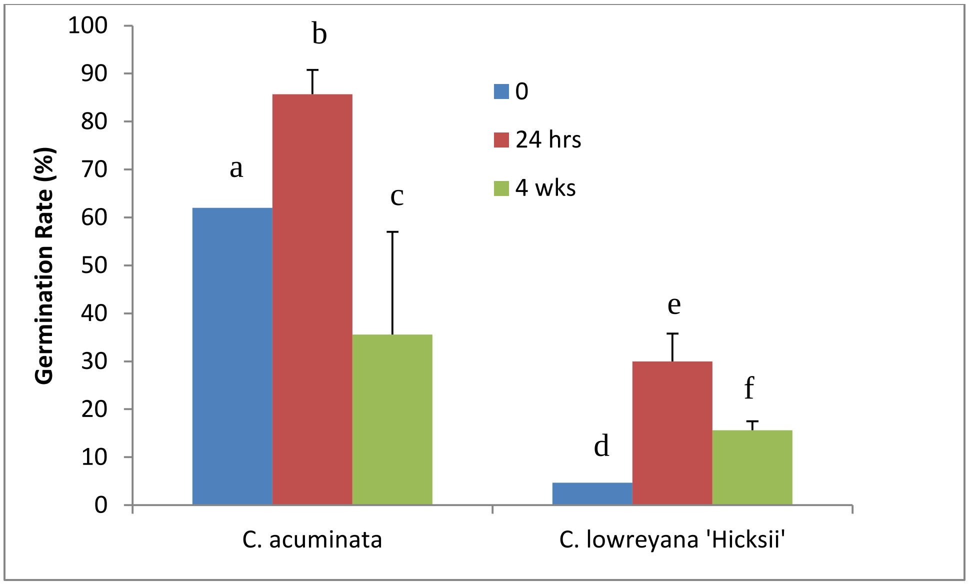

Supplementary Figure S2 Effect of soaking time length of *Camptotheca acuminata* fruits in water on germination rate. Significant differences among germination rate values of each plant are indicated by different letters ($P < 0.05$).

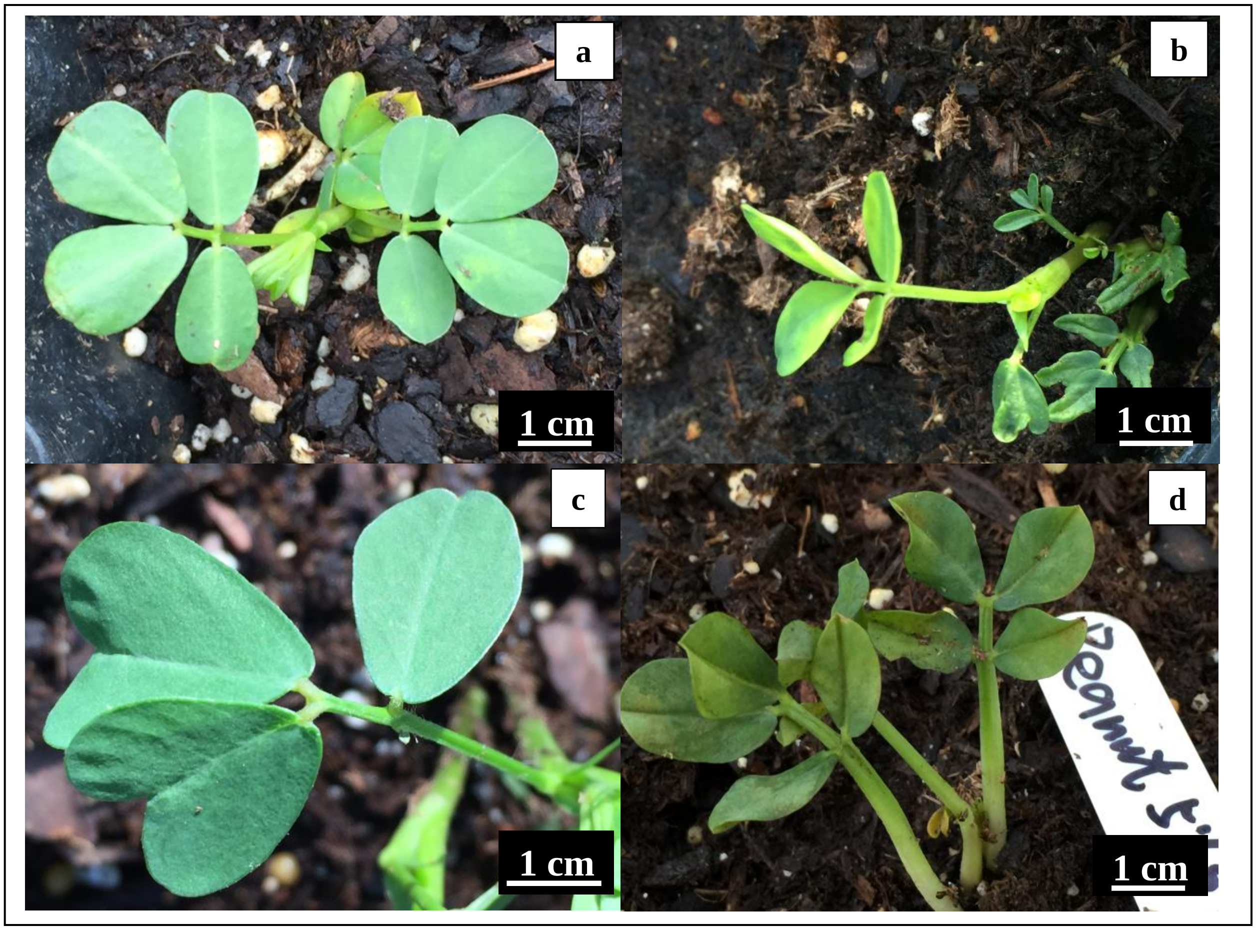

Supplementary Figure S3 Seedlings of peanut (*Arachis hypogaea* L.) germinated from the seeds soaked in 5% EtOH extracts of peanut shell for five weeks developed abnormal leaf morphogenesis (**b**, **c**, and **d**) in comparison with normal seedlings from germinated from the seeds without any treatment (**a**).

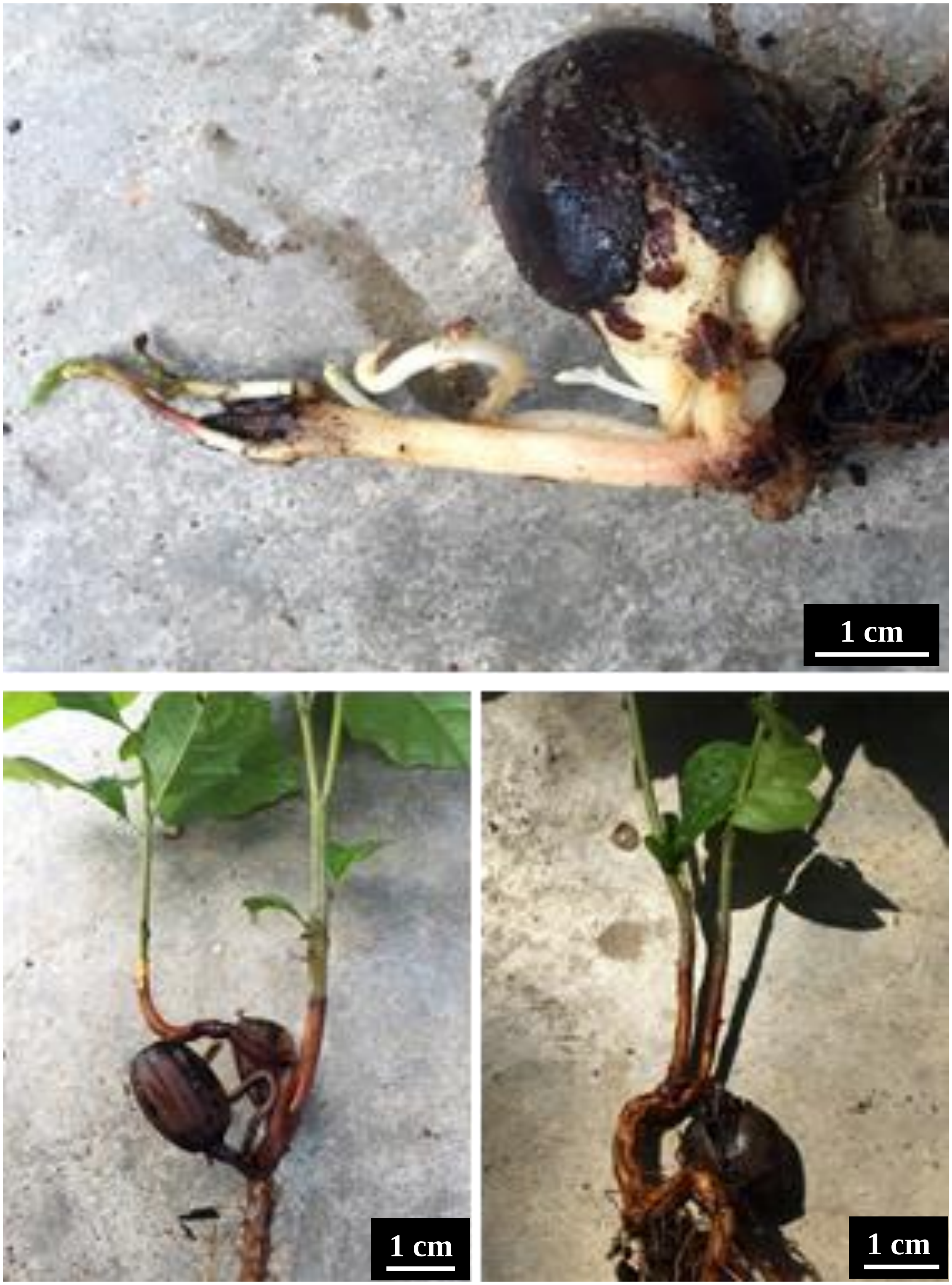

Supplementary Figure S4 About 18% of the germinated acorns of *Quercus shumardii* developed multiple stems after the acorns soaked in a treatment of 5% *Q. shumardii* acorn extracts.

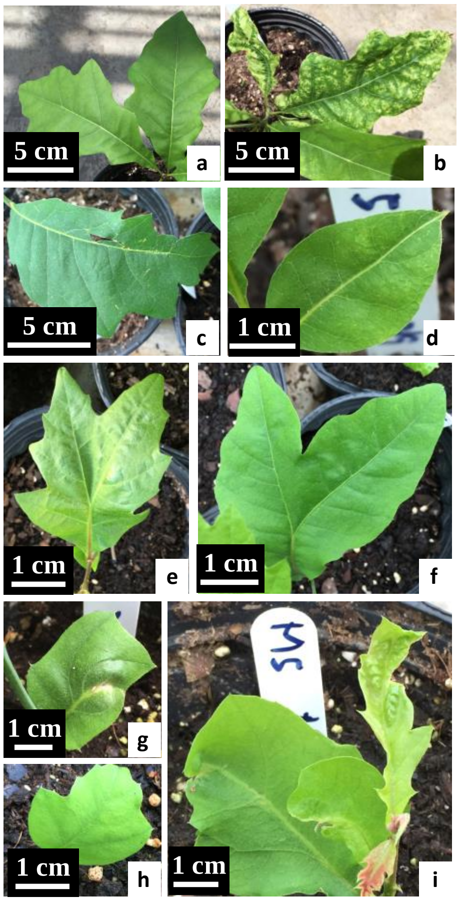

Supplementary Figure S5 Abnormal leaf morphogenesis developed in the early leaves of *Quercus shumardii* after the acorns soaked in 5% EtOH extracts of *Q. shumardii* acorns. (**a**) Normal leaves. (**b-i**) Various induced abnormal leaves.

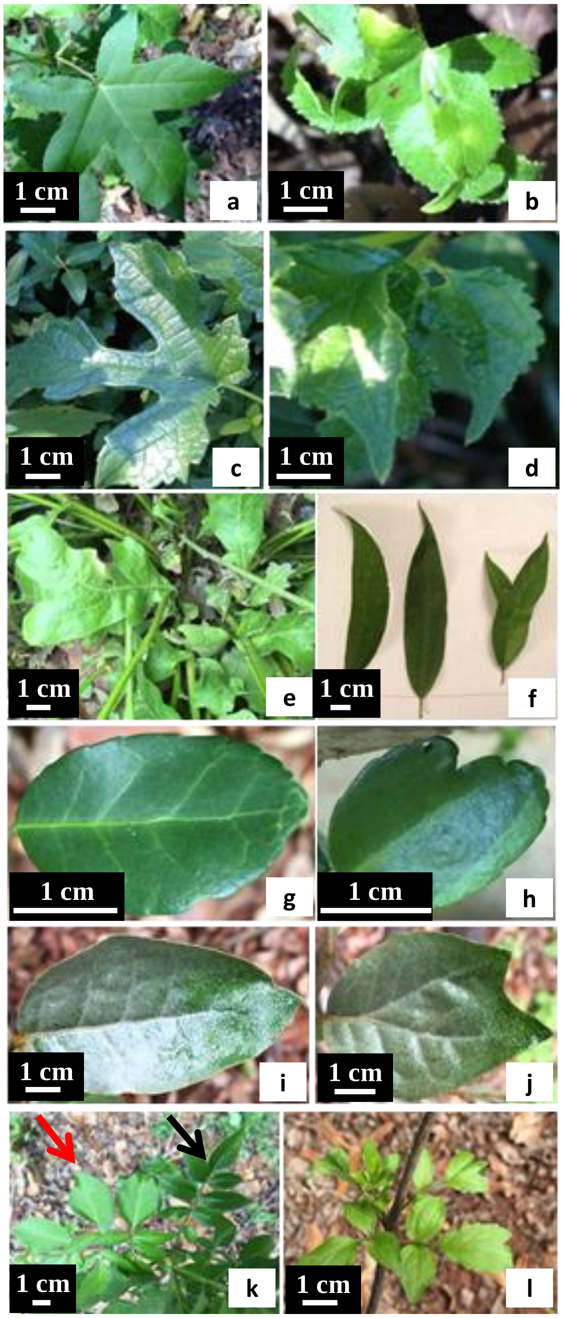

Supplementary Figure S6 Induced abnormal leaf morphogensis of six simple-leaf plant species and one compound leaf species after decapitation pruning. (**a**) Normal leaves of *Liquidambar styraciflua*. (**b**) Abnormal leaves of *L. styraciflua*. (**c**) Normal leaves of *Morus alba*. (**d**) Abnormal leaves of *M. alba*. (**e**) Both normal and abnormal leaves of *Quercus shumardii*. (**f**) Both normal and abnormal leaves of *Prunus persica*. (**g**) Normal leaf of *Ilex vomitoria*. (**h**) Abnormal leaf of *I. vomitoria*. (**i**) Normal leaf of *Elaeagnus pungens*. (**j**) Abnormal leaf of *E. pungens*. (**k**) *Sambucus canadensis* (black arrow-normal compound leaf; red arrow-abnormal twice compound leaf). (**l**) Lobed leaflets of *S. canadensis*.

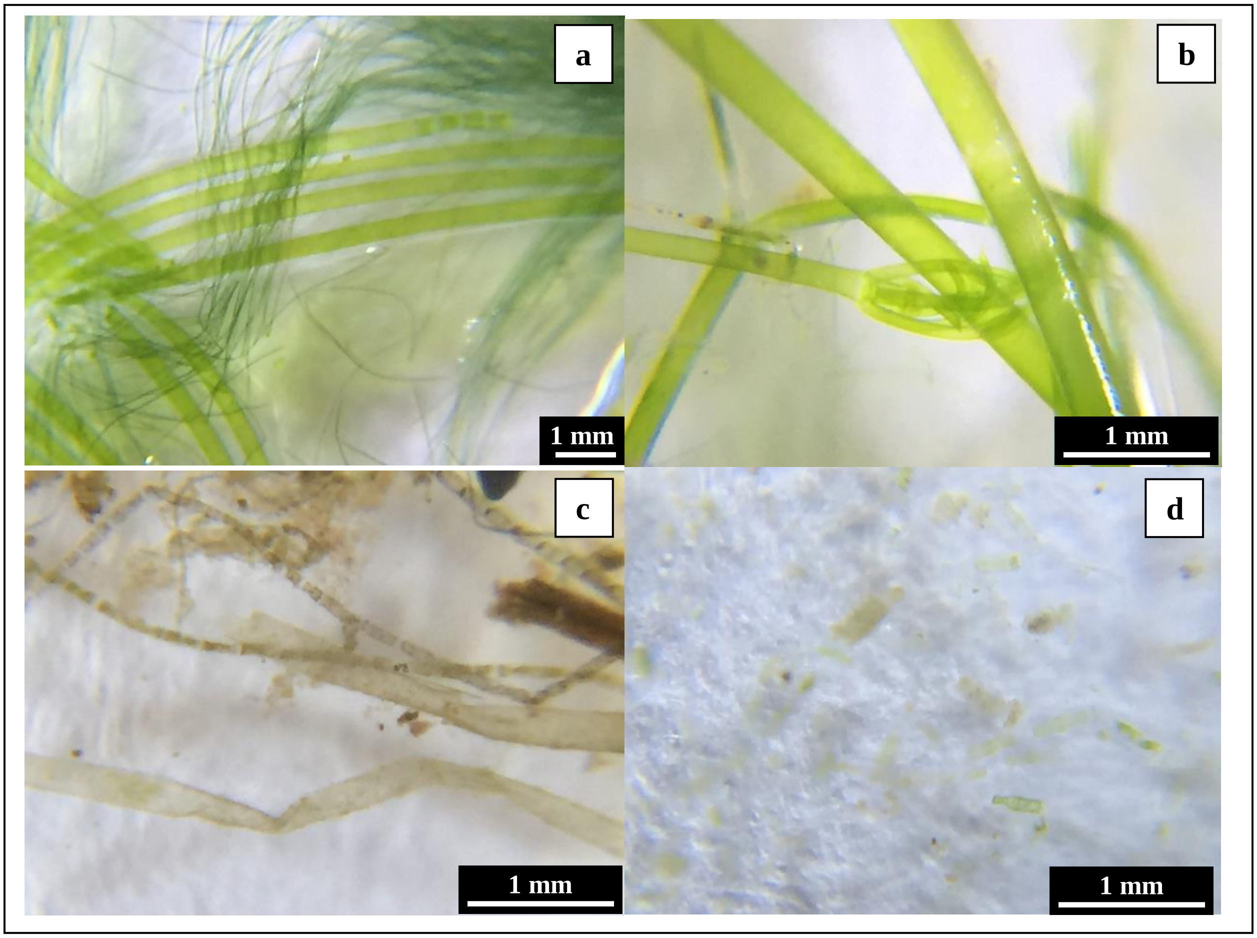

Supplementary Figure S7 Majority of the plant cells of muskgrass (*Chara vulgaris*) (**c**) and filamentous algae (*Cladophora* sp. and *Pithophora roettleri*) (**d**) were separated and lost green color after 48 h of the treatment of EtOH extracts of *C. vulgaris* in comparison with those under control (**a**: *C. vulgaris*; **b**: *Cladophora* sp. and *P. roettleri*).

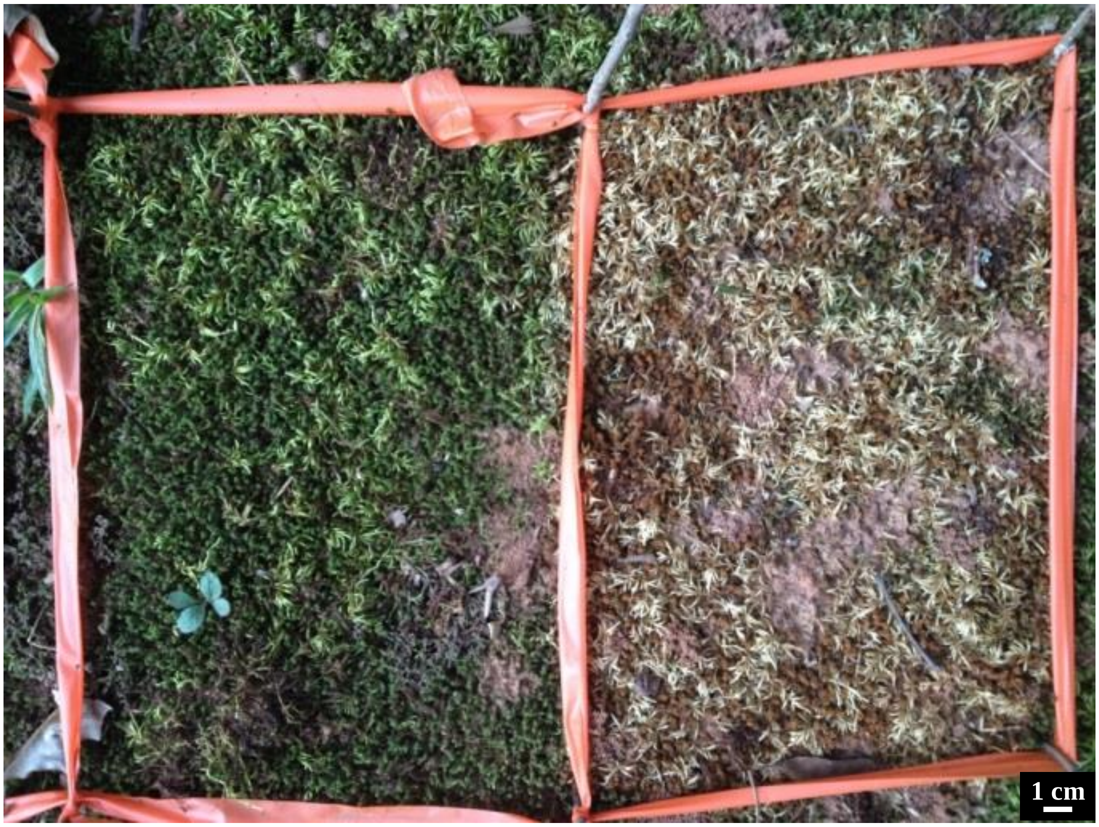

Supplementary Figure S8 Both seductive entodon moss (*Entodon seductrix* C. Müller) and atrichum moss (*Atrichum angustatum* (Brid.) Bruch & Schimp.) were dead within four weeks of the foliar application of 10% EtOH extracts of mosses with 1% Tergitol (left: control, right: treatment).

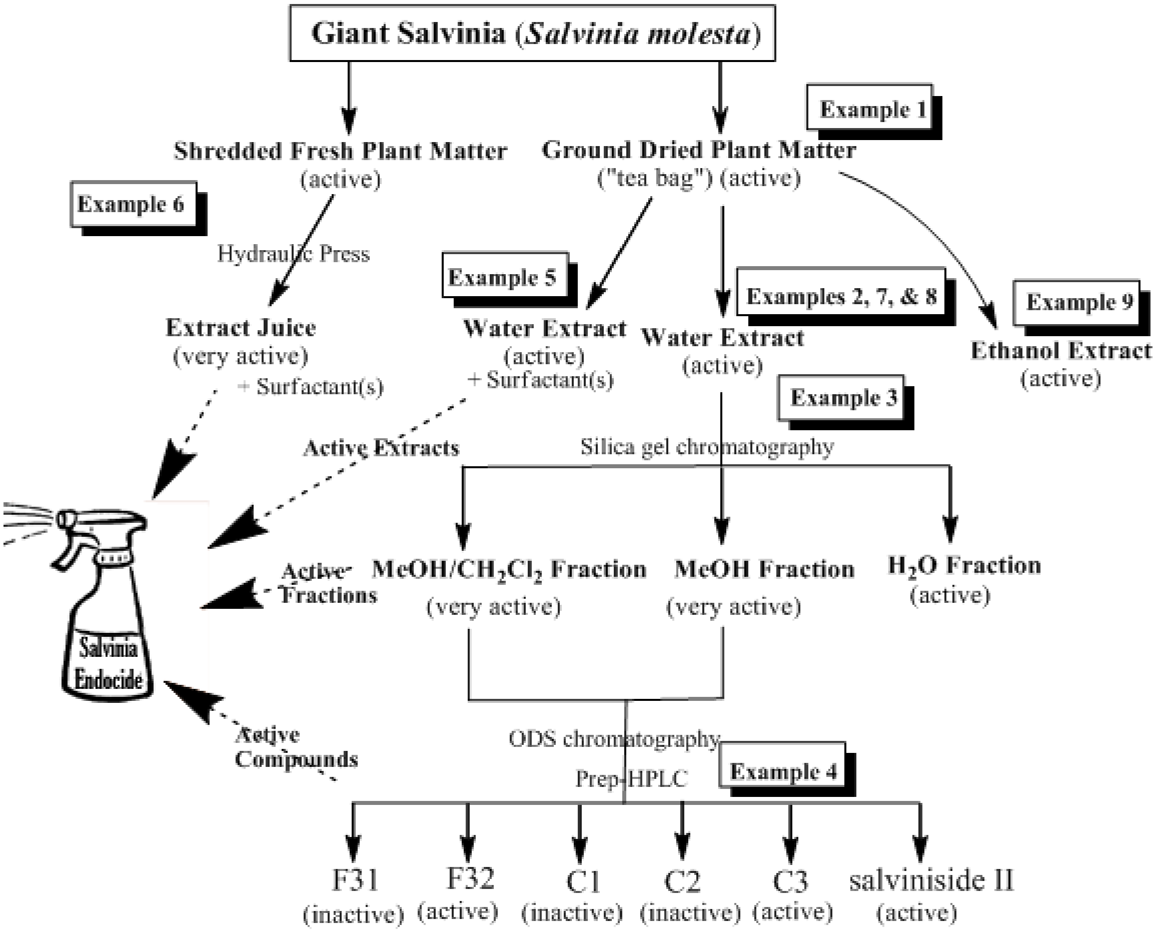

Supplementary Figure S9 The scheme shows the discovery and development process and related experiments of endocides from invasive giant salvinia (*Salvinia molesta*) as described herein (inactive: not able to inhibit the growth of giant salvinia at all; active: able to fully inhibit the growth of *S. molesta* at high concentrations; very active: able to fully inhibit the growth of *S. molesta* at low concentrations).

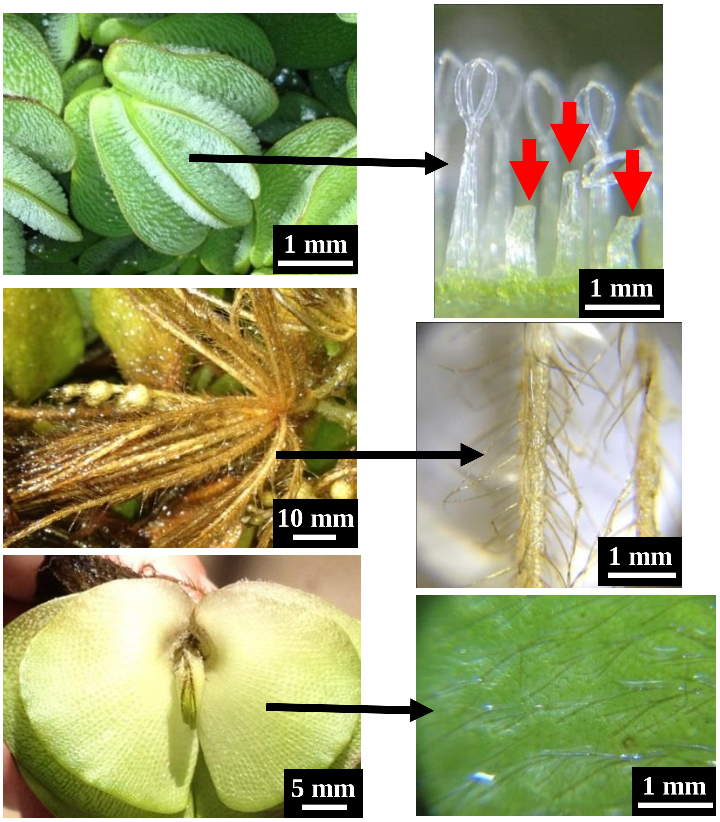

Supplementary Figure S10 *Salvinia* species are rootless, free-floating aquatic ferns and they are well-known for their extremely water-repellent floating leaves. On the upper surfaces of the floating leaves of *S. molesta*, four multicellular glandular trichomes (called "trichomes") have their apical cells connected to form egg-beater structures. The three short arrows point to the egg-beater structures of trichomes which were removed using a blade. The root-like submerged leaves are highly branched with multicellular trichomes on which sporocarps develop.

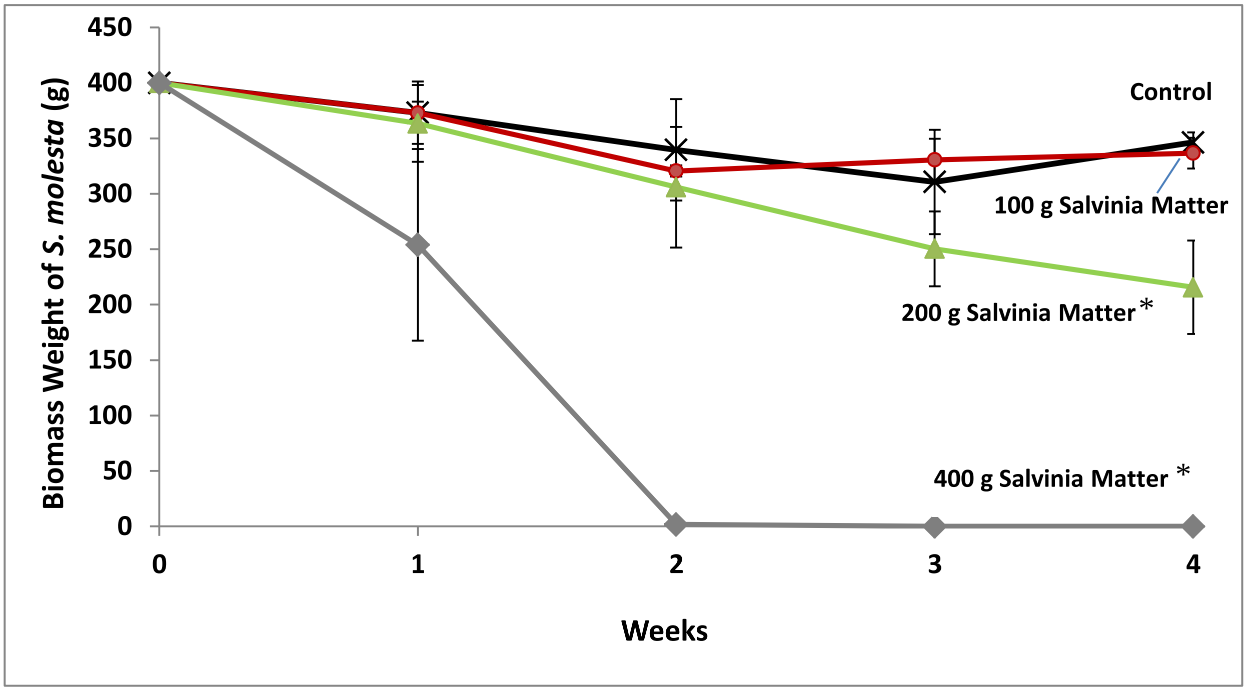

Supplementary Figure S11 Effects of dry plant matter of giant salvinia (*Salvinia molesta*) ("tea bag") on the growth of *S. molesta* in containers during the four weeks of greenhouse experiments (with bars presenting standard deviations). Asterisks denote significant differences between treatment and control values at the end of four weeks ($P < 0.05$).

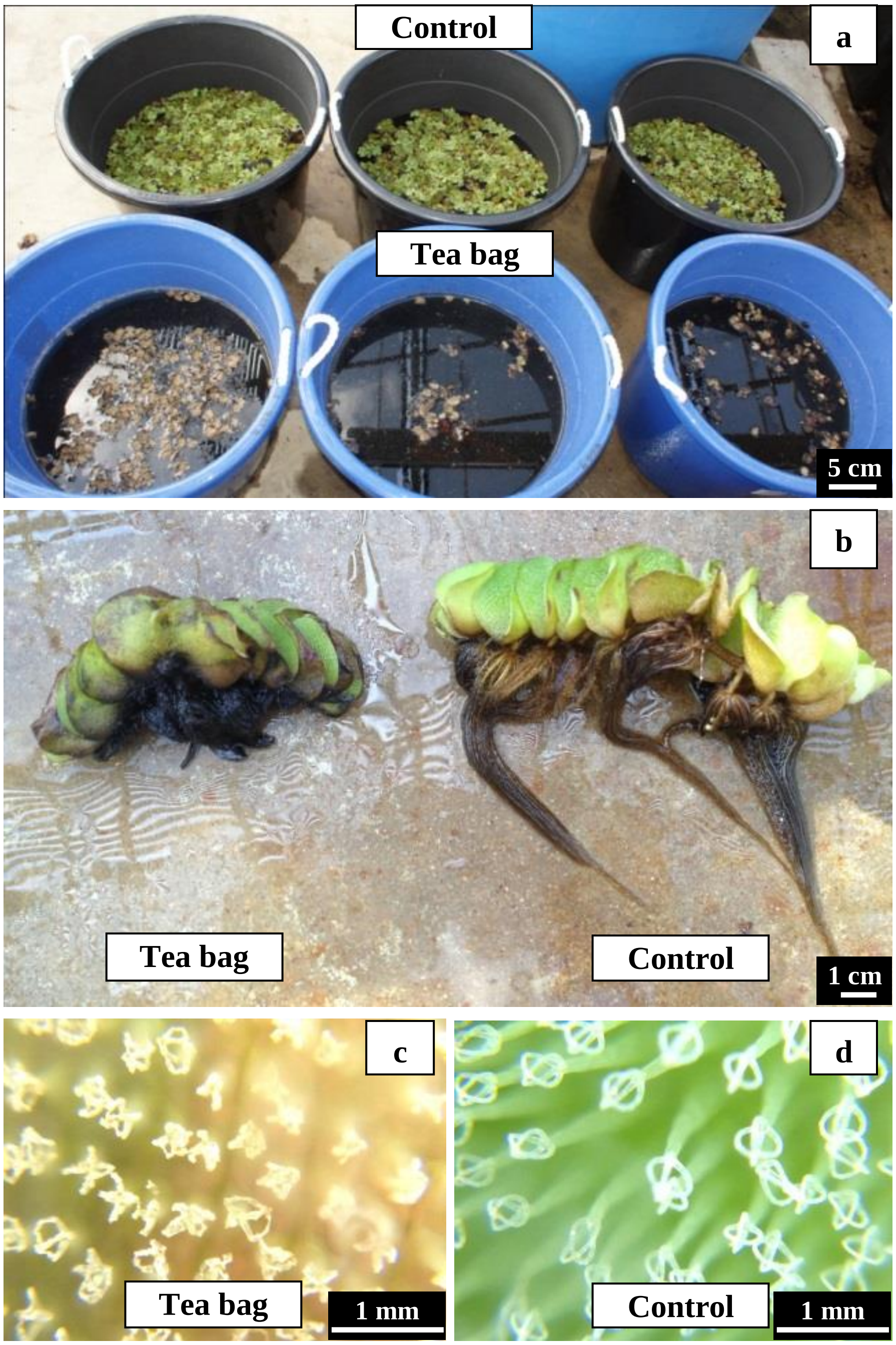

Supplementary Figure S12 The photograph (**a**) shows that giant salvinia (*Salvinia molesta*) plants were totally eliminated by the dried plant matter of *S. molesta* (a bag of 400 g plant matter was placed in the bottom of each of the containers on the bottom row) (called "tea bag") by the end of the second week of experiments in the greenhouse in comparison with controls (containers on the top row); there was no new growth within the six months of observation. The middle photograph (**b**) shows that individual plants in response to the tea bag treatment by the end of the first week with significant trichome changes (**c** and **d**).

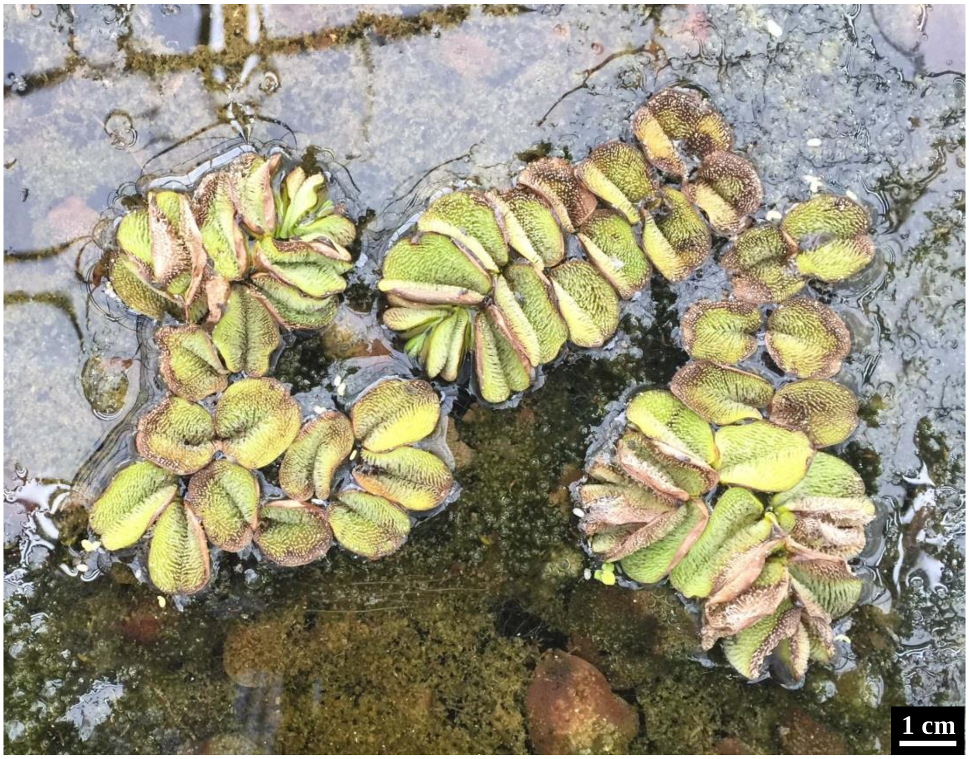

Supplementary Figure S13 The photograph shows that young giant salvinia (*Salvinia molesta*) plants in primary stage has initial slight growth but soon became unhealthy by the end of two weeks in the 400 g “tea bags” treatment.

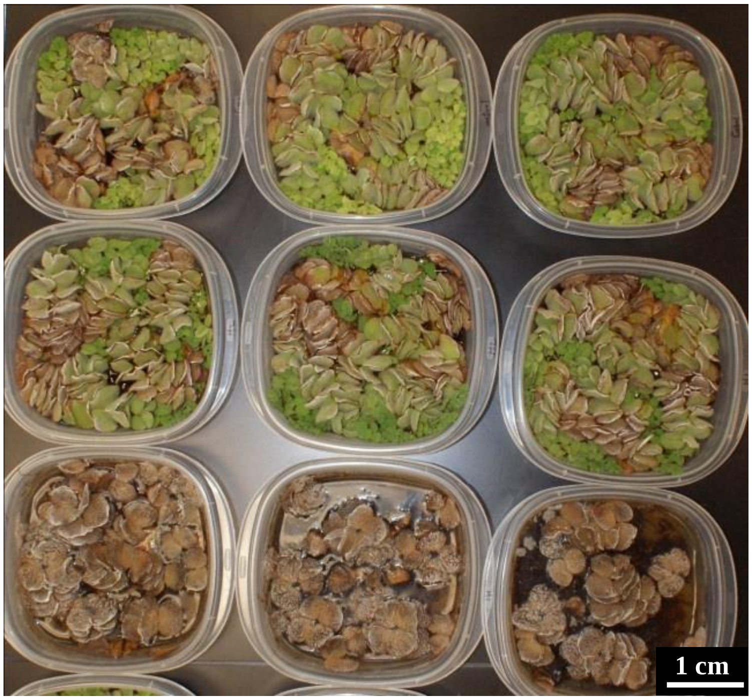

Supplementary Figure S14 The photograph shows that all giant salvinia (*Salvinia molesta*) plants were eliminated within the 13 weeks after the treatment with H_2O extracts of the dried matter of *S. molesta*. The plants in the top row served as control without any treatment, and each plant has developed significant new growth. The plants in the middle row were treated with 0.01% H_2O extracts of *S. molesta*, and some plants have developed new growth. The plants in the bottom row were treated with 0.1% H_2O extracts of *S. molesta*, and all plants are dead and no new growth was developed from any plants during the eight months of observation. The three columns represent three replications of the experiment.

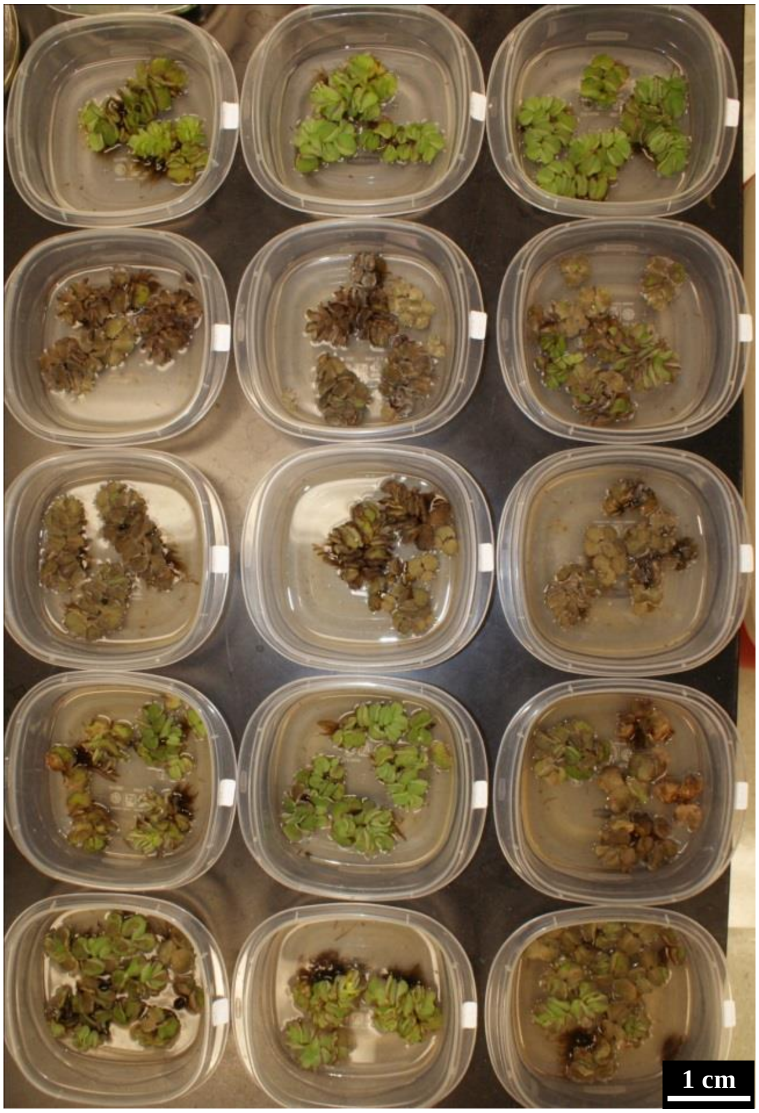

Supplementary Figure S15 The photograph showing growth performance of giant salvinia (*Salvinia molesta*) plants two weeks after the treatments of H_2O extracts of the dried matter of *S. molesta* and its three fractions in comparison with those in the control group. Top row: plants cultured in water without any treatment (control); 2nd row: plants were treated with 0.1% MeOH fraction (all plants dead); 3rd row: plants were treated with 0.1% MeOH/CH_2Cl_2 (1:1) fraction; 4th row: plants were treated with 0.1% H_2O fraction; and the bottom row: 0.1% H_2O extracts of *S. molesta*. The three columns represent three replications of the experiment.

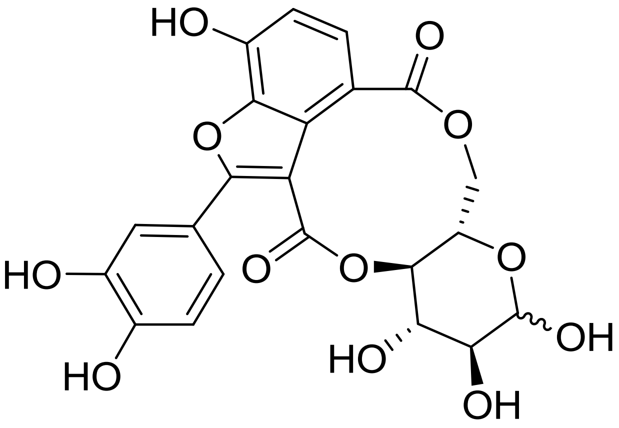

Supplementary Figure S16 Structure of salviniside II isolated from giant salvinia (*Salvinia molesta*).

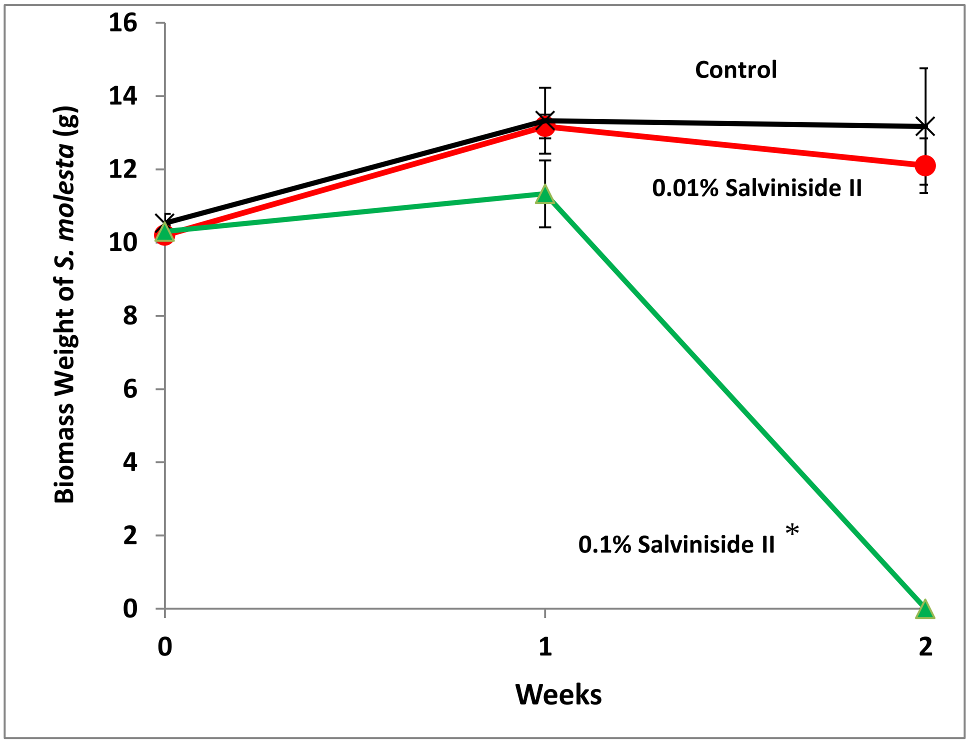

Supplementary Figure S17 Giant salvinia (*Salvinia molesta*) can be totally eliminated by salviniside II isolated from *S. molesta* within two weeks (with bars presenting standard deviations). Asterisk denotes a significant difference between treatment and control values at the end of two weeks ($P < 0.05$).

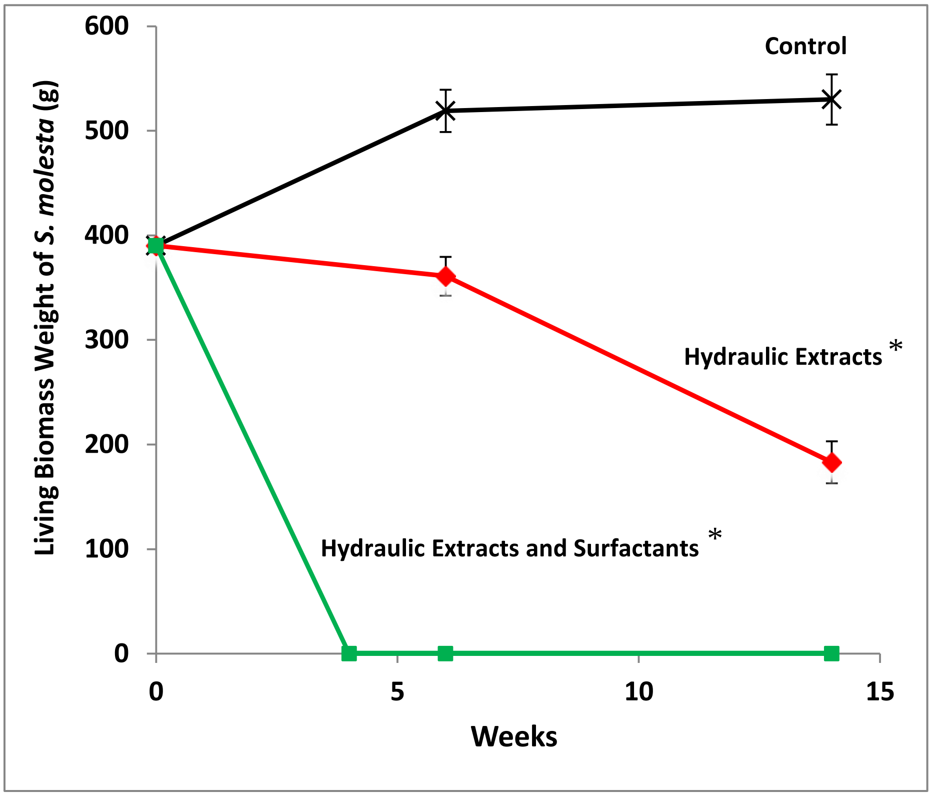

Supplementary Figure S18 The growth of giant salvinia (*Salvinia molesta*) can be successfully controlled by the extract juice from the fresh matter of *S. molesta* by hydraulic press. After two treatments, the hydraulic extract juice with surfactants totally eliminated the *S. molesta* within four weeks and there was no new growth thereafter (with bars presenting standard deviations). Asterisks denote significant differences between treatment and control values at the end of 14 weeks ($P < 0.05$).

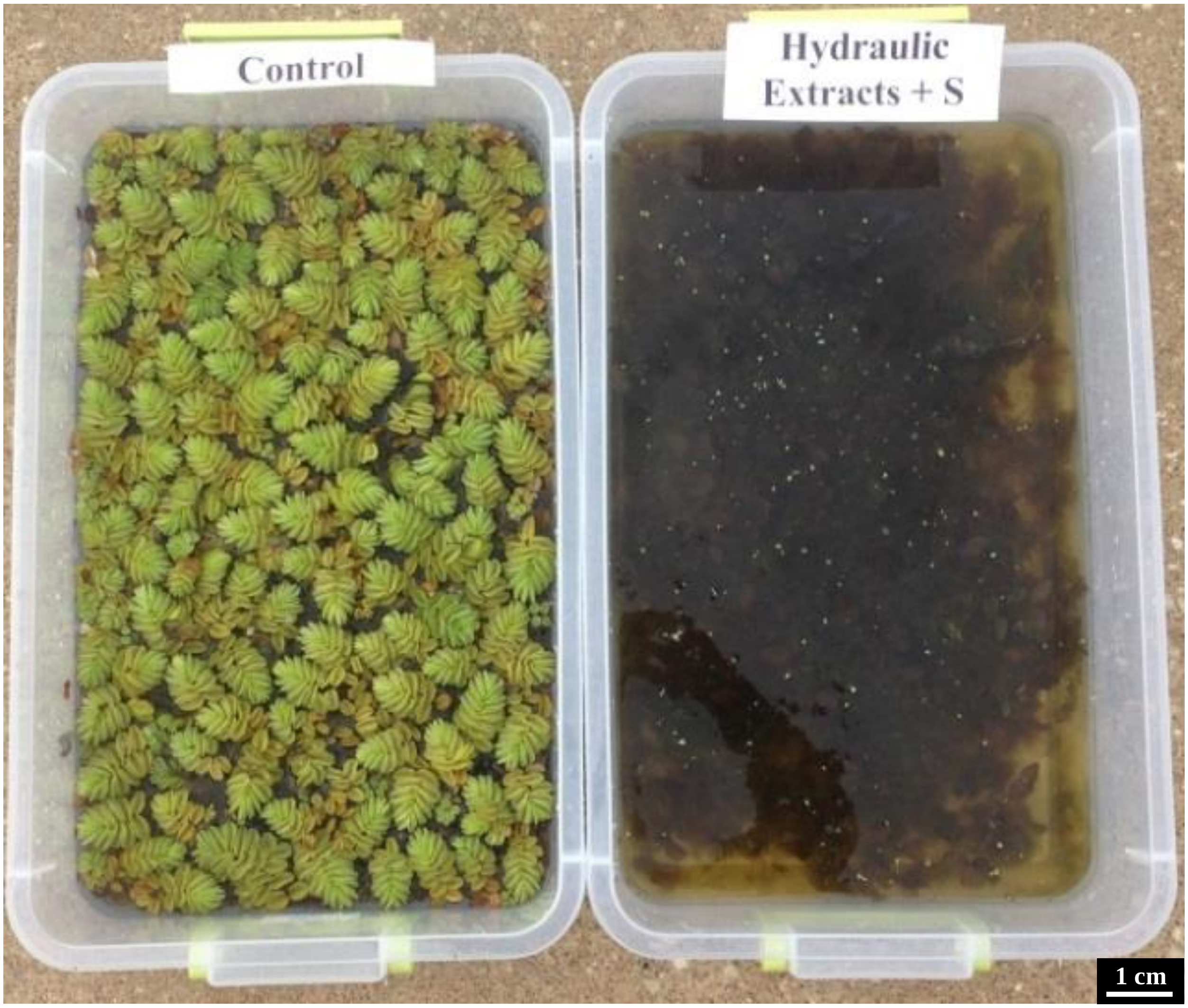

Supplementary Figure S19 The photograph shows that all giant salvinia (*Salvinia molesta*) plants were eliminated within four weeks after the treatment with hydraulic extract juice from the fresh matter of *S. molesta* (right) in comparison with the control (left).

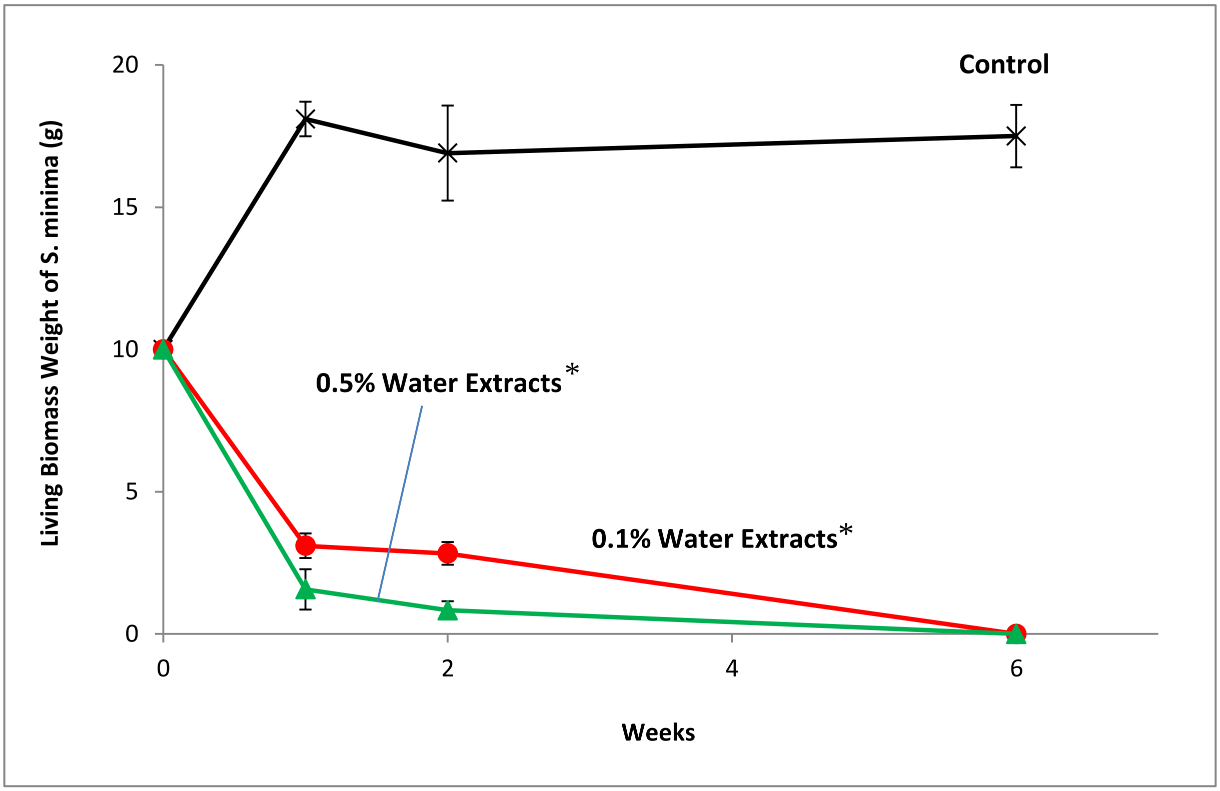

Supplementary Figure S20 The diagram shows that the growth of the floating fern (*Salvinia minima*) plants can be totally controlled by the water extracts of dried matter of giant salvinia (*Salvinia molesta*) within six weeks (with bars presenting standard deviations). Asterisks denote significant differences between treatment and control values at the end of six weeks ($P < 0.05$).

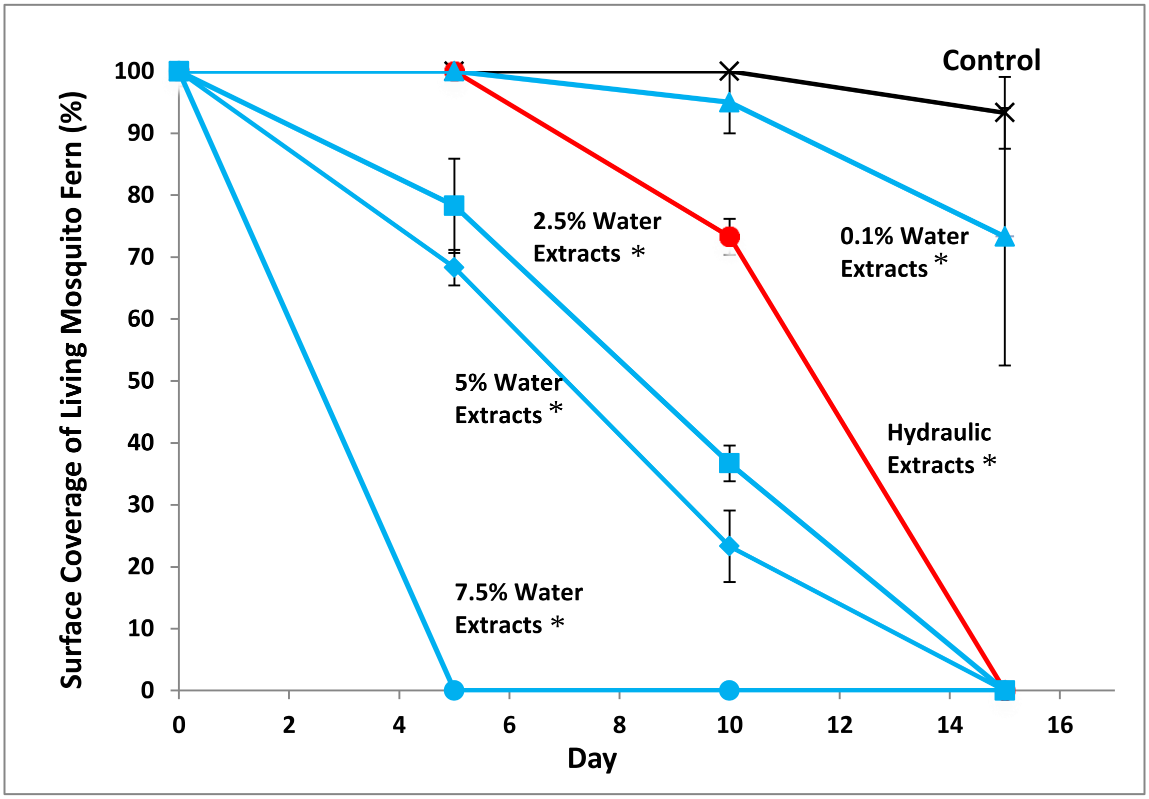

Supplementary Figure S21 The diagram shows that the plants of Carolina mosquito fern (*Azolla caroliana*) was inhibited by the H_2O extracts at higher concentration (2.5% or higher, in dry weight) (with bars presenting standard deviations). Asterisks denote significant differences between treatment and control values at the end of 15 weeks ($P < 0.05$).

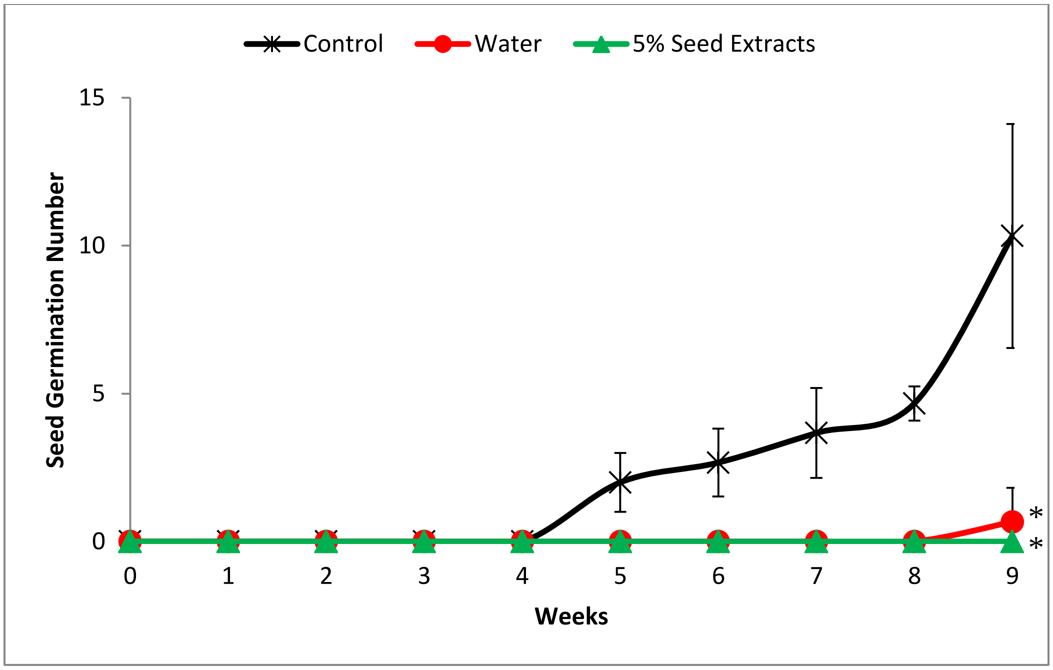

Supplementary Figure S22 The Impact of Korean pine (*Pinus koraiensis* Siebold & Zucc.) seed extracts and prolonged water soaking on the seed germination of *P. koraiensis*. Asterisks denote significant differences between treatment and control values at the end of four weeks ($P < 0.05$).

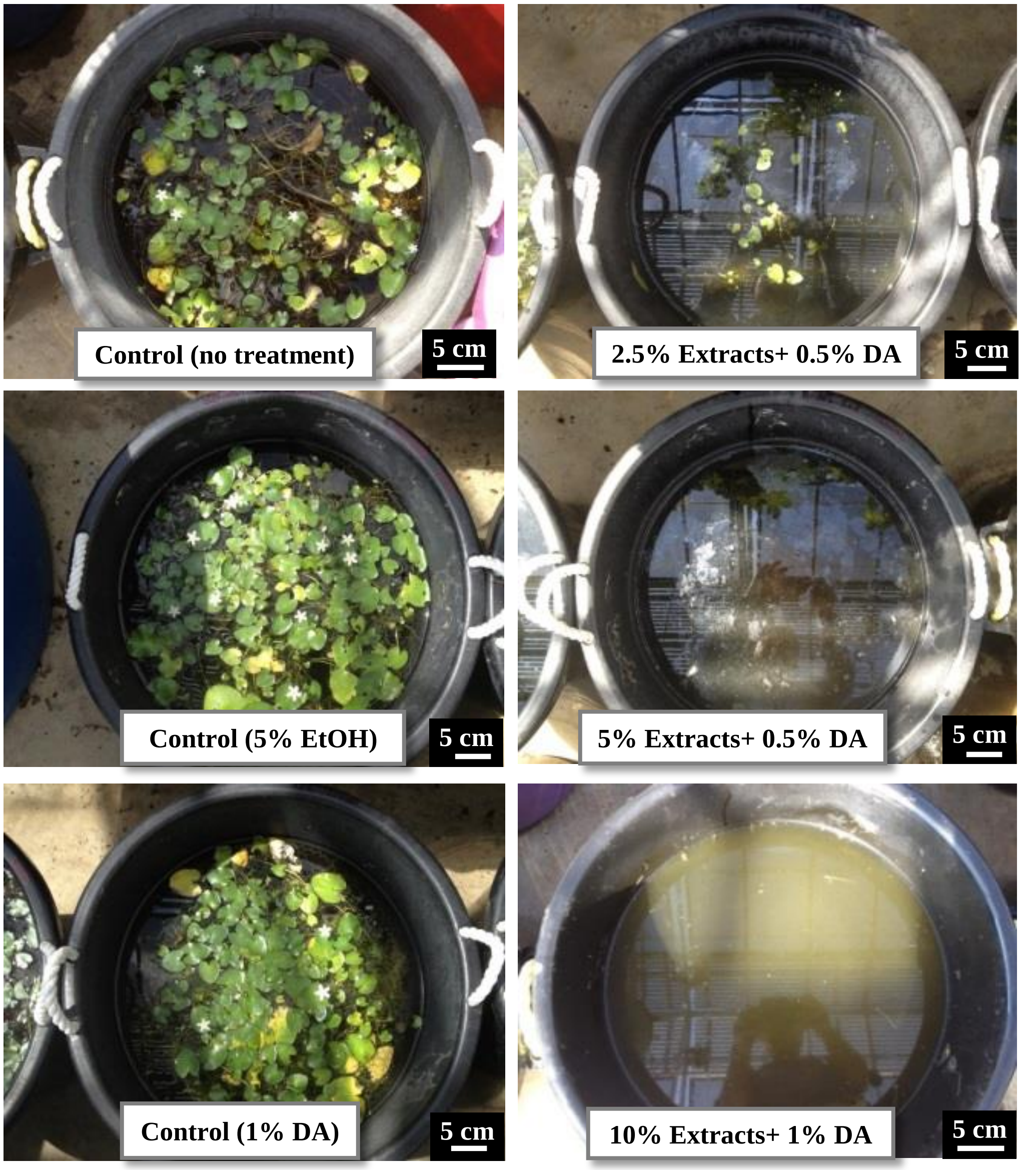

Supplementary Figure S23 The photos showing the result of the foliar application of EtOH extracts of crested floating heart (*Nymphoides cristata*) on *N. cristata* plants by the end of 4th week of the treatments (DA: Dyne-Amic).

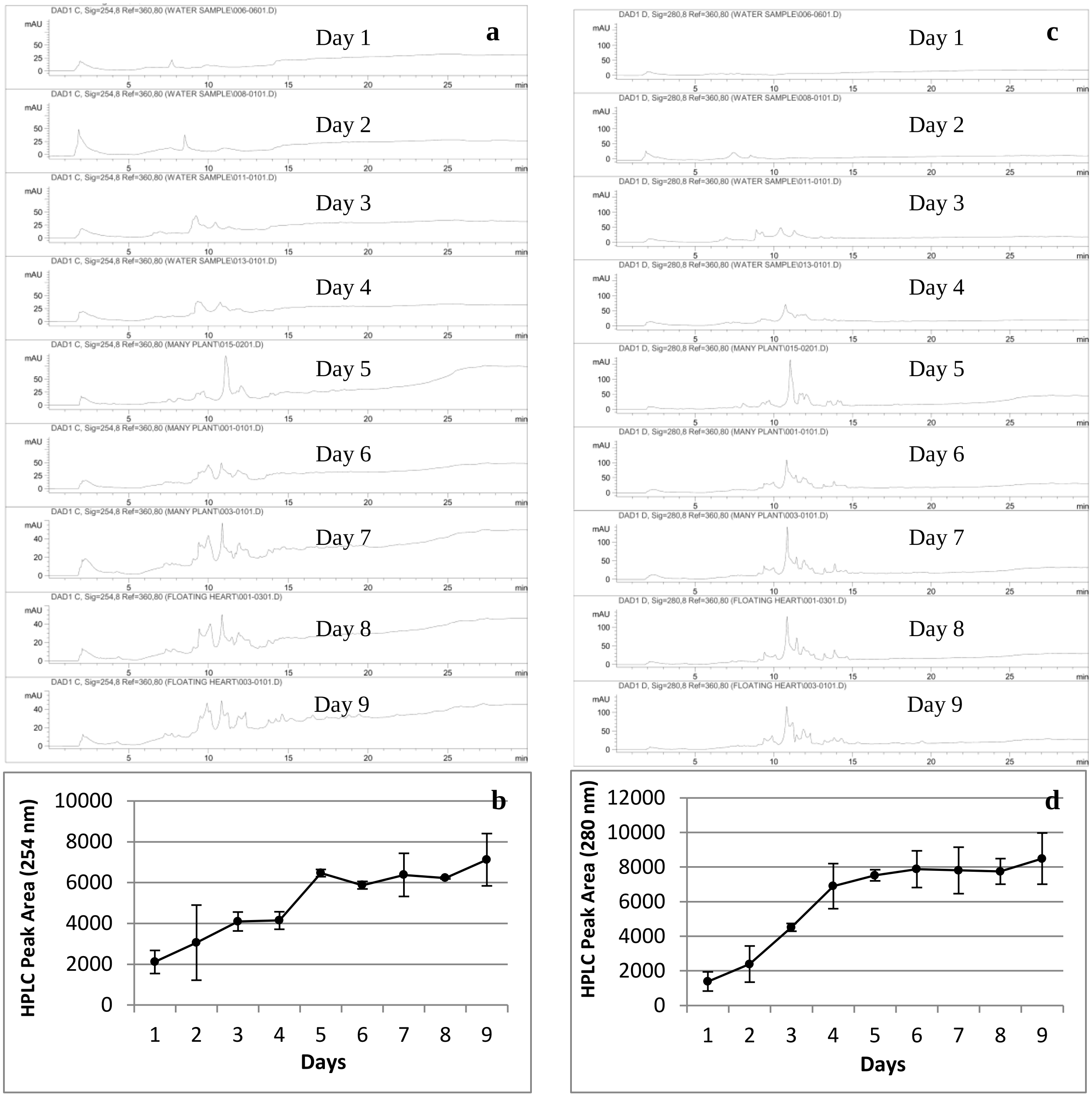

Supplementary Figure S24 HPLC chromatograms show that the changes of the total chemical contents in the liquid part of the whole fresh matter of crested floating heart (*Nymphoides cristata*) during the nine days of experiment. **a**. daily HPLC chromatograms of the liquid part at 254 nm, b. daily change of the total HPLC peak area of the liquid part at 254 nm, **c**. daily HPLC chromatograms of the liquid part at 280 nm, and **d**. daily change of the total HPLC peak area the liquid part at 280 nm.

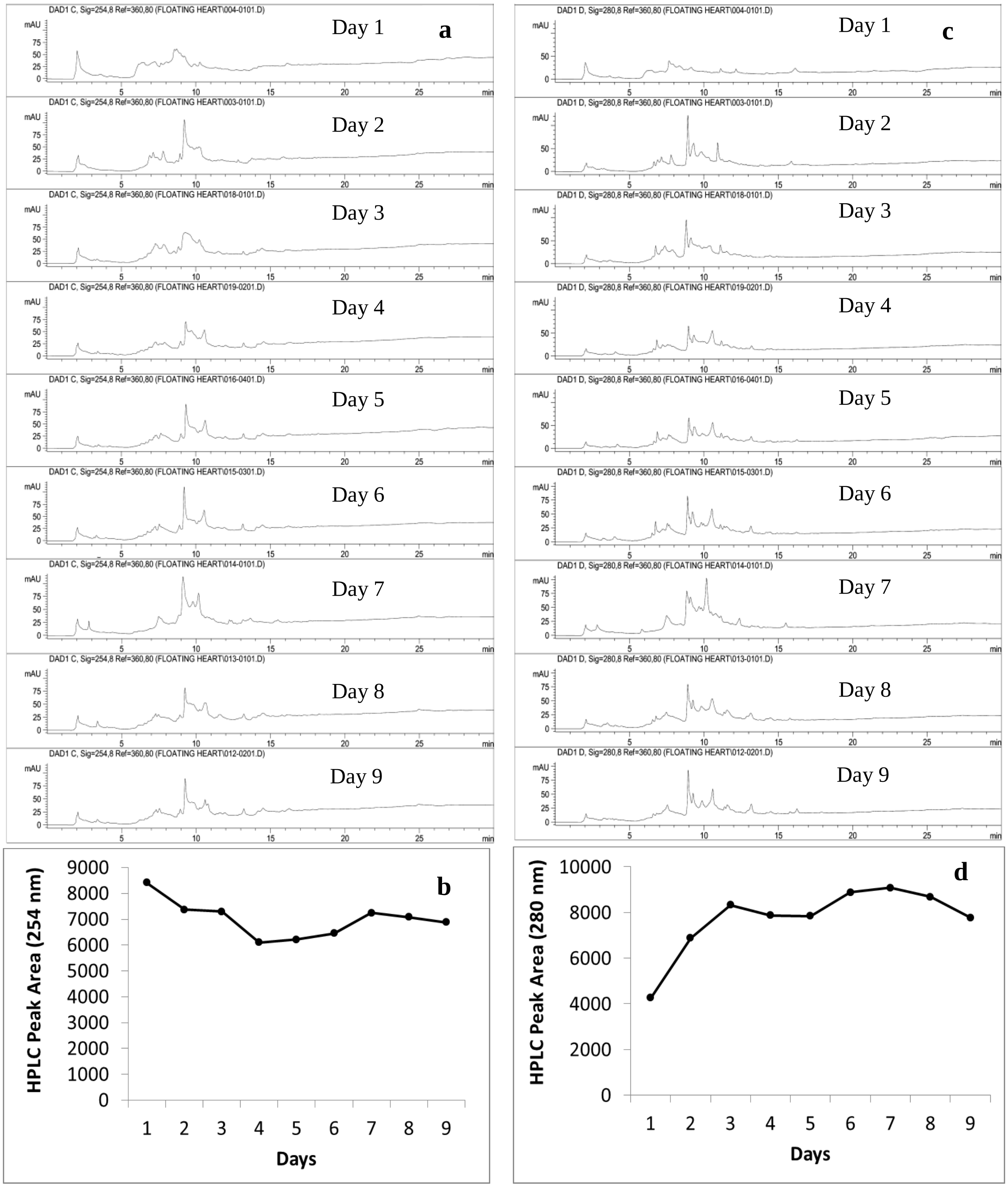

Supplementary Figure S25 HPLC chromatograms show that the changes of the chemical contents in the H_2O extracts of the shredded fresh matter of crested floating heart (*Nymphoides cristata*) during the nine days of experiment. **a**. daily HPLC chromatograms of the H_2O extracts at 254 nm, **b**. daily change of the total HPLC peak area of the H_2O extracts at 254 nm, **c**. daily HPLC chromatograms of the H_2O extracts at 280 nm, and **d**. daily change of the total HPLC peak area of the H_2O extracts at 280 nm.

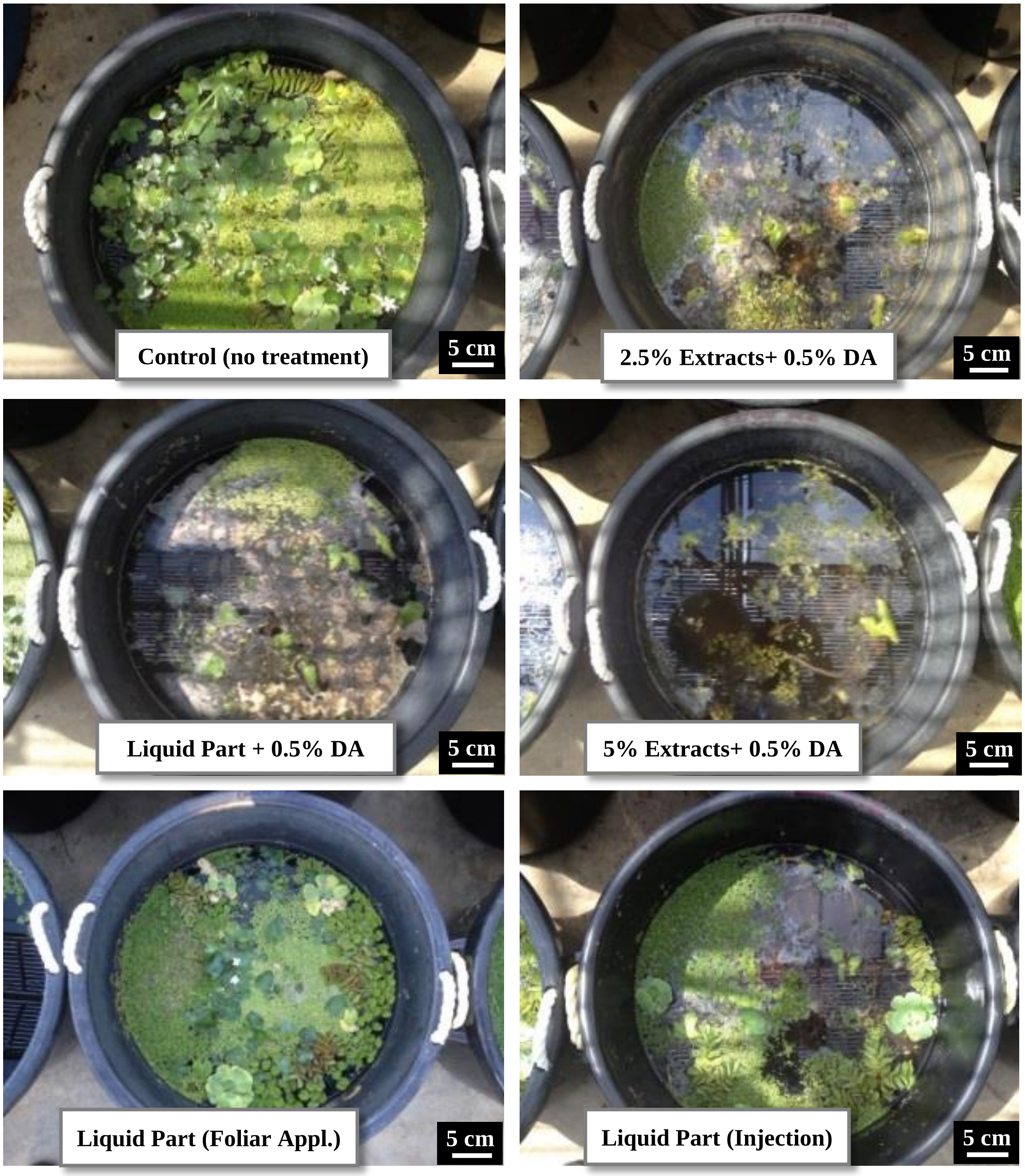

Supplementary Figure S26 The photos showing the result of the foliar application of the liquid part and EtOH extracts of crested floating heart (*Nymphoides cristata*) on *N. cristata* plants by the end of 2nd week of the treatments (DA: Dyne-Amic).

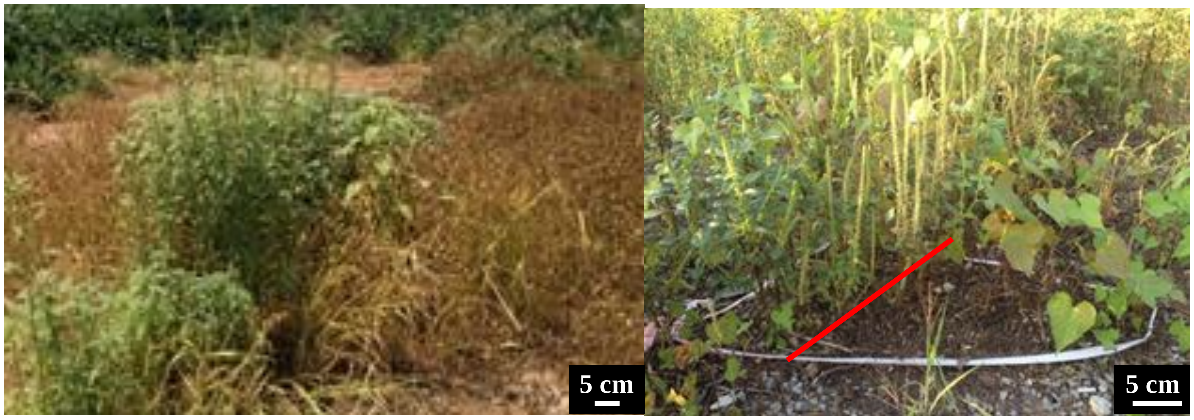

Supplementary Figure S27 Left photo: Glyphosate-resistant Palmer's pigweed (*Amaranthus palmeri* S. Wats.) (Amaranthaceae) next to the soybean field in the southern USA. Right Photos: All plants of *A. palmeri* (on the right of the red line) treated with *A. palmeri* extracts and 1% Tergitol were dead while big-rooted morning glory (*Ipomoea pandurata* (L.) G.F.W. Mey.) still grew.

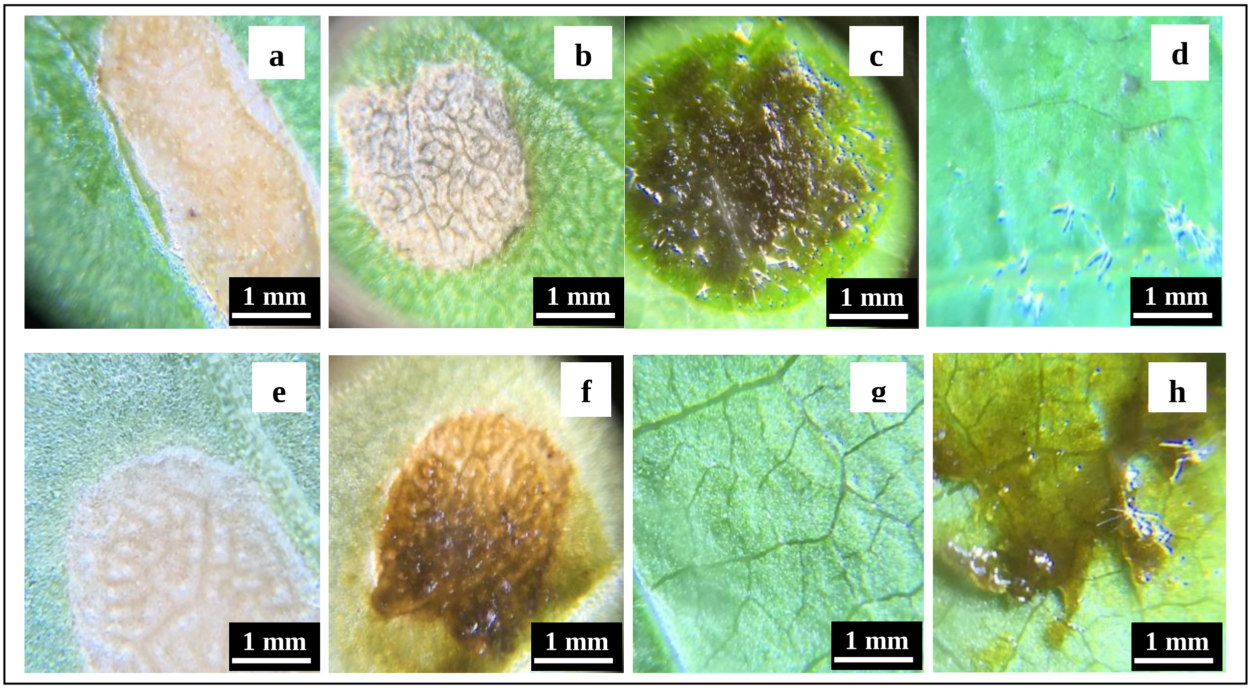

Supplementary Figure S28 The tissues in the leaf spots of *A. palmeri* treated with 10 µL 10% EtOH extracts of *A. palmeri* by either upper or lower surface applications were totally destroyed within two weeks (upper leaf surface (**a**) and lower leaf surface (**e**) after the upper surface application; upper leaf surface (**b**) and lower leaf surface (**f**) after the lower surface application). *Quercus texana* seedlings were not affected by the EtOH extracts of *A. palmeri* with either upper or lower surface applications (upper leaf surface (**c**) and lower leaf surface (**g**) after the upper surface application; upper leaf surface (**d**) and lower leaf surface (**h**) after the lower surface application).

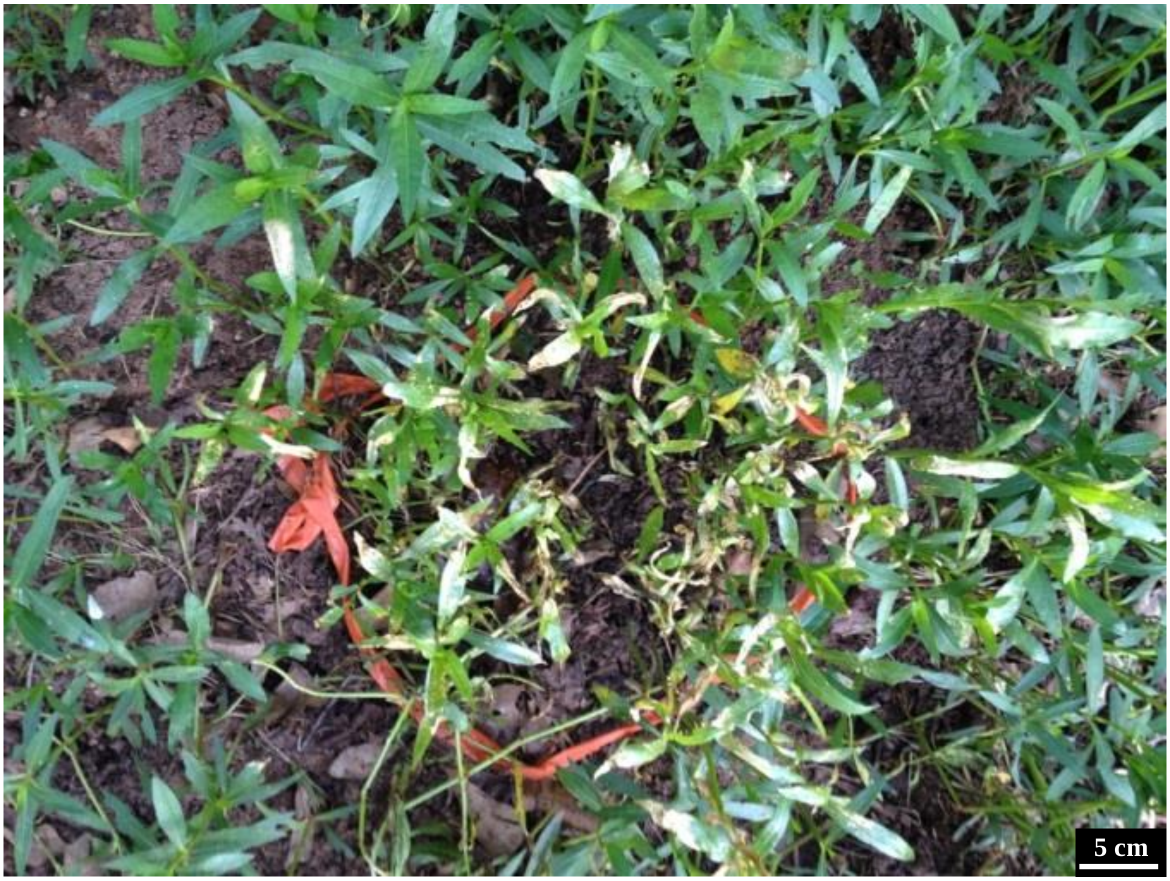

Supplementary Figure S29 The photo shows that alligator weed (*Alternatheria philoxeroides*) plants had significant injury with two plant death five days after treated by 10% EtOH extracts of alligator weed with surfactants (area circled by orange ribbon) in comparison with untreated plants around.

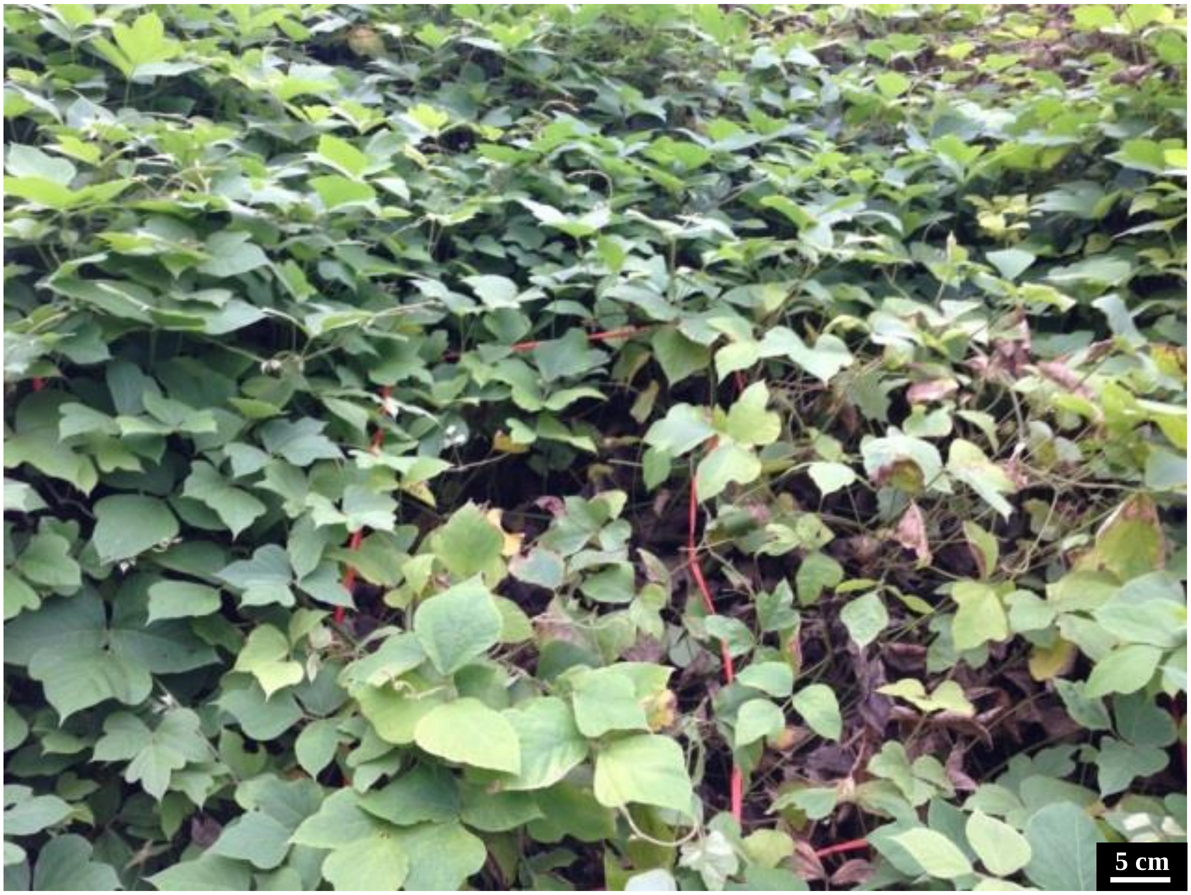

Supplementary Figure S30 The photo shows that EtOH extracts of kudzu (*Pueraria montana*) had significant effects on kudzu plants in three plots (0.5 m^2 each) of the field tests (left plot: control, middle plot: 5% kudzu EtOH extracts, and right plot: 10% kudzu EtOH extracts).

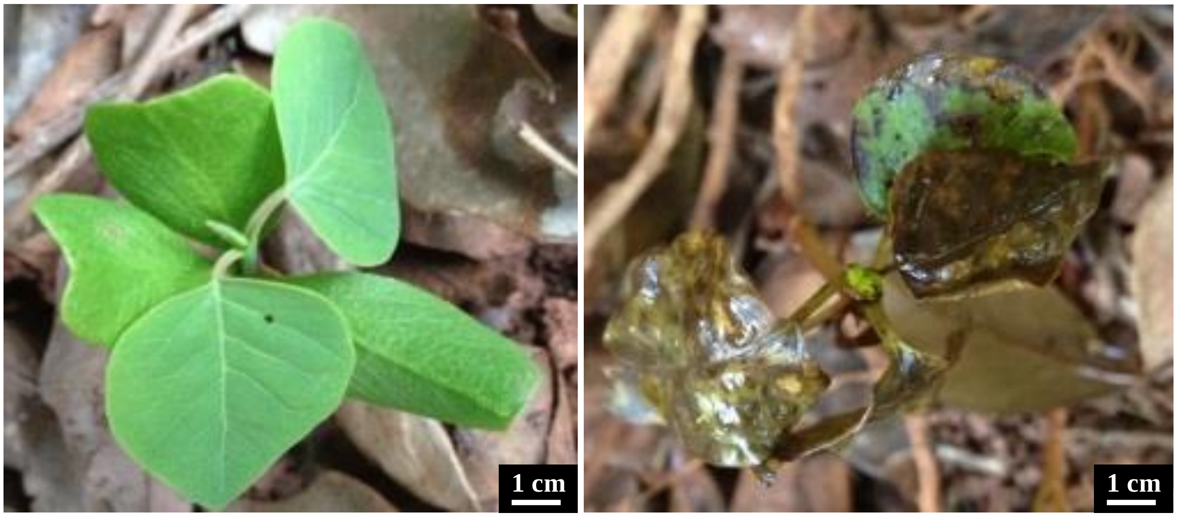

Supplementary Figure S31 A three-week-old seedling of Chinese tallow (*Triadica sebifera*) was significantly injured by foliar application of EtOH extracts of *T. sebifera* stems two days later (right) in comparison with the before treatment (left). The seedling subse-quently died.

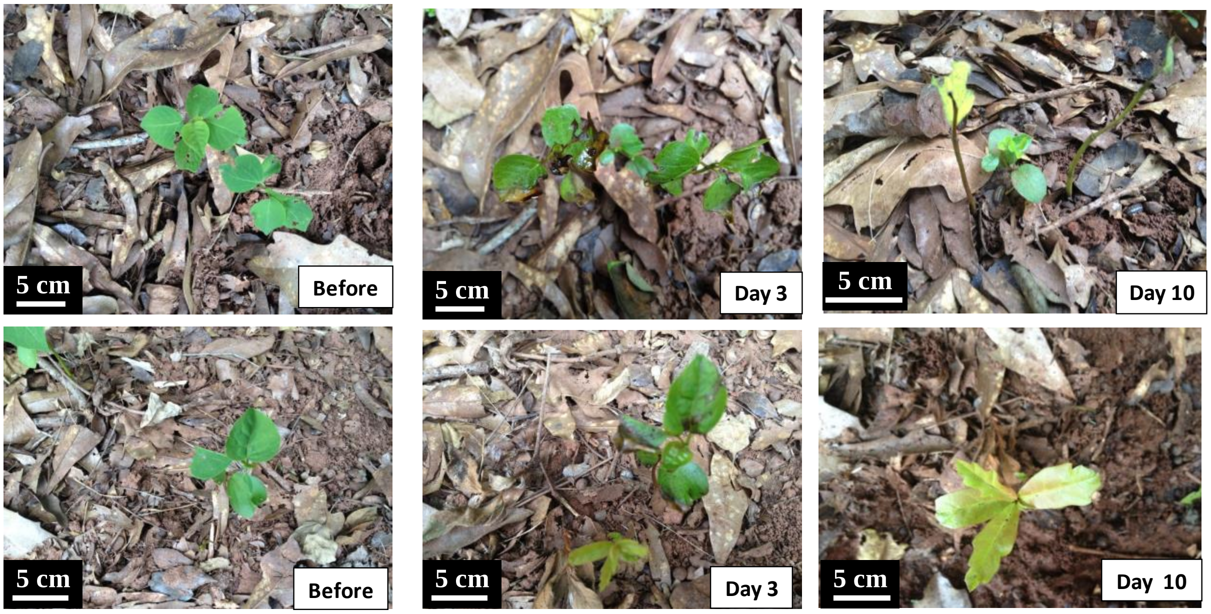

Supplementary Figure S32 The photographs show that the impacts of 10% EtOH extracts of *Triadica sebifera* leaves and stems on seedlings of *T. sebifera*, *Ligustrum sinense*, and *Quercus texana*. All treated 3-week-old *T. sebifera* seedlings were wilted and turned yellow within 10 days (right on the upper row) in comparison with before (left on the upper row). However, the *T. sebifera* extracts did not cause any damage in either *L. sinense* (right on the upper row) or *Q. texana* (right on the bottom row).

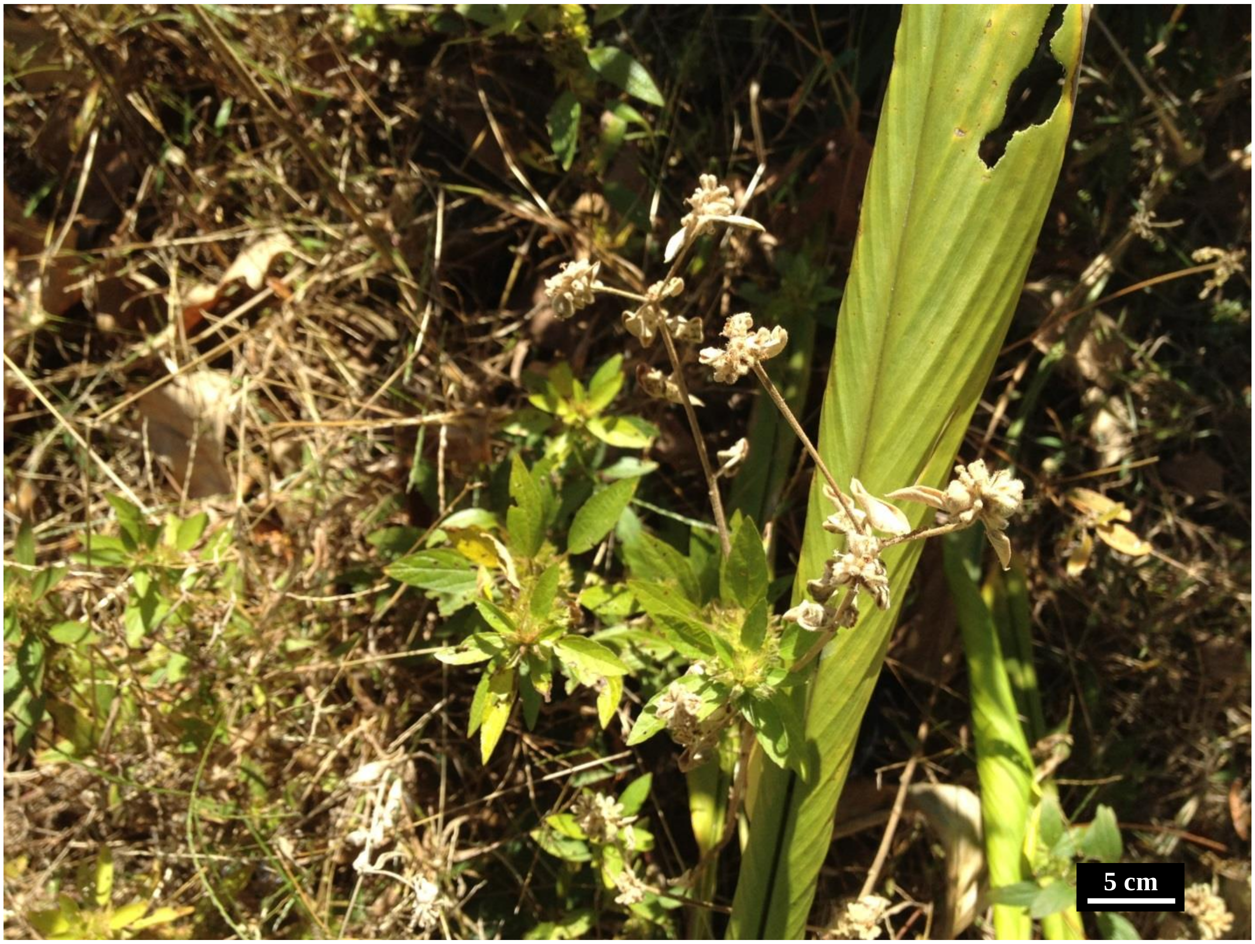

Supplementary Figure S33 The extracts of *C. capitatus* var. *lindheimeri* inhibited and eliminated the producing species but did not have any inhibitory activity against the monocots bermudagrass (*Cynodon dactylon* (L.) Pers.) or turmeric (*Curcuma longa* L.) and even three-seeded mercury (*Acalypha rhomboidea* Raf.), of the same Eurphorbiaceae family in the same treatment.

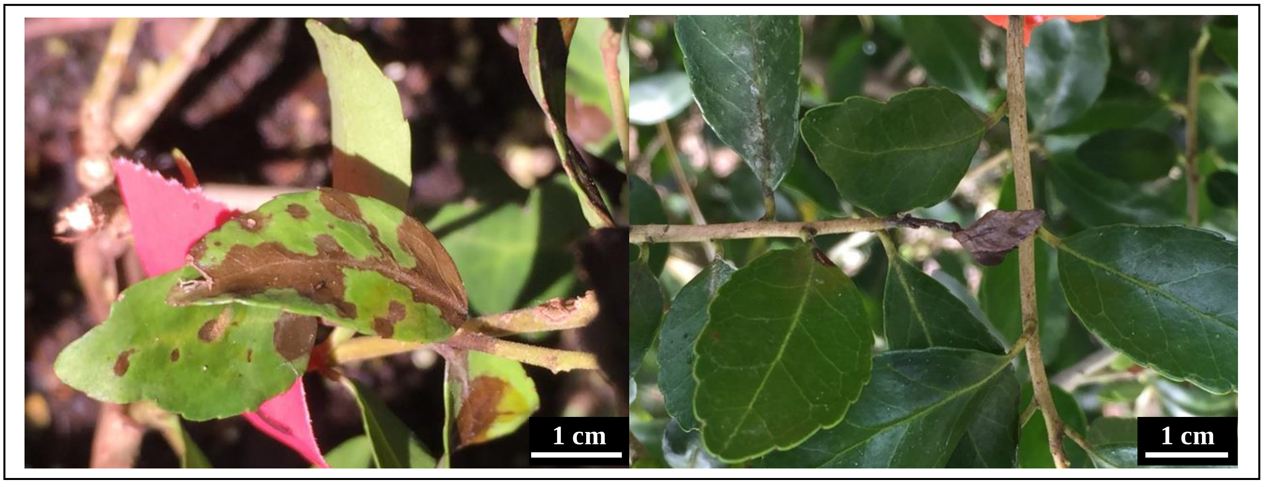

Supplementary Figure S34 The t-BuOH fraction of the EtOH extracts of yaupon (*Ilex vomitoria* Sol. Ex Alton.) showed toxicity against both young plant in greenhouse (left) or mature plant in the field (right). The left photo showed leaf injury 3 days after the application of three drops of 10 µL t-BuOH fraction. The right photo showed the terminal bud and young shoot were killed by foliar application of t-BuOH fraction within 9 days.

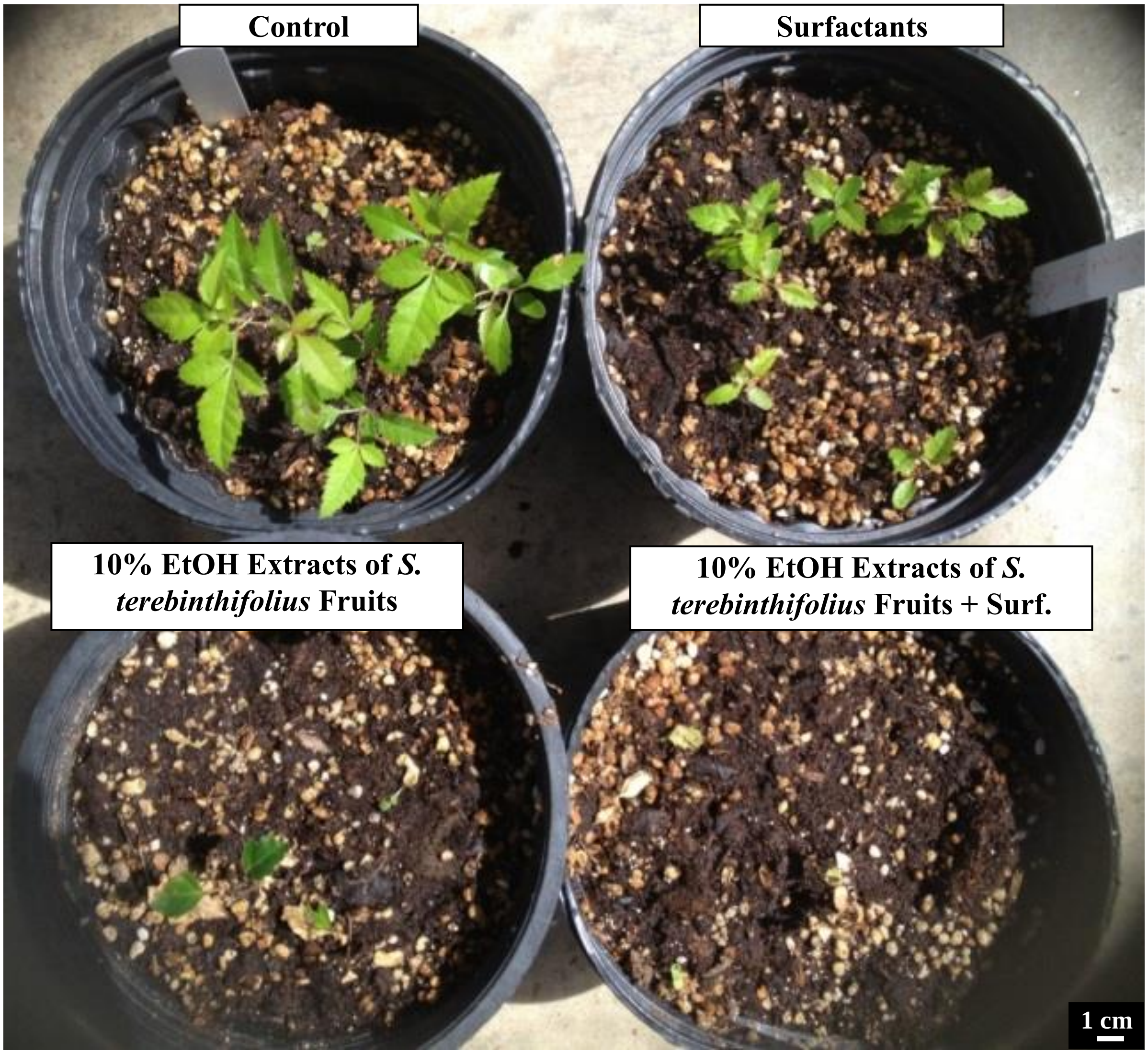

Supplementary Figure S35 The photograph shows that 3-week-old Brazilian pepper tree (*Schinus terebinthifolius*) seedlings had significant damage after the first treatment with 10% *S. terebinthifolius* extracts or *S. terebinthifolius* extracts with surfactants in comparison with those in control and surfactants treatment. By the end of the 4th week, all six *S. terebinthifolius* seedlings were dead following the *S. terebinthifolius* extracts with surfactants treatment and five seedlings were dead and one had dead apical bud and young leaves after treated by the *S. terebinthifolius* extracts.

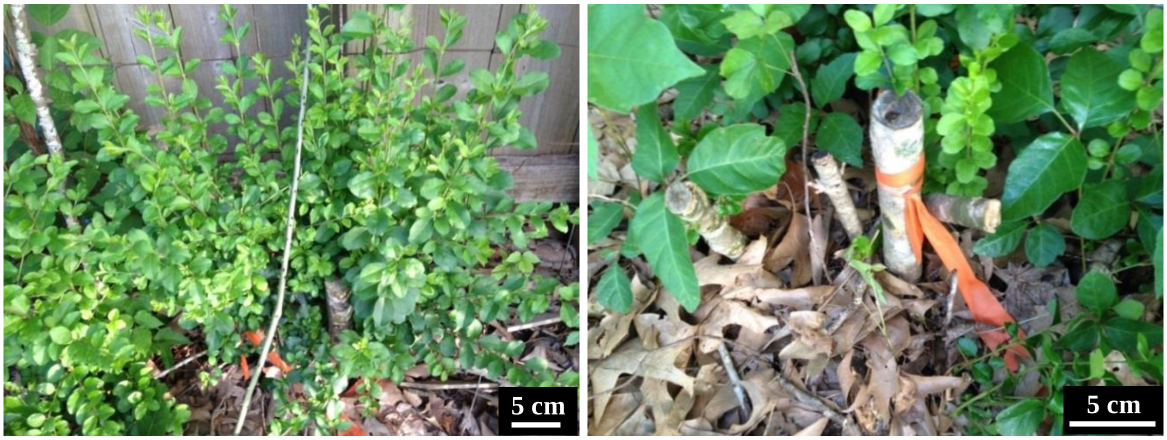

Supplementary Figure S36 The photos show that Chinese privet (*Ligustrum sinense* Lour.) responded to the 10% EtOH extracts of *L. sinense* fruits two months after the cut stump treatments (left: the growth of coppice from the stump in control, right: no coppice growth from the treated stumps).

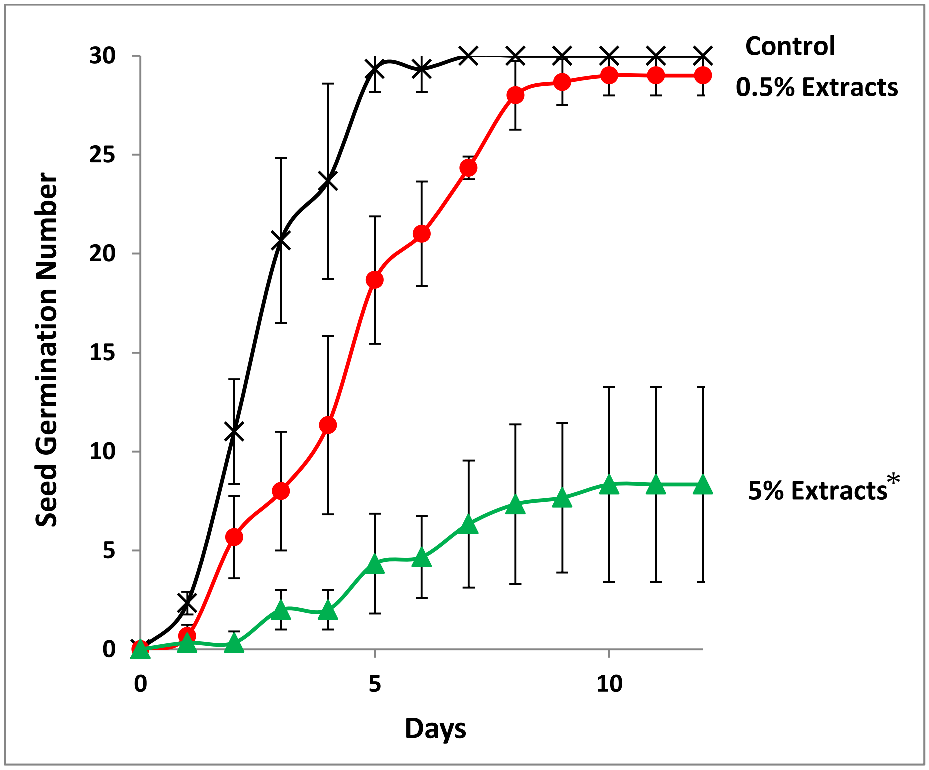

Supplementary Figure S37 The germination of *Phaseolus vulgaris* is affected by its EtOH extracts. Soaking of the *P. vulgaris* beans in 0.5% extracts can significantly delay the germination, while over 70% of beans did not germinate after the treatment in 5% extracts. Asterisks denote significant differences between treatment and control values at the end of the 12th week ($P < 0.05$).

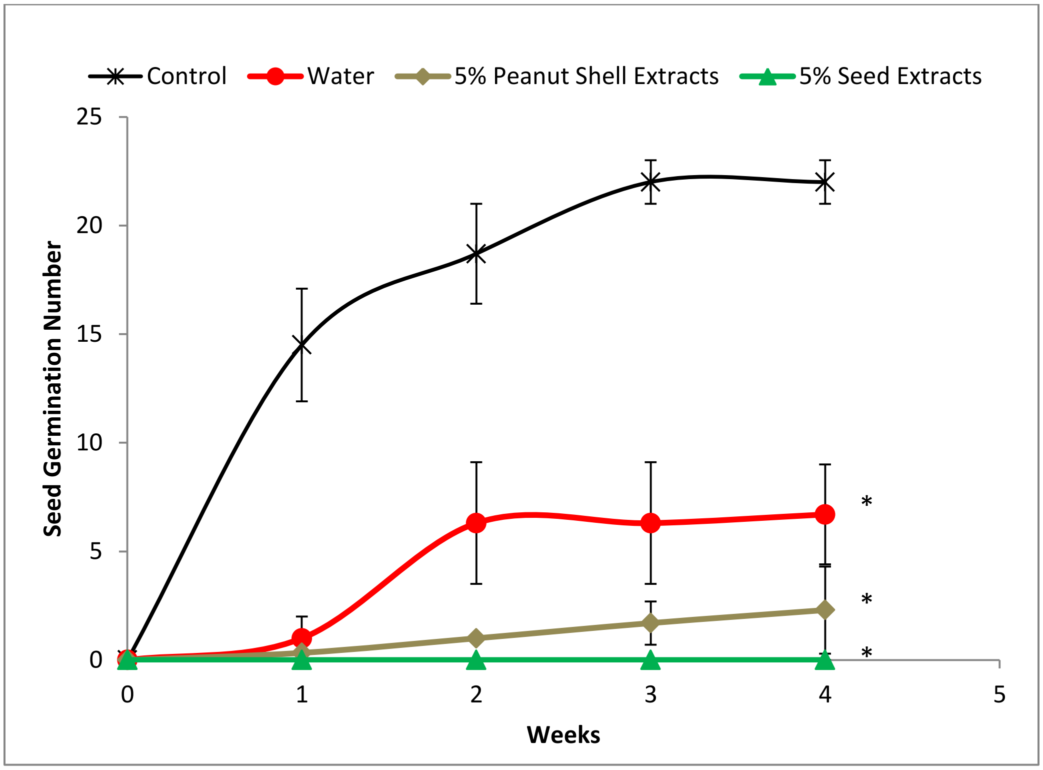

Supplementary Figure S38 The impact of peanut (*Arachis hypogaea*) extracts on the seed germination of *A. hypogaea*. Asterisks denote significant differences between treatment and control values at the end of four weeks ($P < 0.05$).

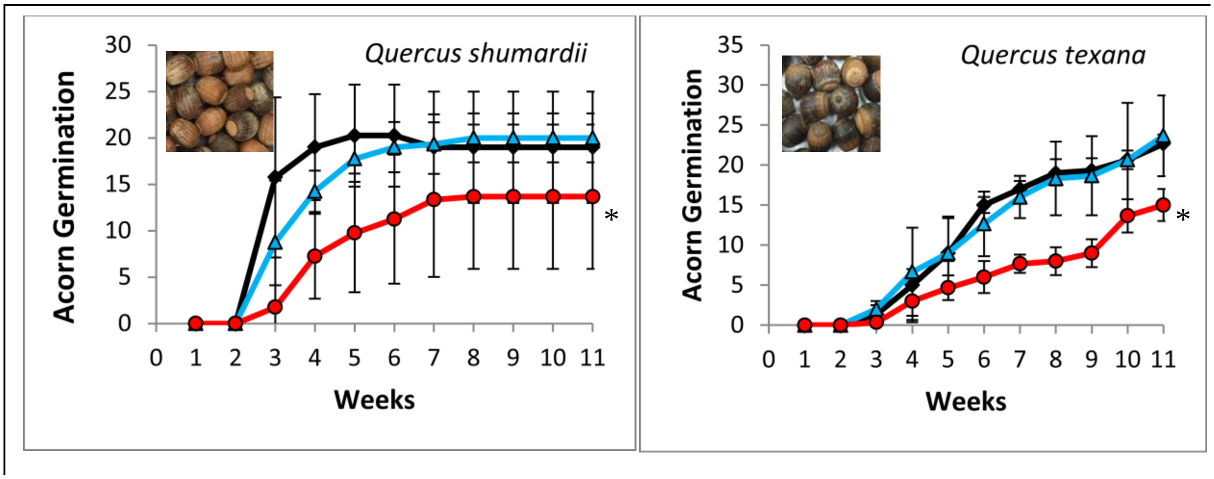

Supplementary Figure S39 The acorn germination of *Quercus texana* and *Q. shumardii* can be greatly inhibited by the EtOH extracts of *Q. shumardii* acorns (black line: control; blue line: 0.5% extracts; and red line: 5% extracts). Asterisks denote significant differences between treatment and control values at the end of the 11th week ($P < 0.05$).

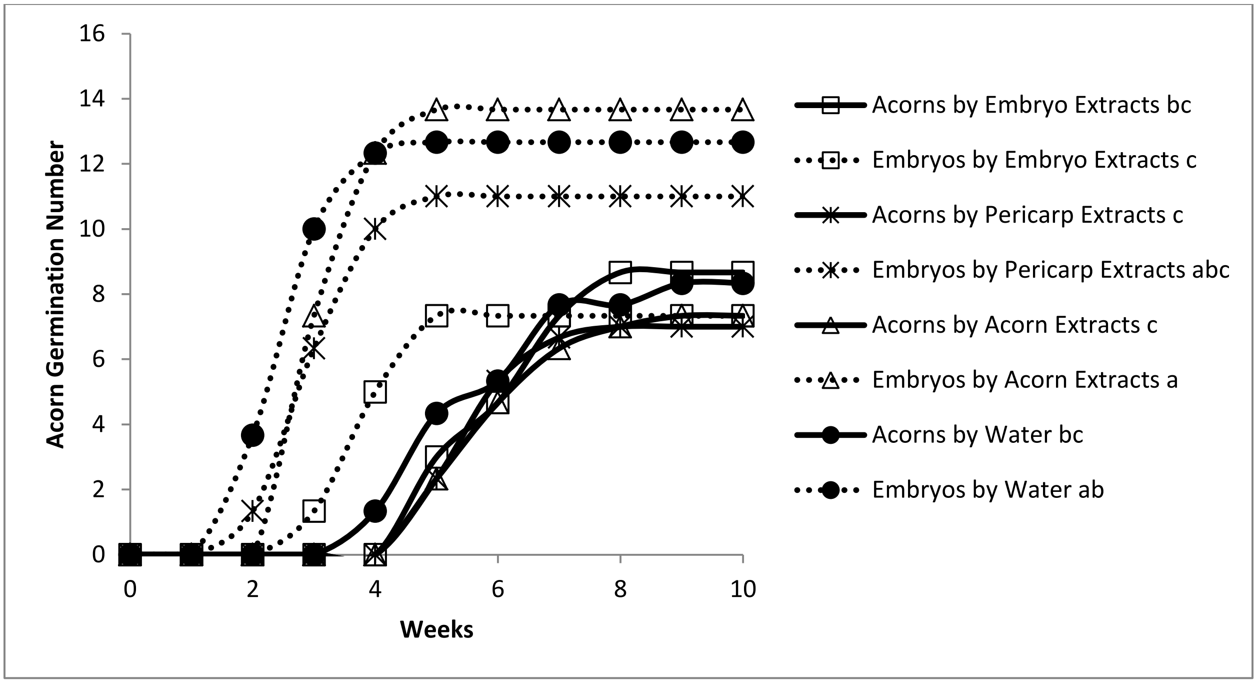

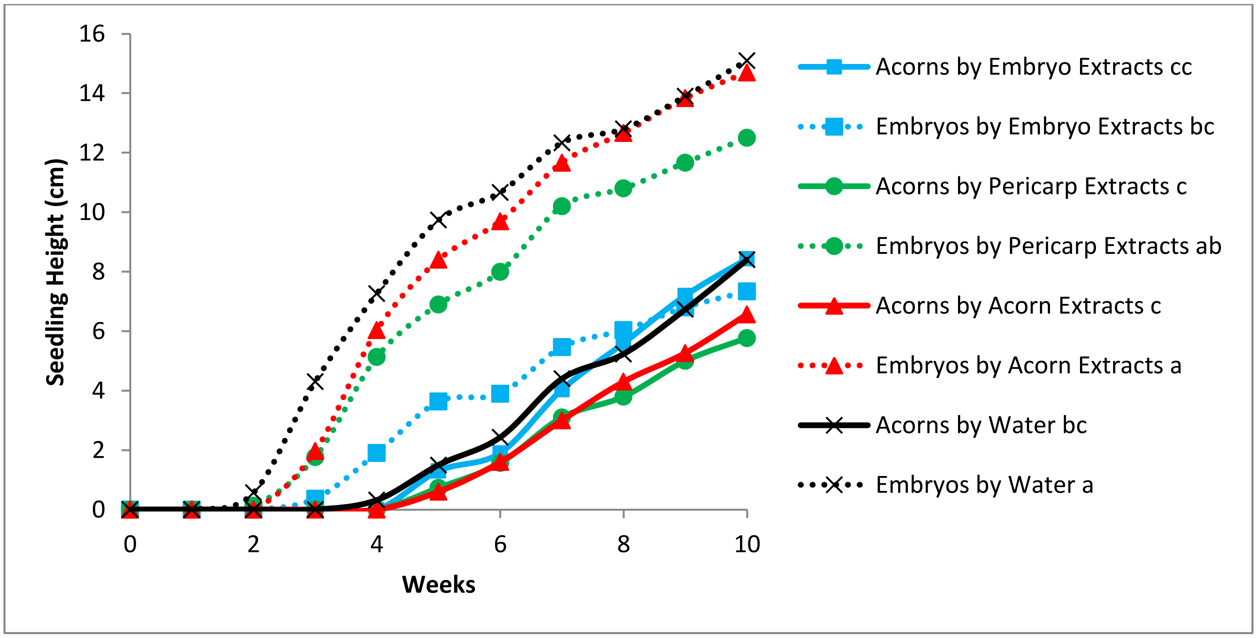

Supplementary Figure S40 By the end of 10 weeks of planting, the germination rates of the acorn embryos of Nutall oak (*Qercus texana*) had no significant difference among the treatments of water, extracts of *Q. texana* whole acorns, and extracts of *Q. texana* acorn pericarps but all were significantly higher than those treated with extracts of *Q. texana* acorn embryos or whole acorns of *Q. texana* in any treartments (upper). The height growth of seedlings in different treatments exhibited a similar pattern (lower). Significant differences between the treatments at the end of 10 weeks are indicated by different letters ($P < 0.05$).

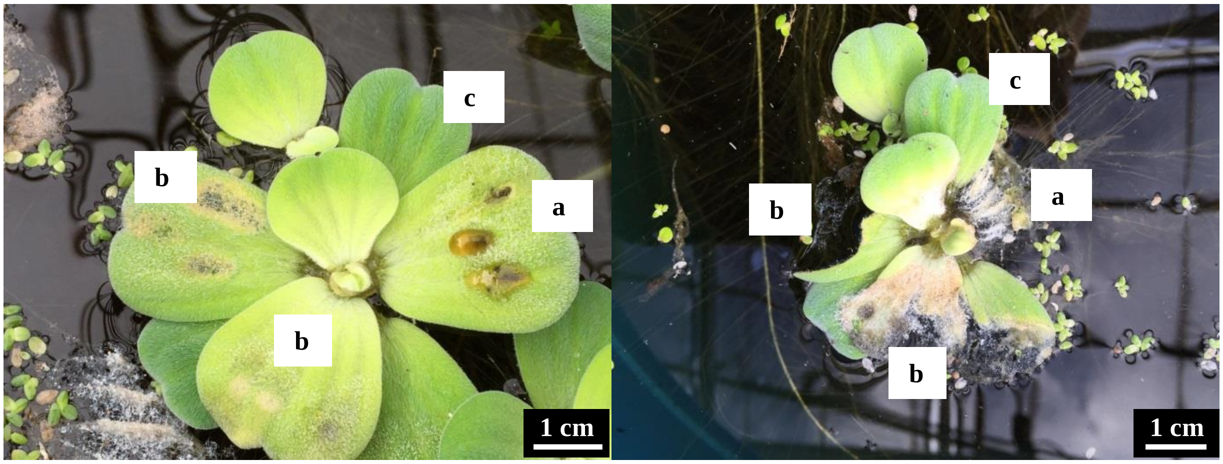

Supplementary Figure S41 The photo shows that water lettuce (*Pistia.stratiotes* L.) treated by 10 µL 10% EtOH extracts of *P. stratiotes* on both upper (**a**) and lower leaf surface (**b**) turned into brown in color after three days (left) and 14 days (right) of the treatment (**c**: control without any treatment).

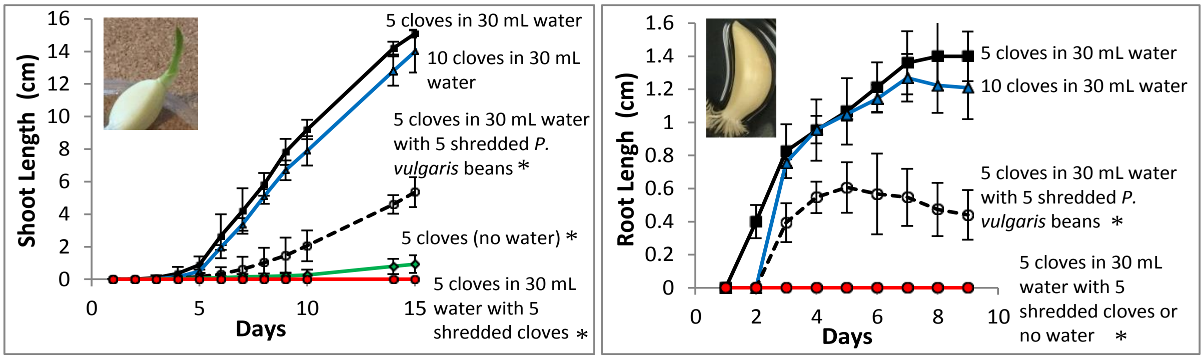

Supplementary Figure S42 The growth of *Allium sativum* can be totally inhibited by soaking in solution of shredded garlic. No root or shoot of *A. sativum* cloves were developed in the water with shredded cloves. Asterisks denote significant differences between treatment and control values on the last experimental day ($P < 0.05$).

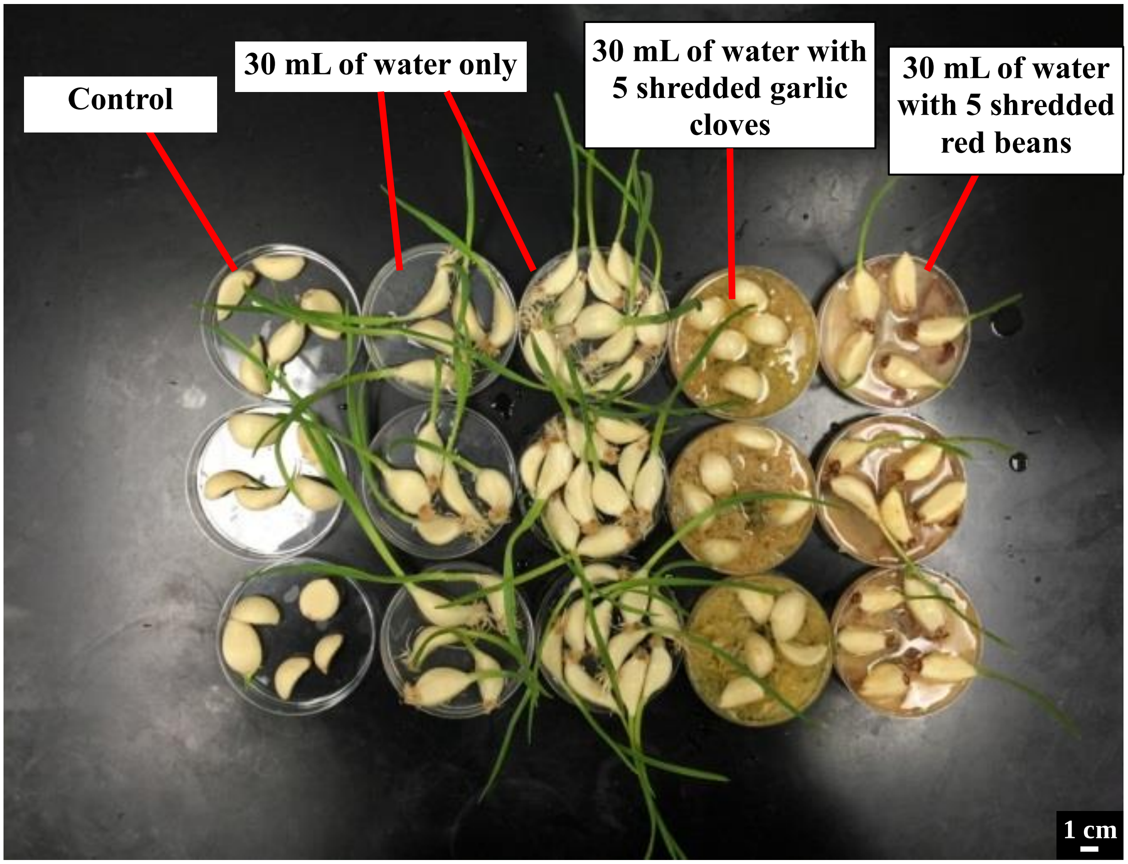

Supplementary Figure S43 The photo shows that growth of garlic (*Allium sativum*) was totally inhibited by the shredded cloves of *A. sativum.*

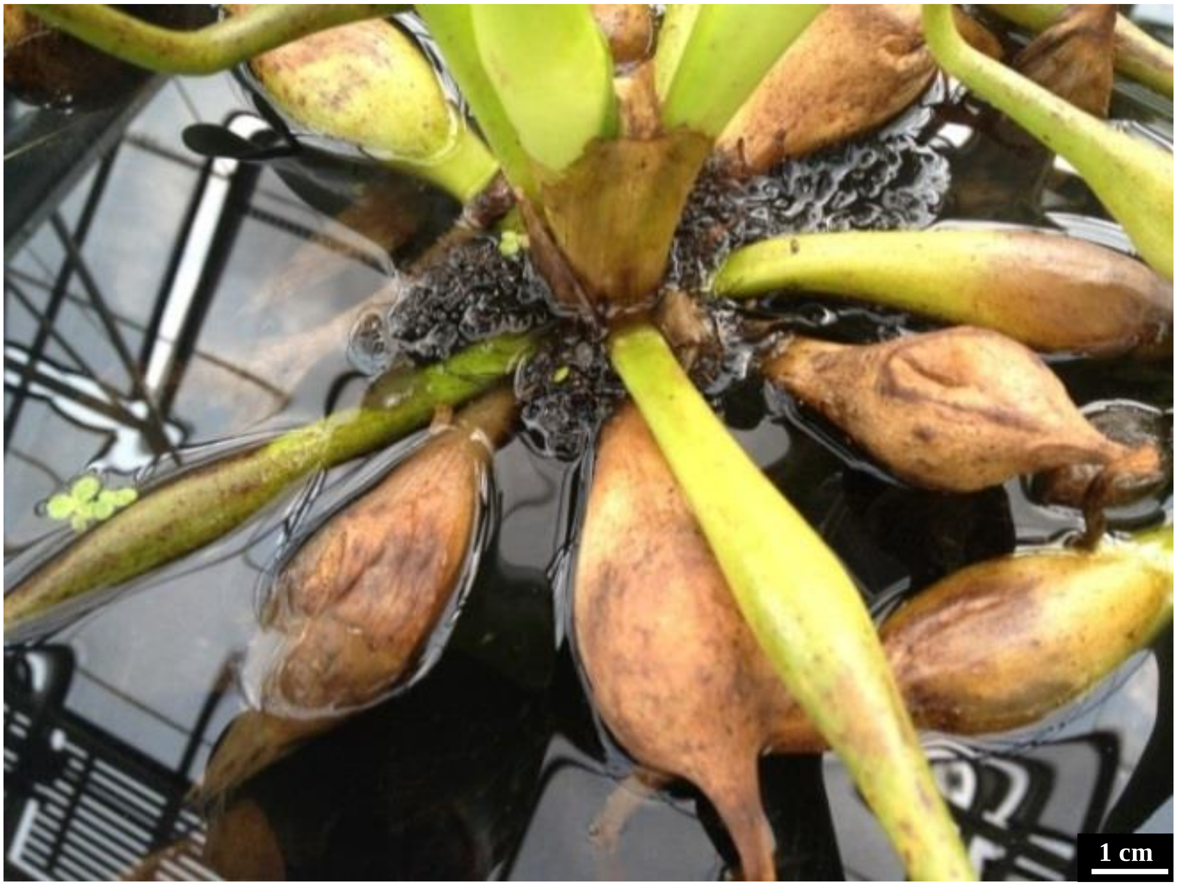

Supplementary Figure S44 The photo shows that most leaves and spongy and bulbous stalks of water hyacinth (*Elchornia crossipes*) were dead three weeks after the foliar treatment of 10% EtOH extracts of water hyacinth with surfactants.

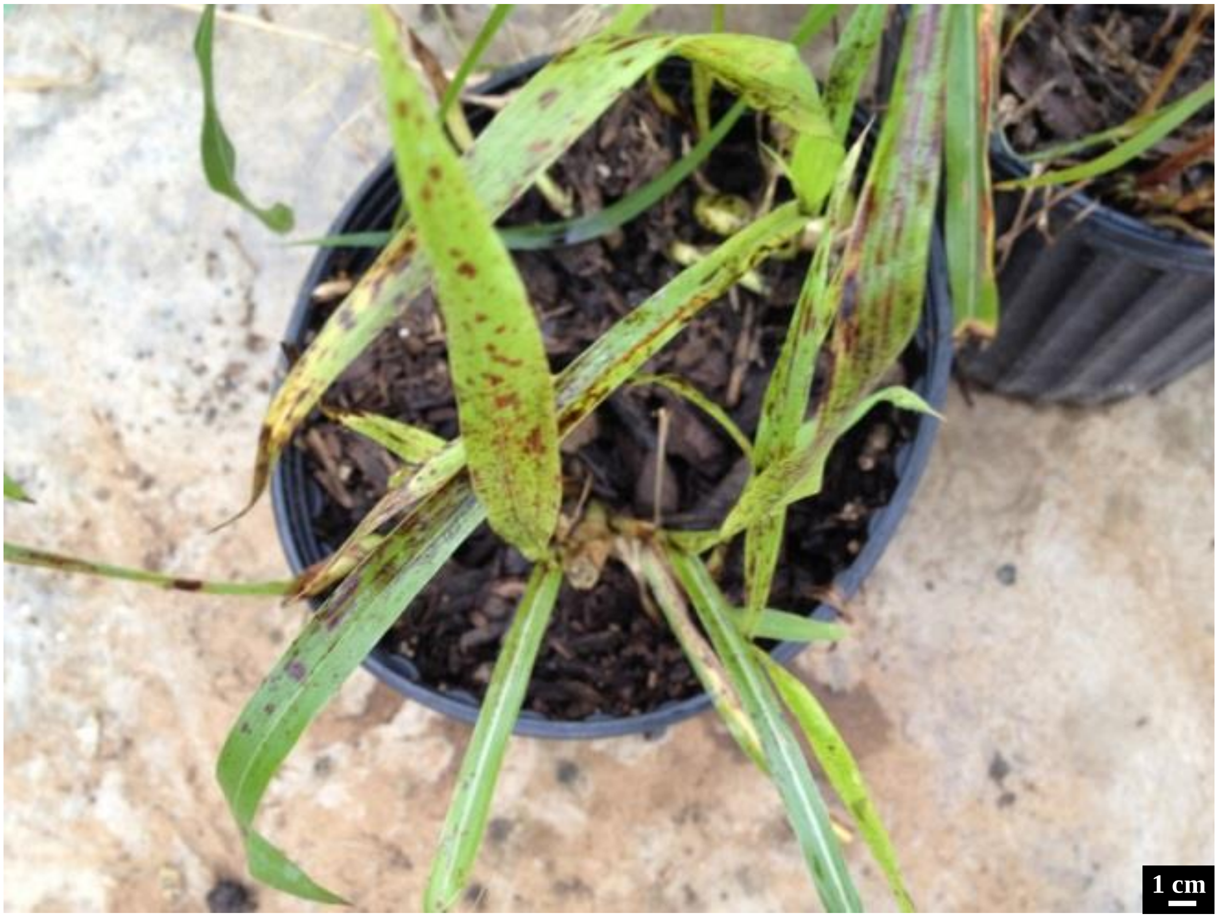

Supplementary Figure S45 Johnsongrass (*Sorghum halepense*) was injured by spray of the *S. halepense* extracts.

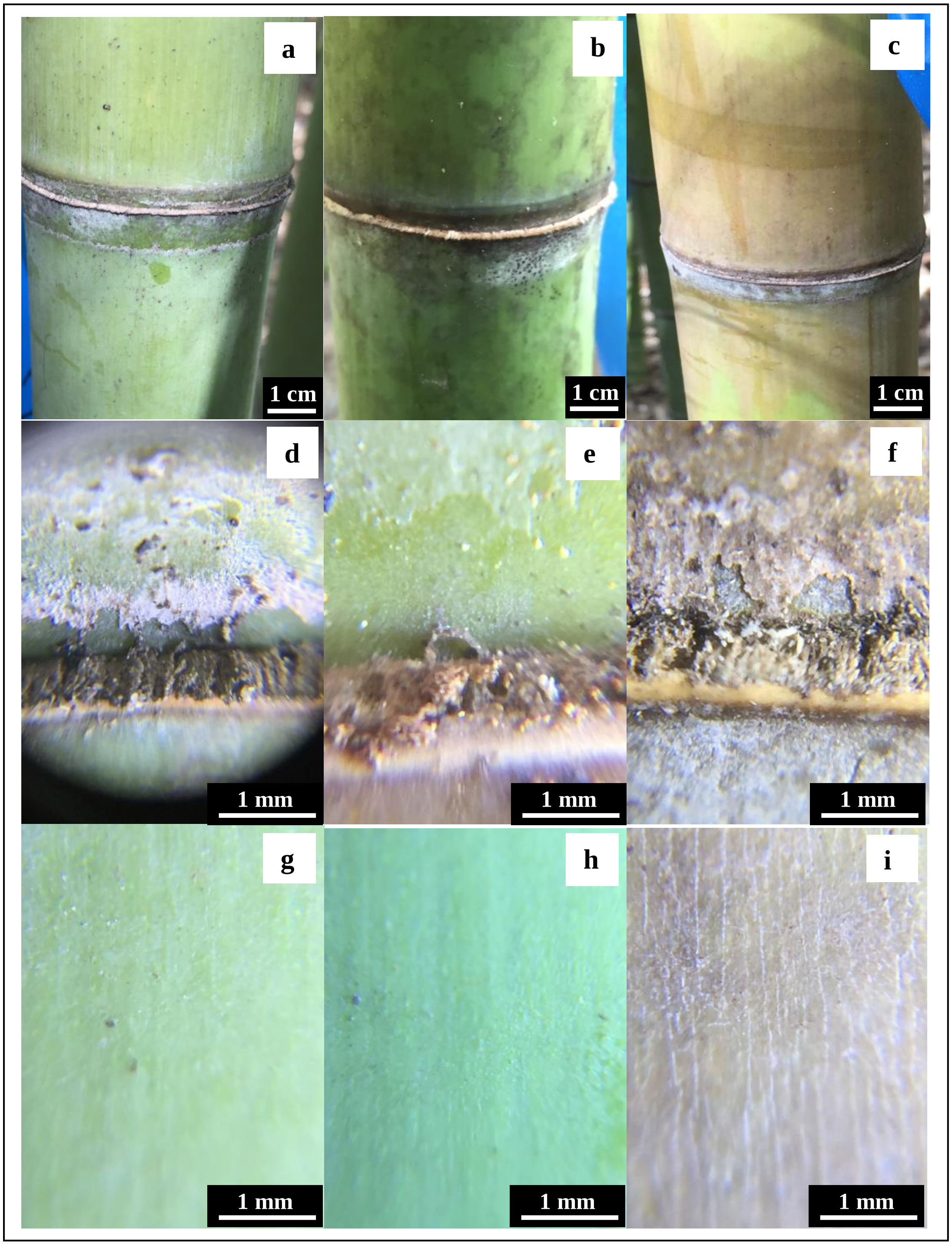

Supplementary Figure S46 The photos show the morphological changes of the first node below the culm cut of golden bamboo (*Phyllostachys aurea*) a week after the cut stump (culm) treatment at 100 cm high above ground: control with culm cut only (**a**, **d**, and **g**), application of 5 mL 10% EtOH extracts of giant reed (*Arundo donax*) on the culm cut (**b**, **e**, and **h**) on the culm cut, and application of 5 mL 10% EtOH extracts of *P. aurea* (**c**, **f**, and **i**) on the culm cut.

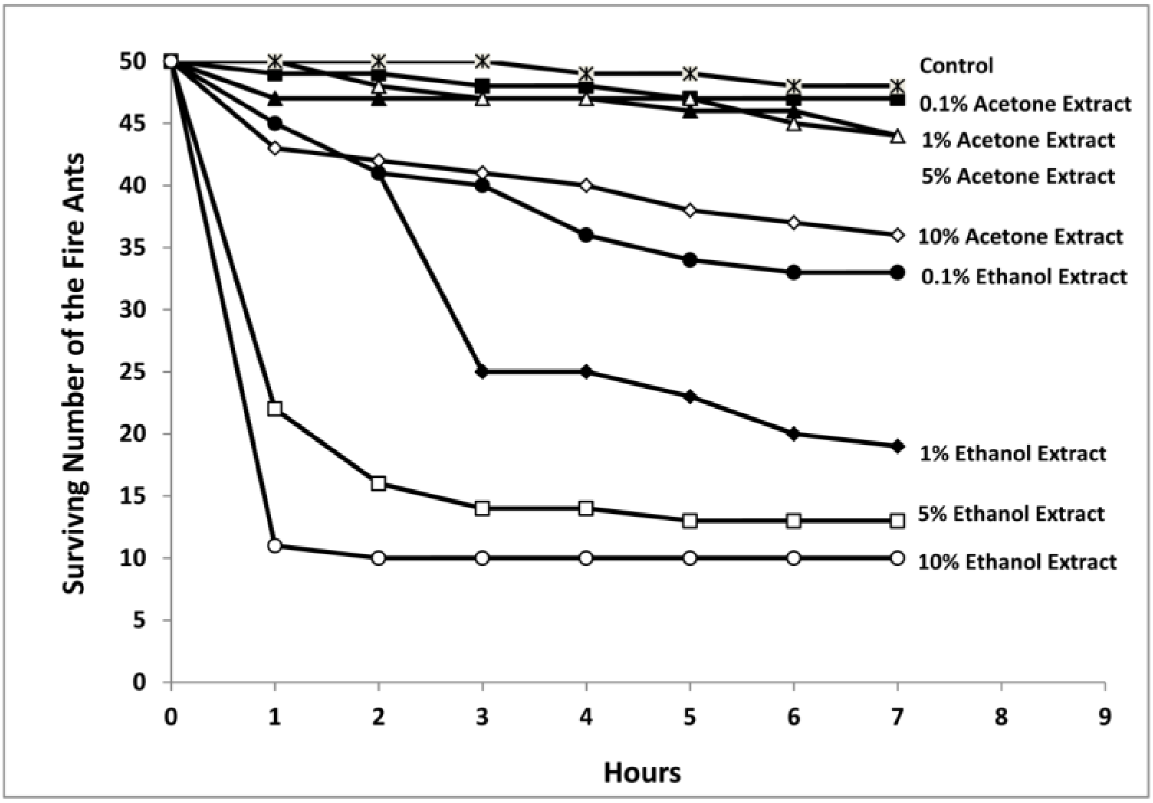

Supplementary Figure S47 The diagram shows the impacts of acetone extracts and EtOH extracts of the red imported fire ant (*Solenopsis invicta* Buren) on the workers of *S. invicta*. The EtOH extracts showed more significant toxicity against *S. invicta* than the acetone extract. During the 7 h of contact toxicity bioassays, an average of approximately 60%, 70%, or 80% of the 50 *S. invicta* in contact with 1%, 5%, or 10% EtOH extracts were dead, respectively.

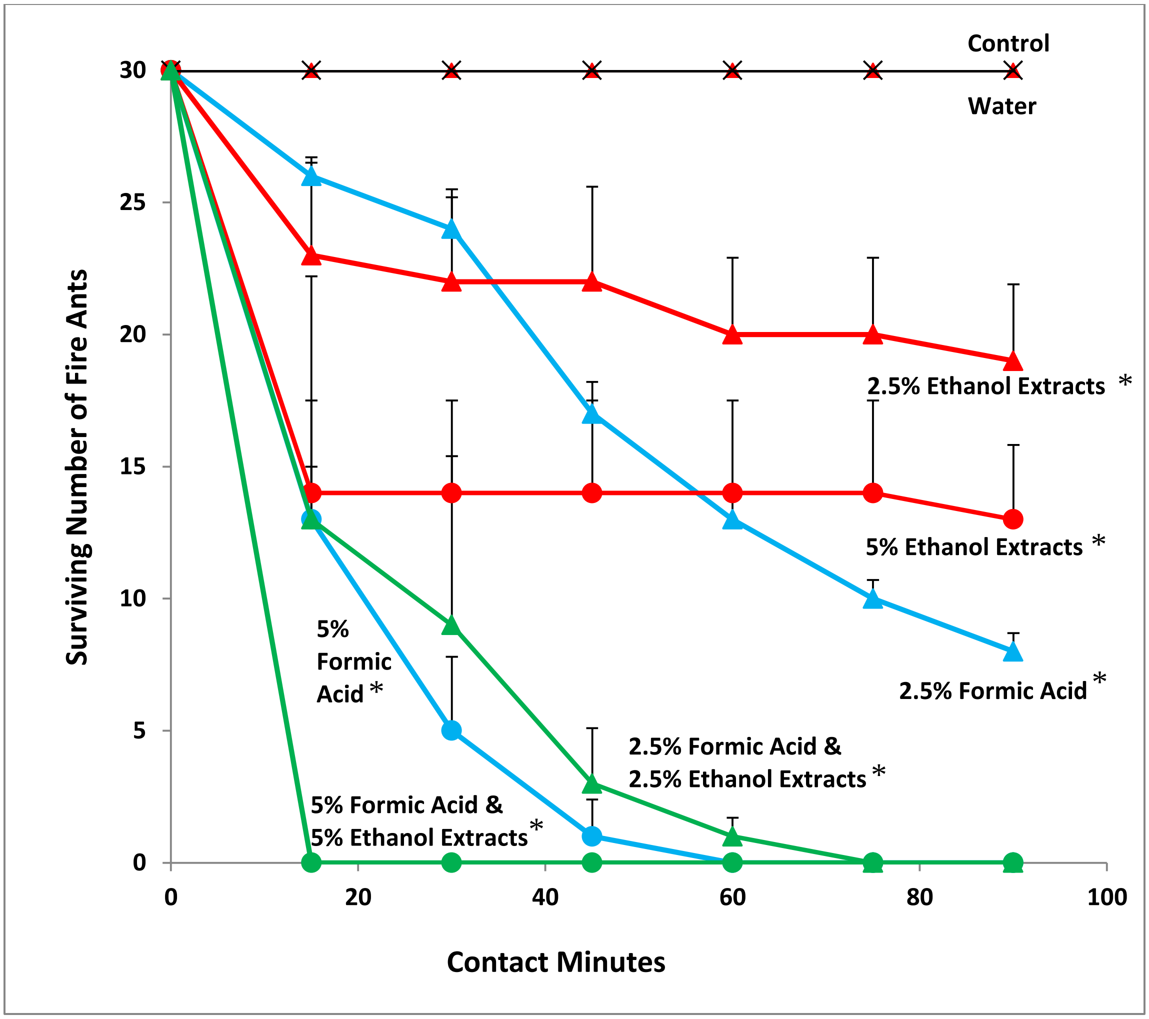

Supplementary Figure S48 The diagram shows the impacts of EtOH extracts of the red imported fire ants (*Solenopsis invicta*) and formic acid on the heathy workers of *S. invicta*. During the 90 min of contact toxicity bioassays, the combined applications of EtOH extracts and formic acid had more significant effects than the use of either EtOH extracts or formic acid alone (with bars presenting standard deviations). Asterisks denote significant differences between treatment and control values at the end of 90 minutes ($P < 0.05$).

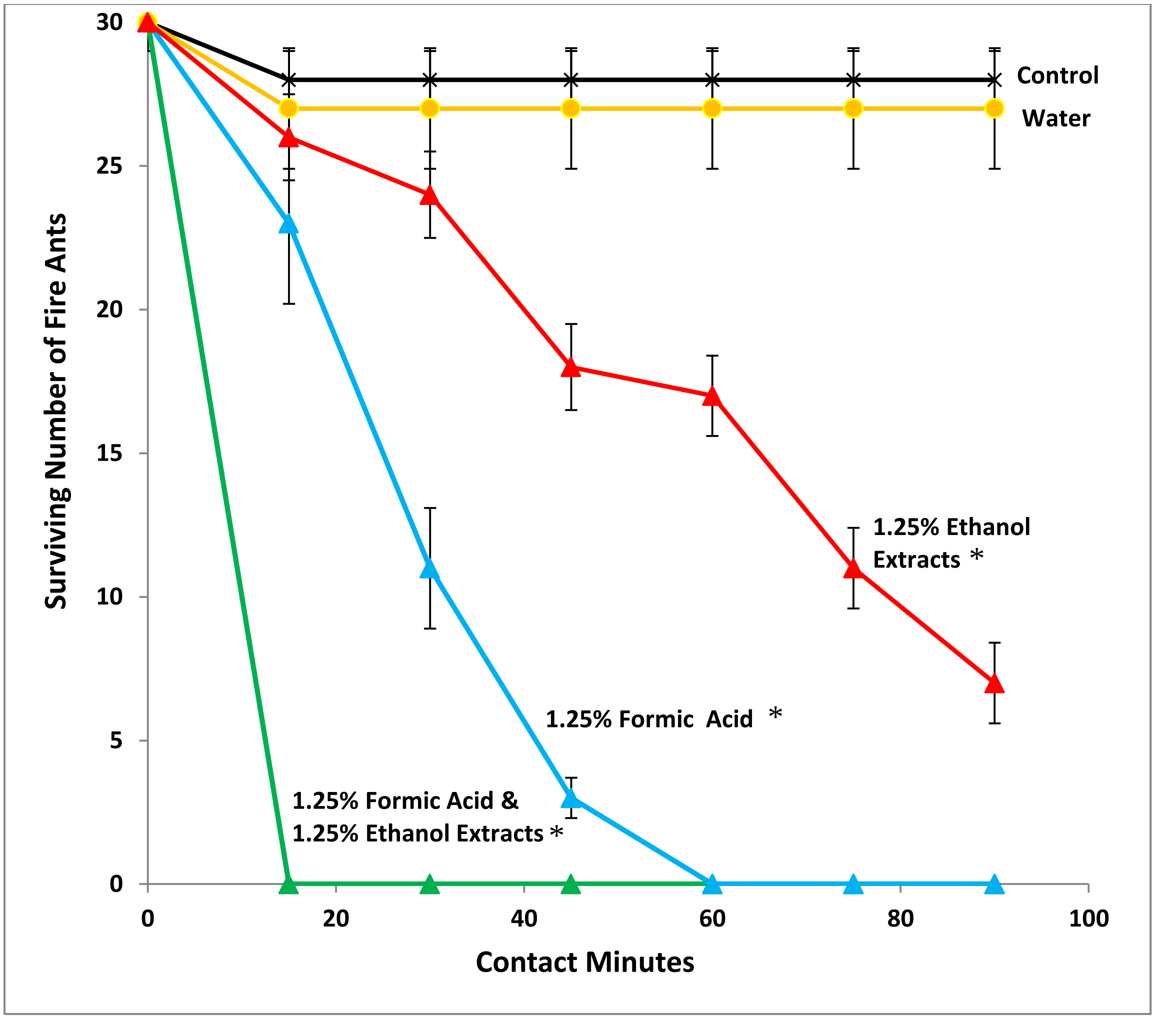

Supplementary Figure S49 The diagram shows the impacts of EtOH extracts of the red imported fire ant (*Solenopsis invicta* Buren) and formic acid on the injured workers of *S. invicta*. During the 90 min of contact toxicity bioassays, the combined applications of EtOH extracts and formic acid had more significant effects than the use of either EtOH extracts or formic acid alone (with bars presenting standard deviations). Asterisks denote significant differences between treatment and control values at the end of 90 minutes ($P < 0.05$).

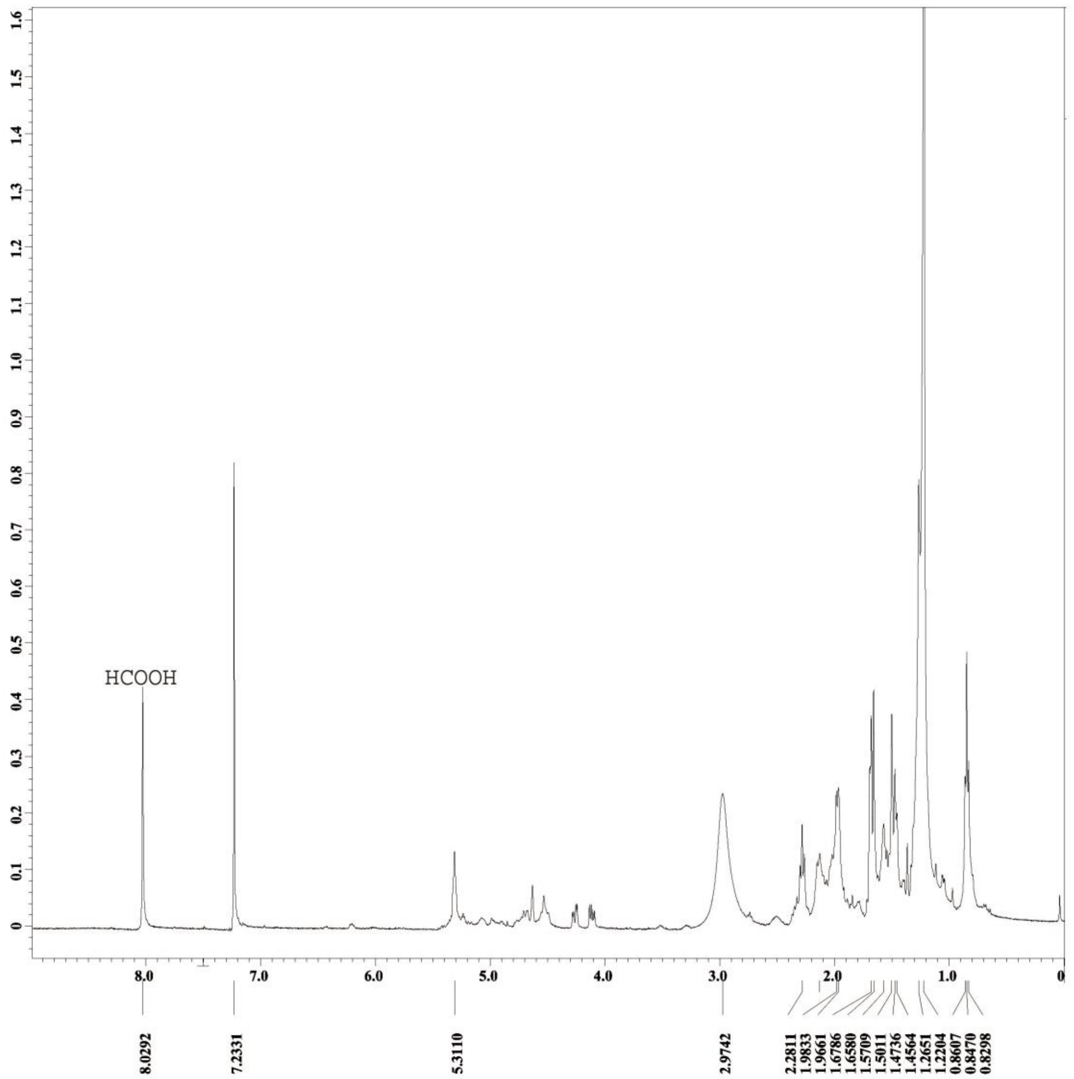

Supplementary Figure S50 The diagram shows the occurrence of formic acid (HCOOH) in chroroform ($CHCl_3$) extracts of the subterranean termite (*Reticulitermes flavipes* (Kollar)) by NMR analysis. ^{1}H-NMR experiments were performed on a JEOL ECS 400 spectrometer, with spectroscopic data referenced to the solvent used. According to standard formic acid ^{1}H-NMR spectrum, the unique singlet of the aldehyde proton should appear at δ_H 8.03.

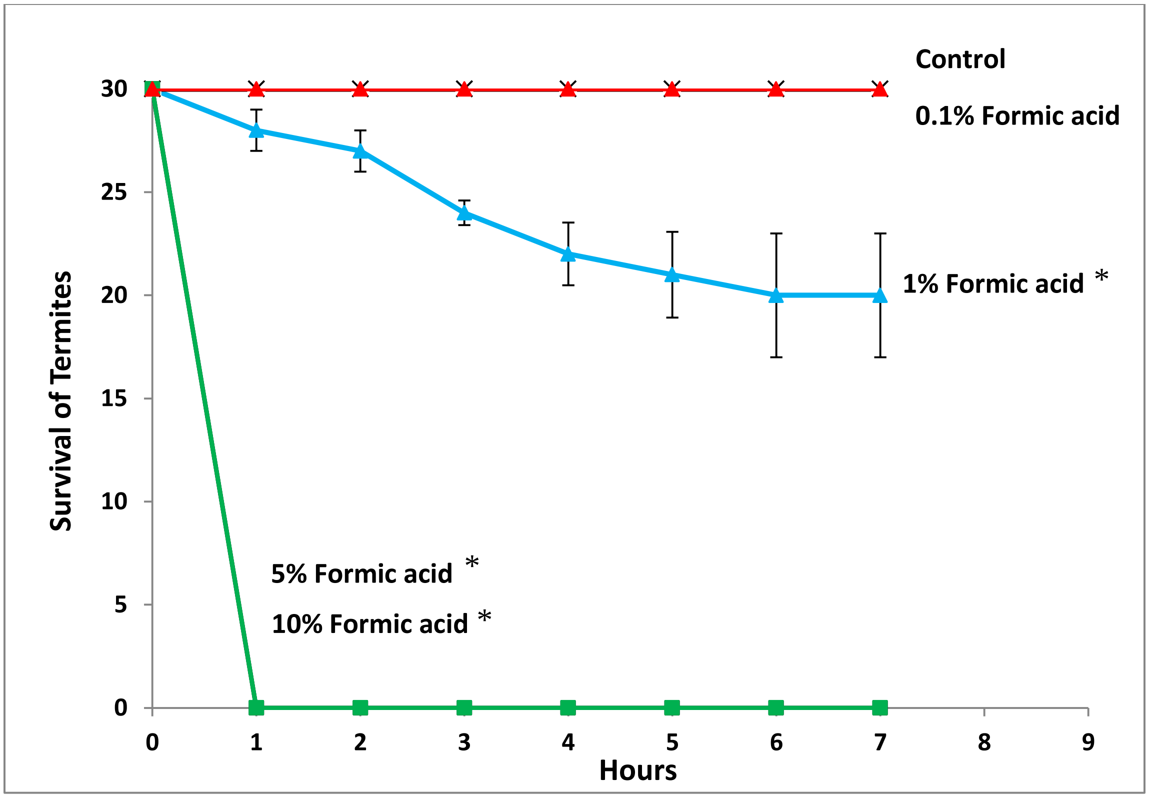

*

Supplementary Figure S51 The diagram shows the impacts of formic acid on the subterranean termite (*Reticulitermes flavipes* (Kollar)). Formic acid at higher concentrations (5 or 10%) killed all termites (including workers, soldiers, and reproductive) during the first hour of contact experiment. More than 40% of the termites were killed by the 1% formic acid during the 7 h of contact toxicity bioassays. However, formic acid at 0.1% concentration had no impact on the termites (with bars presenting standard deviations). Asterisks denote significant differences between treatment and control values at the end of seven h ($P < 0.05$).

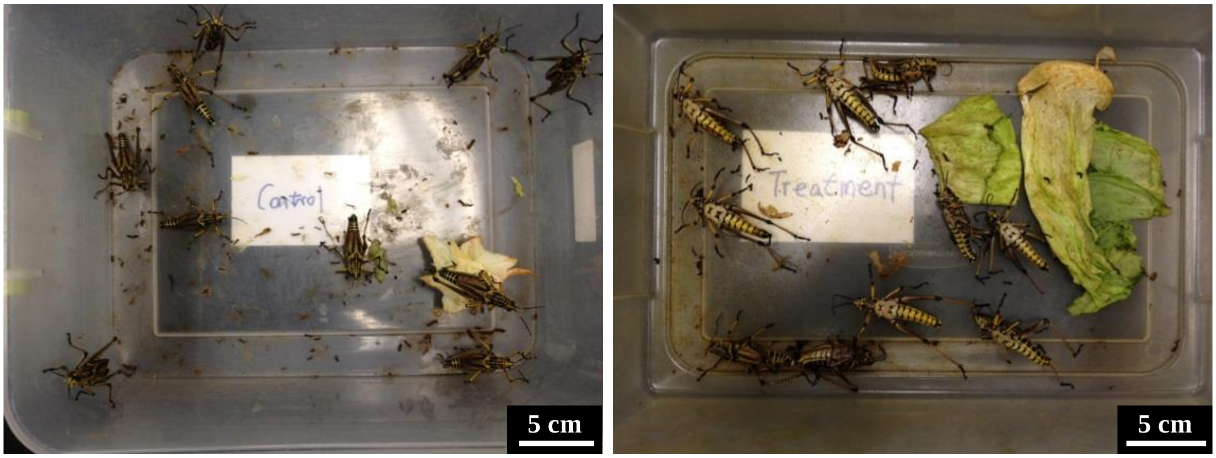

Supplementary Figure S52 The upper photo shows that the 10 grasshoppers in control were still active on the second day of the experiment. The lower photo shows that all 10 grasshopper (*Schistocerca americana* (Drury)) were killed by its 10% EtOH extracts within 24 h after the treatments.

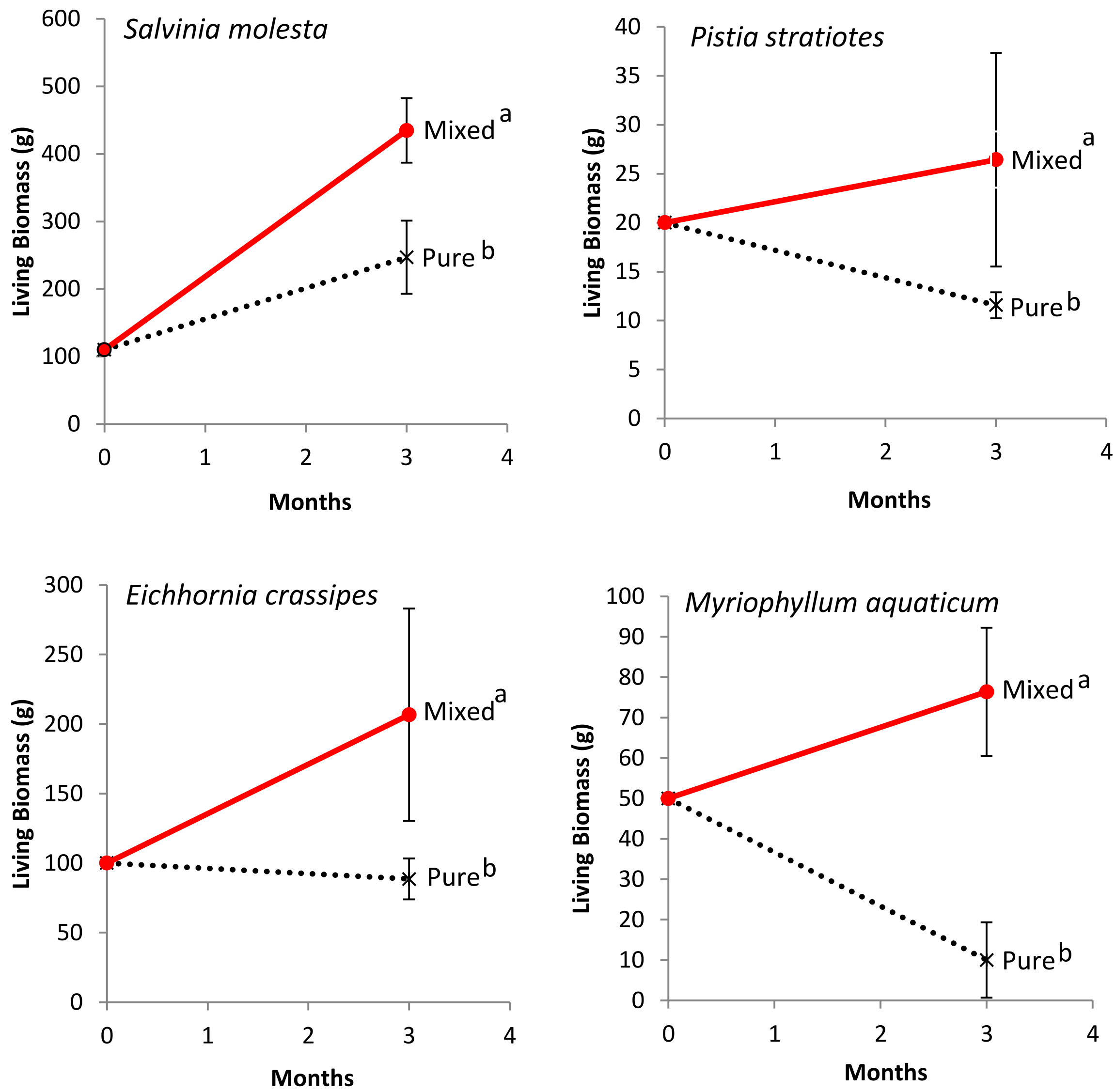

Supplementary Figure S53 Living biomass growth of *Salvinia molesta, Pista stratiotes, Eichhornia crassipes,* and *Myriophyllum aquaticum* in pure or mixed culture. Significant differences between the pure and mixture values at the end of third week in the same chart are indicated by different letters (P < 0.05).

Supplementary Table S1 – Experimental species list for the external applications of endocides for mutations

Kingdom	Phyllum/Division	Family	Species	Common Name	Status in North America
Plantae	dicots of Anthophyta (angiosperms)	Cactaceae	*Opuntia ficus-indica* (L.) Mill.	nopal cactus or Indian fig opuntia	crop
Plantae	dicots of Anthophyta (angiosperms)	Brassicaceae	*Brassica oleracea* L.	broccoli	crop
Plantae	dicots of Anthophyta (angiosperms)	Fabaceae	*Phaseolus vulgaris* L.	red kidney bean	crop
Plantae	dicots of Anthophyta (angiosperms)	Fabaceae	*Arachis hypogaea* L	peanut	crop
Plantae	dicots of Anthophyta (angiosperms)	Nyssaceae	*Camptotheca acuminata* Decaisne	happytree	cultivated
Plantae	dicots of Anthophyta (angiosperms)	Nyssaceae	*C. lowreyana* Li	Lowrey's happytree	cultivated
Plantae	dicots of Anthophyta (angiosperms)	Euphorbiaceae	*Triadica sebifera* (L.) Small	Chinese tallow	invasive
Plantae	dicots of Anthophyta (angiosperms)	Euphorbiaceae	*Cnidoscolus aconitifolius* (Mill.) I.M. Johnst.	chaya	crop
Plantae	dicots of Anthophyta (angiosperms)	Fagaceae	*Quercus texana* Buckley	Nuttall oak	native
Plantae	dicots of Anthophyta (angiosperms)	Fagaceae	*Q. shumardii* Buckley	Shumard oak	native
Plantae	dicots of Anthophyta (angiosperms)	Fagaceae	*Q. michauxii* Nuttall	swamp chestnut oak	native
Plantae	dicots of Anthophyta (angiosperms)	Fagaceae	*Q. acutissima* Carruth.	Sawtooth oak	potentially invasive

Supplementary Table S2 – Results of abnormal seedlings of *Camptotheca* in the greenhouse (germination from the fruits without soaking in water) and the field

Taxon	Experimental Site	Survey Plots	Total Seedlings Surveyed	Percentage of seedlings with one or more abnormal leaves (%)
C. acuminata	Greenhouse (fruits without soaking in water)	93 pots	93	0
	Field	4 plots (1x1 m^2 each)	272	7.9
C. lowreyana 'Hicksii'	Greenhouse (fruits without soaking in water)	12 pots	12	0
	Field	4 plots (1x1 m^2 each)	13	92.3

Supplementary Table S3 – Woody species list in the decapitation pruning experiments

Kingdom	Phyllum/Division	Family	Species	Common Name	Status in North America
Plantae	dicots of Anthophyta (angiosperms)	Nyssaceae	*Camptotheca acuminata* Decaisne	happytree	cultivated
Plantae	dicots of Anthophyta (angiosperms)	Nyssaceae	*C. lowreyana* Li	Lowrey's happytree	cultivated
Plantae	dicots of Anthophyta (angiosperms)	Euphorbiaceae	*Triadica sebifera* (L.) Small	Chinese tallow	invasive
Plantae	dicots of Anthophyta (angiosperms)	Moraceae	*Morus alba* L.	mulberry	native & unwanted
Plantae	dicots of Anthophyta (angiosperms)	Altingiaceae	*Liquidambar styraciflua* L.	sweetgum	native & unwanted
Plantae	dicots of Anthophyta (angiosperms)	Fagaceae	*Q. shumardii* Buckley	Shumard oak	native
Plantae	dicots of Anthophyta (angiosperms)	Rosaceae	*Prunus persica* (L.) Stoles	peach tree	cultivated
Plantae	dicots of Anthophyta (angiosperms)	Aquifoliaceae	*Ilex vomitoria* Sol. Ex Alton	yaupon	native
Plantae	dicots of Anthophyta (angiosperms)	Elaeagnaceae	*Elaeagnus pungens* Thunb.	thorny olive	invasive
Plantae	dicots of Anthophyta (angiosperms)	Adoxaceae	*Sambucus cabadensis* L.	elderberry	native

Supplementary Table S4 – Experimental species list for the extraction and external applications of endocides

Kingdom	Phyllum/Division	Family	Species	Common Name	Status in North America
Plantae	Charophyta (green algae)	Characeae	*Chara vulgaris* L.	muskgrass	Native & unwanted
Plantae	Bryophyta (mosses)	Entodontaceae	*Entodon seductrix* (Hedw.) Müll. Hal.	seductive entodon moss	native & unwanted
Plantae	Bryophyta (mosses)	Polytrichaceae	*Atrichum angustatum* (Bridel) Bruch & Schimper	atrichum moss	native & unwanted
Plantae	Pteridophyta (ferns)	Salviniaceae	*Salvinia molesta* D. S. Mitchell	giant salvinia	invasive
Plantae	Coniferophyta (gymnosperms)	Pinaceae	*Pinus koraiensis* Siebold & Zucc.	Korean pine	ornamental tree
Plantae	dicots of Anthophyta (angiosperms)	Menyanthaceae	*Nymphoides cristata* (Roxb.) Kuntze	crested floating heart	invasive
Plantae	dicots of Anthophyta (angiosperms)	Amaranthaceae	*Amaranthus palmeri* S. Wats.	Palmer's pigweed	invasive
Plantae	dicots of Anthophyta (angiosperms)	Amaranthaceae	*Alternanthera philoxeroides* Griseb.	alligator weed	invasive
Plantae	dicots of Anthophyta (angiosperms)	Cactaceae	*Opuntia ficus-indica* (L.) Mill.	nopal cactus or Indian fig opuntia	invasive
Plantae	dicots of Anthophyta (angiosperms)	Brassicaceae	*Brassica oleracea* L.	broccoli	crop
Plantae	dicots of Anthophyta (angiosperms)	Fabaceae	*Pueraria montana* (Lour.) Merr.	kudzu	invasive
Plantae	dicots of Anthophyta (angiosperms)	Fabaceae	*Phaseolus vulgaris* L.	red kidney bean	crop
Plantae	dicots of Anthophyta (angiosperms)	Fabaceae	*Arachis hypogaea* L.	peanut	crop
Plantae	dicots of Anthophyta (angiosperms)	Euphorbiaceae	*Triadica sebifera* (L.) Small	Chinese tallow	invasive
Plantae	dicots of Anthophyta (angiosperms)	Euphorbiaceae	*Croton capitatus* Michx. var. *lindheimeri* (Engelm. & Gray) Muell.-Arg.	hogwort	native & unwanted
Plantae	dicots of Anthophyta (angiosperms)	Euphorbiaceae	*Cnidoscolus aconitifolius* (Mill.) I.M. Johnst.	chaya	crop
Plantae	dicots of Anthophyta (angiosperms)	Aquifoliaceae	*Ilex vomitoria* Sol. Ex Alton	yaupon	native & unwanted
Plantae	dicots of Anthophyta (angiosperms)	Altingiaceae	*Liquidambar styraciflua* L.	sweetgum	native & unwanted
Plantae	dicots of Anthophyta (angiosperms)	Anacardiaceae	*Schinus terebinthifolius* Raddi	Brazilian pepper tree	invasive
Plantae	dicots of Anthophyta (angiosperms)	Simaroubaceae	*Ailanthus altissima* (P. Mill.) Swingle	tree of heaven	invasive
Plantae	dicots of Anthophyta (angiosperms)	Fagaceae	*Quercus texana* Buckley	Nuttall oak	native
Plantae	dicots of Anthophyta (angiosperms)	Fagaceae	*Q. shumardii* Buckley	Shumard oak	native
Plantae	dicots of Anthophyta (angiosperms)	Fagaceae	*Q. acutissima* Carruth.	sawtooth oak	potentially invasive
Plantae	dicots of Anthophyta (angiosperms)	Oleaceae	*Ligustrum sinense* Lour.	Chinese privet	invasive

Plantae	monocots of Anthophyta (angiosperms)	Araceae	*Pistia stratiotes* L.	water lettuce	invasive
Plantae	monocots of Anthophyta (angiosperms)	Amaryllidaceae	*Allium sativum* L.	garlic	crop
Plantae	monocots of Anthophyta (angiosperms)	Pontederiaceae	*Eichhornia crassipes* (Mart.) Solms	water hyacinth	invasive
Plantae	monocots of Anthophyta (angiosperms)	Poaceae	*Sorghum bicolor* (L.) Moench	sorghum	crop
Plantae	monocots of Anthophyta (angiosperms)	Poaceae	*S. halepense* (L.) Pers.	Johnsongrass	invasive
Plantae	monocots of Anthophyta (angiosperms)	Poaceae	*Arundo donax* L.	giant reed	invasive
Plantae	monocots of Anthophyta (angiosperms)	Poaceae	*Phyllostachys aurea* Carr. ex A. & C. Rivière	golden bamboo	invasive
Plantae	monocots of Anthophyta (angiosperms)	Cyperaceae	*Isolepis prolifera* (Rottb.) R. Br.	proliferating bulrush	invasive
Animalia	Arthropoda (insects)	Formicidae	*Solenopsis invicta* Buren	red imported fire ant	invasive
Animalia	Arthropoda (insects)	Rhinotermitidae	*Reticulitermes flavipes* (Kollar)	subterranean termite	native
Animalia	Arthropoda (insects)	Acrididae	*Schistocerca americana* Drury	American grasshopper	native
Animalia	Arthropoda (insects)	Tenebrionidae	*Tenebrio molitor* L.	mealworm	invasive
Animalia	Arthropoda (insects)	Tenebrionidae	*Zophobas morio* Fabricius	superworm	invasive

Supplementary Table S5 – Experimental species list for the external applications of endocides for selectivity

Kingdom	Phyllum/Division	Family	Species	Common Name	Status in North America
Plantae	Charophyta (green algae)	Characeae	*Chara vulgaris* L.	muskgrass	Native & unwanted
Plantae	Chlorophya (green algae)	Cladophoraceae	*Cladophora* sp.	hair algae	invasive
Plantae	Chlorophya (green algae)	Cladophoraceae	*Pithophora roettleri* (Roth) Wittrock	horse hair algae	invasive
Plantae	Pteridophyta (ferns)	Salviniaceae	*Salvinia molesta* D. S. Mitchell	giant salvinia	invasive
Plantae	Pteridophyta (ferns)	Salviniaceae	*S. minima* Baker	water moss	invasive
Plantae	Pteridophyta (ferns)	Azollaceae	*Azolla caroliniana* Willd.	mosquito fern	native
Plantae	Pteridophyta (ferns)	Dryopteridaceae	*Athyrium filix-femina* (L.) Mertens var. aspenioides (Michaux) Farwell	common lady fern	native
Plantae	Pteridophyta (ferns)	Thelypteridaceae	*Thelypteris kunthi* (Desv.) C.V. Morton	southern shield fern	native
Plantae	Coniferophyta (gymnosperms)	Pinaceae	*Pinus taeda*	loblolly pine	native
Plantae	Coniferophyta (gymnosperms)	Cupressaceae	*Taxododium distichum* (L.) Rich.	baldcypress	native
Plantae	dicots of Anthophyta (angiosperms)	Menyanthaceae	*Nymphoides cristata* (Roxb.) Kuntze	crested floating heart	invasive
Plantae	eudicots of Anthophyta (angiosperms)	Ceratophyllaceae	*Ceratophyllum demersum* L.	coontail	Native & unwanted
Plantae	dicots of Anthophyta (angiosperms)	Fabaceae	*Desmodium* sp.	tick-trefoil	native
Plantae	dicots of Anthophyta (angiosperms)	Rubiaceae	*Cephalanthus occidentalis* L.	buttonbush	native
Plantae	dicots of Anthophyta (angiosperms)	Caprifoliaceae	*Lonicera japonica* Thunb.	Japanese honeysuckle	invasive
Plantae	dicots of Anthophyta (angiosperms)	Onagraceae	*Ludwigia* sp.	Water primrose	native
Plantae	dicots of Anthophyta (angiosperms)	Polygonaceae	*Polygonum* sp.	knotweed	native
Plantae	dicots of Anthophyta (angiosperms)	Convolvulaceae	*Ipomoea pandurata* (L.) G.F.W. Mey	potato vine	native
Plantae	dicots of Anthophyta (angiosperms)	Euphorbiaceae	*Triadica sebifera* (L.) Small	Chinese tallow	invasive
Plantae	dicots of Anthophyta (angiosperms)	Euphorbiaceae	*Acalypha rhomboidea* Raf.	three-seeded mercury	native & unwanted
Plantae	dicots of Anthophyta (angiosperms)	Altingiaceae	*Liquidambar styraciflua* L.	sweetgum	native & unwanted
Plantae	dicots of Anthophyta (angiosperms)	Anacardiaceae	*Rhus aromatica* L.	sumac	native
Plantae	dicots of Anthophyta (angiosperms)	Anacardiaceae	*Toxicodendron radicans* (L.) Kuntze	poison ivy	native
Plantae	dicots of Anthophyta (angiosperms)	Fagaceae	*Quercus texana* Buckley	Nuttall oak	native
Plantae	dicots of Anthophyta (angiosperms)	Fagaceae	*Q. shumardii* Buckley	Shumard oak	native

Plantae	dicots of Anthophyta (angiosperms)	Myricaceae	*Myrica heterophylla* Raf.	waxmyrtle	native
Plantae	dicots of Anthophyta (angiosperms)	Oleaceae	*Ligustrum sinense* Lour.	Chinese privet	invasive
Plantae	dicots of Anthophyta (angiosperms)	Vitaceae	*Vitis rotundifolia* Michx.	muscadine grape	native
Plantae	dicots of Anthophyta (angiosperms)	Ulmaceae	*Planera aquatica* J.F. Gmel.	water elm	native
Plantae	dicots of Anthophyta (angiosperms)	Hydrocharitaceae	*Hydrilla verticillata* (L.f.) Royle	hydrilla	invasive
Plantae	dicots of Anthophyta (angiosperms)	Lentibulariaceae	*Utricularia macrorhiza* LeConte	bladderwort	invasive
Plantae	monocots of Anthophyta (angiosperms)	Pontederiaceae	*Eichhornia crassipes* (Mart.) Solms	water hyacinth	invasive
Plantae	monocots of Anthophyta (angiosperms)	Araceae	*Lemna minuta* Kunth	least duckweed	invasive
Plantae	monocots of Anthophyta (angiosperms)	Araceae	*Wolffia brasiliensis* Weddell	Brazillan watermeal	invasive
Plantae	monocots of Anthophyta (angiosperms)	Araceae	*Pistia stratiotes* L.	water lettuce	invasive
Plantae	monocots of Anthophyta (angiosperms)	Poaceae	*Dichanthelium boscii* (Poir.) Gould & C.A. Clark	Bosc's panicagrass	native
Plantae	monocots of Anthophyta (angiosperms)	Poaceae	*Sorghum bicolor* (L.) Moench	sorghum	crop
Plantae	monocots of Anthophyta (angiosperms)	Poaceae	*S. halepense* (L.) Pers.	Johnsongrass	invasive
Plantae	monocots of Anthophyta (angiosperms)	Poaceae	*Arundo donax* L.	giant reed	invasive
Plantae	monocots of Anthophyta (angiosperms)	Poaceae	*Phyllostachys aurea* Riviere & C. Riviere	golden bamboo	invasive
Plantae	monocots of Anthophyta (angiosperms)	Poaceae	*Cynodon dactylon* (L.) Pers.	bermudagrass	invasive
Plantae	monocots of Anthophyta (angiosperms)	Juncaceae	*Juncus* sp.	rush	native
Plantae	monocots of Anthophyta (angiosperms)	Cyperaceae	*Carex* sp.	sedge	native
Plantae	monocots of Anthophyta (angiosperms)	Cyperaceae	*Isolepis prolifera* (Rottb.) R. Br.	proliferating bulrush	invasive
Plantae	monocots of Anthophyta (angiosperms)	Typhaceae	*Typha latifolia* L.	cattail	native
Plantae	monocots of Anthophyta	Zingiberaceae	*Curcuma longa* L.	turmeric	crop

	(angiosperms)				
Animalia	Arthropoda (insects)	Formicidae	*Solenopsis invicta* Buren	red imported fire ant	invasive
Animalia	Arthropoda (insects)	Formicidae	*Nylanderia fulva* Mayr	Rasberry crazy ant	invasive
Animalia	Arthropoda (insects)	Rhinotermitidae	*Reticulitermes flavipes* (Kollar)	subterranean termite	native
Animalia	Arthropoda (insects)	Tenebrionidae	*Tenebrio molitor* L.	mealworm	invasive
Animalia	Arthropoda (insects)	Tenebrionidae	*Zophobas morio* Fabricius	superworm	invasive

Supplementary Table S6 – Bioactivity of four isolates from *S. molesta* against the producing species

Compound			Activity against *S. molesta*
No.	Name	Structure	
1	(+)-3-Hydroxy-*β*-ionone	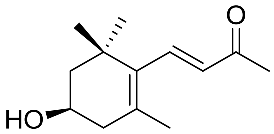	Potent activity
2	(3R,6R,7E)-3-Hydroxy-4,7-megastigmadien-9-one	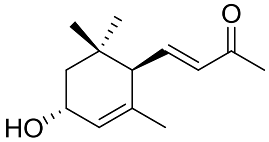	Weak activity
3	Annuionone D	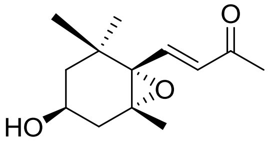	No activity
4	Dehydrovomifoliol	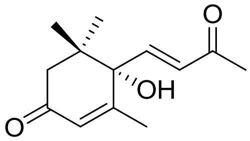	No activity

Supplementary Table S7 – Impacts of *Tenebrio molitor* and *Zophobas morio* extracts on the survival of their larvae

**Extracts**	**Treatment Level**	**Surviving *Tenebrio molitor* Number after 24 h of the Treatment (mean ± s.d.)**			**Surviving *Zophobas morio* Number after 24 h of the Treatment (mean ± s.d.)**	
**Larvae/Adults Lot Information**		Larvae T15813	Larvae T15911	Adults T15911	Larvae Z15721	Larvae Z15911
**Mean Fresh Weight per Worm (mg)**		93.5	79.2	18.5	661.4	204
***Tenebrio molitor* Extracts** (Lot: T15813; Extraction worm number: 550; Extract yield per worm: 2.7 mg)	0 mg	10 ± 0 a	9.7 ± 0.6 a	10 ± 0 a	10 ± 0 a	---
	1 mg	7.3 ± 0.6 b	9.7 ± 0.6 a	10 ± 0 a	10 ± 0 a	---
	2 mg	7 ± 0 b	10 ± 0 a	---	10 ± 0 a	---
	3 mg	6.7 ± 0.6 b	6 ± 1.7 b	---	---	---
	5 mg	---	---	---	---	---
	10 mg	---	---	---	10 ± 0 a	---
	20 mg	---	---	---	8.3 ± 0.8 b	---
***Tenebrio molitor* Extracts** (Lot: T15911; Extraction worm number: 1,500; Extract yield per worm: 3.8 mg)	0 mg	---	---	10 ± 0 a	---	10 ± 0 a
	1 mg	---	---	9.7 ± 0.6 a	---	---
	2 mg	---	---	---	---	---
	3 mg	---	---	---	---	---
	5 mg	---	---	4.7 ± 1.5 b	---	9.7 ± 0.6 a
	10 mg	---	---	---	---	7 ± 1.7 b
	20 mg	---	---	---	---	6 ± 1.7 b
***Zophobas morio* Extracts** (Lot: Z15813; Extraction worm number: 100; Extract yield per worm: 20.3 mg)	0 mg	10 ± 0 a	---	10 ± 0 a	10 ± 0 a	10 ± 0 a
	1 mg	8.3 ± 2.9 ab	---	5 ± 3.6 b	10 ± 0 a	---
	2 mg	4.3 ± 4 b	---	---	10 ± 0 a	---
	3 mg	8 ± 0 ab	---	---	---	---
	5 mg	---	---	---	---	2 ± 1 b
	10 mg	---	---	---	9.7 ± 0.6 a	0.3 ± 0.1 c
	20 mg	---	---	---	6.3 ± 1.5 b	0.3 ± 0.1 c
***Zophobas morio* Extracts** (Lot: Z15911; Extraction worm number: 300; Extract yield per worm: 8.7 mg)	0 mg	---	10 ± 0 a	10 ± 0 a	---	10 ± 0 a
	1 mg	---	9.7 ± 0.6 a	8.7 ± 1.5 a	---	---
	2 mg	---	8.7 ± 1.5 a	---	---	---
	3 mg	---	9.7 ± 0.6 a	---	---	---
	5 mg	---	---	2.7 ± 2.5 b	---	7.3 ± 1.5 ab
	10 mg	---	---	---	---	6 ± 1.7 b
	20 mg	---	---	---	---	1.3 ± 2.3 c

--- no data. Significant differences among means of treatment and control are indicated by different letters ($P < 0.05$).

References

1 Li, S., Wang, P., Deng, G., Yuan, W. & Su, Z. Cytotoxic compounds from invasive giant salvinia (*Salvinia molesta*) against human tumor cells. *Bioorg. Med. Chem. Lett.* **23**, 6682-6687, doi: 10.1016/j.bmcl.2013.10.040 (2013).

2 Burks, K. C. *Nymphoides cristata*-snowflakes in sunny Florida. *Aquatics*, 8-10 (2002).

3 Willey, L. N. & Langeland, K. A. in *Aquatic Weeds: Crested Floating Heart (Nymphoides cristata)* (ed University of Florida IFAS Extension) (2011).

4 Ascunce, M. S. *et al.* Global invasion history of the fire ant *Solenopsis invicta*. *Science* **331**, 1066-1068, doi:10.1126/science.1198734 (2011).

5 Chen, J., Cantrell, C. L., Shang, H. W. & Rojas, M. G. Piperideine alkaloids from the poison gland of the red imported fire ant (Hymenoptera: Formicidae). *J. Agric. Food Chem.* **57**, 3128-3133, doi: 10.1021/jf803561y (2009).

6 Hoffman, D. R. Ant Venoms. *Curr. Opin. Allergy Clin. Immunol.* **10**, 342-345, doi: 10.1097/ACI.0b013e328339f325 (2010).

7 Morrison, L. W., Porter, S. D., Daniels, E. & Korzukhin, M. D. Potential global range expansion of the invasive fire ant, *Solenopsis invicia*. *Biol. Invas.* **6**, 183-191 (2004).
